# Mechanically Robust Hybrid Gel Beads Loaded with “Naked” Palladium Nanoparticles as Efficient, Reusable, and Sustainable Catalysts for the Suzuki–Miyaura Reaction

**DOI:** 10.1021/acssuschemeng.2c05484

**Published:** 2023-01-24

**Authors:** Matteo Albino, Thomas J. Burden, Carmen C. Piras, Adrian C. Whitwood, Ian J. S. Fairlamb, David K. Smith

**Affiliations:** Department of Chemistry, University of York, Heslington, York YO10 5DD, U.K.

**Keywords:** catalysis, gel, gelator, nanoparticles, palladium, Suzuki−Miyaura

## Abstract

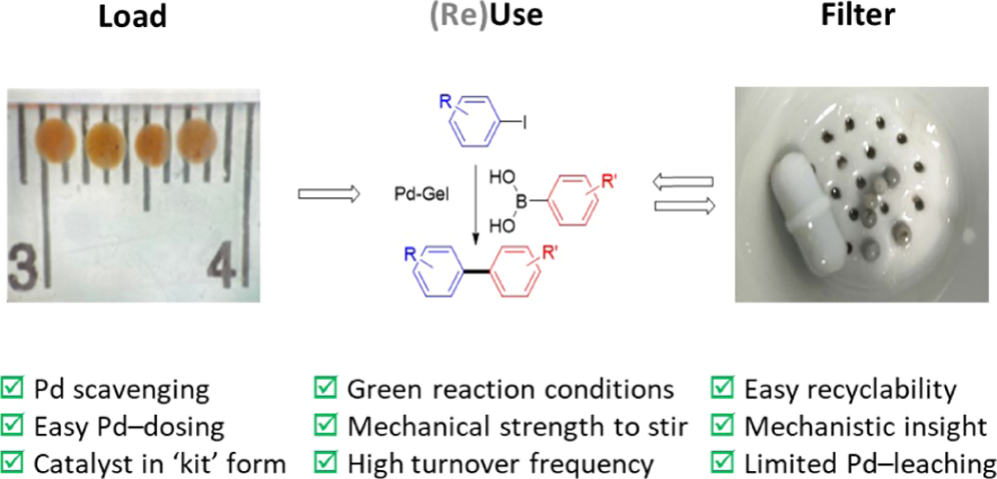

The increase in demand for Pd and its low abundance pose a significant threat to its future availability, rendering research into more sustainable Pd-based technologies essential. Herein, we report Pd scavenging mechanically robust hybrid gel beads composed of agarose, a polymer gelator (PG), and an active low-molecular-weight gelator (LMWG) based on 1,3:2,4-dibenzylidenesorbitol (DBS), **DBS-CONHNH**_**2**_. The robustness of the PG and the ability of the LMWG to reduce Pd(II) *in situ* to generate naked Pd(0) nanoparticles (PdNPs) combine within these gel beads to give them potential as practical catalysts for Suzuki–Miyaura cross-coupling reactions. The optimized gel beads demonstrate good reusability, green metrics, and most importantly the ability to sustain stirring, improving reaction times and energy consumption compared to previous examples. In contrast to previous reports, the leaching of palladium from these next-generation beads is almost completely eliminated. Additionally, for the first time, a detailed investigation of these Pd-loaded gel beads explains precisely how the nanoparticles are formed *in situ* without a stabilizing ligand. Further, detailed catalytic investigations demonstrate that catalysis occurs within the gel beads. Hence, these beads can essentially be considered as robust “nonligated” heterogeneous PdNP catalysts. Given the challenges in developing ligand-free, naked Pd nanoparticles as stable catalysts, these gel beads may have future potential for the development of easily used systems to perform chemical reactions in “kit” form.

## Abstract 2

Self-assembled gel beads reclaim palladium from aqueous solution, and the resulting Pd-nanoparticle-loaded beads act as recyclable catalysts for Suzuki−Miyaura cross-couplings under environmentally benign conditions.

## Introduction

After the discovery and commercialization of Pd-catalyzed cross-coupling reactions at the end of the 20th century,^1−10^ there has been a steep increase in both the demand for, and the price of, palladium.^11^ In an analysis of synthetic methodologies in medicinal chemistry, Suzuki–Miyaura cross-coupling (SMCC) reactions alone were the fifth most widely used transformation, demonstrating the high dependence of modern society on this element.^12^ Economic factors are a significant concern in terms of use of this element and the cost to society. More importantly, long-term sustainability is a worry, considering the low abundance of Pd, meaning current consumption rates pose a serious threat to future supplies.^13^

In recent years, there has therefore been a significant drive for research into both alternative Pd-independent synthetic methodologies, such as the utilization of earth-abundant metals,^14−16^ and more sustainable Pd-utilizing technologies.^11,17−19^ One method to achieve the latter is to develop systems that, in addition to reclaiming waste Pd, can subsequently utilize it, with minimal input of energy, for desired transformations. Examples include biological remediation using bacteria and fungi,^20−22^ functionalized polymers,^23^ and MOFs.^24^ A wide range of such nanostructured supports have then been explored with regard to Pd-nanoparticle mediated catalysis.^25^ A class of materials that are also very effective for environmental remediation and catalysis are gels:^26^ these materials have the potential to uptake metal ions under ambient conditions and store them inside their gel networks, often without influencing their catalytic activity, with the resulting metal-loaded gels being able to be used directly in reactions under ambient, environmentally friendly conditions. An elegant recent example, specific to Pd, made use of an **Alkyne–PVA** polymer gel.^27^ This system relied on the ability of Pd(II) to catalyze homocoupling between alkynes present on different polymer chains, crosslinking them and hence forming a three-dimensional (3D) chemically-crosslinked gel network. The Pd(II) ions trapped within the gel could then be reduced to palladium nanoparticles (PdNPs) using NaBH_4_ to form a polymer metallogel able to catalyze SMCC reactions in an ethanol–water mixture in less than an hour using ppm levels of catalyst.

Indeed, there has been considerable general interest in using polymeric materials as supports for palladium nanoparticles, even in the absence of Pd remediation.^28^ In early work, Kobayashi and co-workers incarcerated PdNPs within a covalently crosslinked polymeric material and went on to demonstrate the capacity for Suzuki–Miyaura catalysis.^29,30^ These systems included phosphine ligands to assist in stabilizing the Pd. In later work, this phosphine was also built into the polymer backbone.^31^ Yamada and co-workers also made use of a covalently crosslinked polymer composite with appended phosphine ligands and an interior microgel structure to deliver Pd catalysts into Suzuki–Miyaura reactions.^32^ Other research groups built on this crosslinked polymer stabilization approach.^33,34^ Rather than using covalently crosslinked synthetic polymers, Quignard and co-workers employed alginate crosslinked with metal ions as a support for palladium nanoparticle catalysts produced *in situ*,^35^ and went on to demonstrate that the co-cation present in the alginate polymer gel could also impact upon the catalytic outcome.^36^ Nanostructured polyamines have also been used as an effective support for palladium-catalyzed reactions.^37,38^ Alternative to polymers and polymer gels, silica-based gels have also been employed as supports for PdNP catalysts.^39,40^ and in some cases, the support can also act as an *in situ* reducing agent to produce the PdNPs from Pd(II), with their use being demonstrated under flow synthesis conditions.^41^

In contrast to polymer gels, supramolecular gels based on low-molecular-weight gelators (LMWGs) can be easily and reversibly assembled and readily tuned to incorporate active functional groups. They have become of much interest for applications in catalysis as a result of their high activities, ease of recycling, and their green and sustainable credentials.^42−45^ Early reports incorporated Pd-binding ligands into such materials and then used the resulting bulk gels to catalyze cross-coupling reactions.^46,47^ Going beyond simple Pd ligation, Maity and Maitra used an external reductant to create PdNPs in gels that could catalyze Suzuki–Miyaura reactions.^48^ Inspired by this work, we developed an active LMWG, **DBS-CONHNH**_**2**_, not only capable of removing precious-metal cations from waste water down to ppb levels but also able to spontaneously reduce them *in situ* to their elemental form, storing them as NPs inside its network.^49^ When loaded with Pd(II), the resulting gel with embedded Pd(0)-NPs has been used for Suzuki–Miyaura, Heck, and Sonogashira cross-couplings with excellent yields, good functional group tolerability, and green reaction conditions.^50−52^ Recently, Haldar and co-workers used a similar strategy to develop a self-assembled organogel in toluene, which accumulates PdNPs and catalyzes Suzuki–Miyaura cross-coupling reactions when brought into contact with an aqueous solution of reagents.^53^

**Figure 1:**
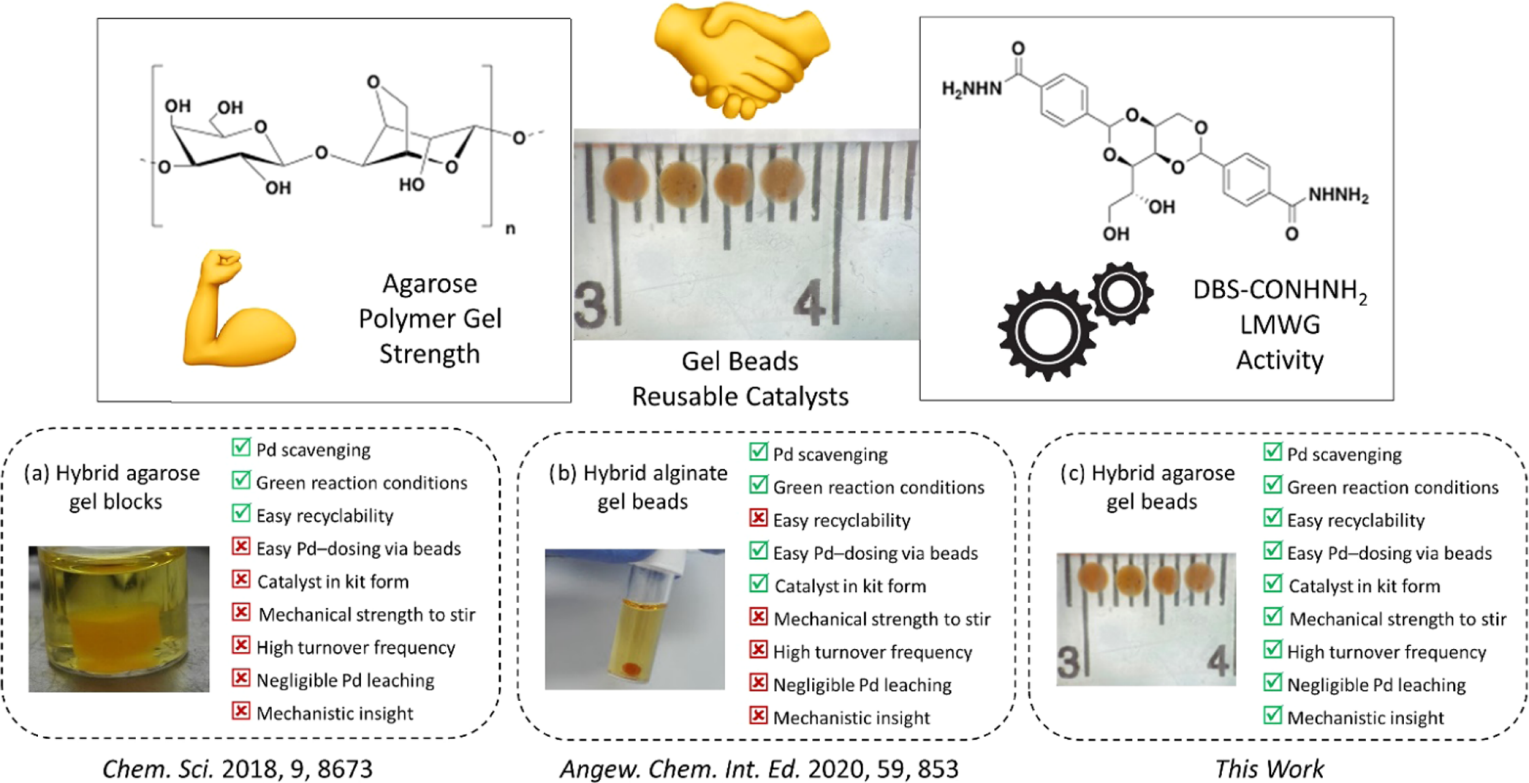
Composition of agarose/**DBS-CONHNH**_**2**_ hybrid hydrogels used in this work for Pd scavenging and catalysis, and a comparison between the gels previously reported by our group for this application (boxes (a) and (b))^50,52^ and this work (box (c)). The photographs in boxes (a) and (b) were produced previously by the research group and published open access in ref (50−52) under CC-BY license, and therefore have permission to be reproduced.

As for many LMWGs, however, **DBS-CONHNH**_**2**_ suffers from low mechanical stability. A widely used method to improve the strength of gels is to form hybrid gels composed of two gelators—for example, using an LMWG to give function and a polymer gelator (PG) to provide robustness.^54^ Hence, to allow for more facile separation and reusability of the catalyst, we previously combined this LMWG with agarose to make blocks^50,51^ or calcium alginate to make beads (Figure 1).^52^ Both of these sugar-based PGs are biodegradable and sustainable. This approach improved the practicality of the catalyst, but the alginate bead formulation was still too weak to sustain reaction stirring, causing long reaction times, limited by diffusion. Additionally, they were too mechanically weak to simply remove from the reaction by filtration and then dose into another subsequent reaction. They also suffered very significantly from Pd leaching, which cast doubt onto the gel phase heterogeneous nature of the catalytically active species.

Hybrid PG/LMWG gel systems open the possibility of imposing shape/morphology on gels, such as beads.^52,55^ For the work in this paper, we envisioned that the use of agarose as a PG, and employing a higher concentration compared to our previous gel blocks, might facilitate the formation of well-defined beads and prevent the mechanical degradation caused by stirring without compromising the activity of our catalyst. Using catalyst-loaded beads allows for facile precise dosing of Pd compared to using gel blocks since each bead contains a discrete, known amount of Pd. Furthermore, such beads can potentially be made at scale using flow chemistry methods. This approach is therefore potentially amenable to the development of easy-to-use reaction kits for SMCC methodology. Very pleasingly, we report here that the new hybrid beads loaded with Pd were able to sustain reaction stirring without any visible mechanical degradation, while catalyzing SMCC reactions. We report the process optimization and scope for this new system, demonstrating its superior performance compared to previous generation counterparts. Furthermore, to obtain a holistic understanding of these new systems, we also report for the first time a detailed mechanistic understanding of both the Pd-gel-loading process and the gel-mediated Pd-catalyzed SMCC reactions.

## Results and Discussion

### Fabrication and Characterization of Gel Beads

The LMWG **DBS-CONHNH**_**2**_ was synthesized as previously reported,^56^ while agarose is commercially available. Both gels are formed using a heat–cool cycle, and therefore gelation can be triggered simultaneously when heating (and then cooling) a solution of both gelators. Hybrid gel beads can be manufactured by the dropwise addition of hot aqueous gelator solution into ice-cold paraffin oil, with the drop volume controlling the size of bead formed. We targeted gel beads with diameters of *ca.* 2 mm as they could be easily handled in terms of physical manipulation, reaction dosing, and bead recycling.

**Figure 2:**
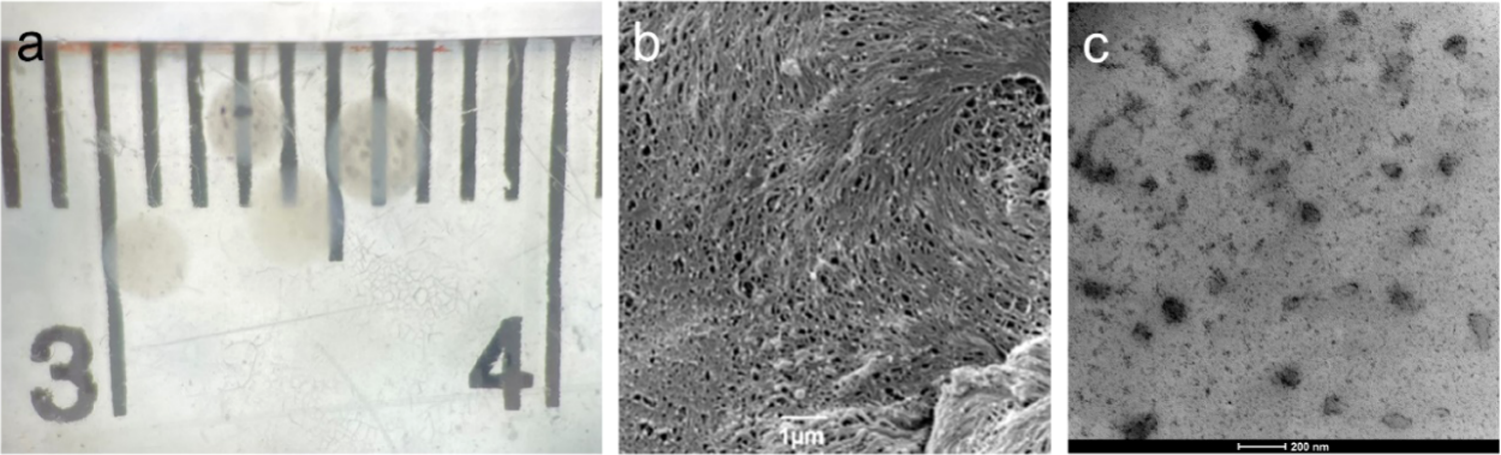
(a) Hybrid beads under an optical microscope. (b) SEM image of gel fibers and (c) TEM image of PdNPs.

The characterization of the gel beads in this work is consistent with our previous report, which made use of this type of bead in a different context.^55^ The formulation employed 0.3% wt/vol of the **DBS-CONHNH**_**2**_ LMWG and 1.0% wt/vol of the agarose PG. The loading of the LMWG was chosen because at lower loadings, this system does not form effective gels, while at higher loadings, it does not completely dissolve and the gels formed are somewhat homogeneous. Given the LMWG directs PdNP assembly, this choice therefore controls the Pd loading level of the gel beads. The loading of PG was chosen to optimize the mechanical strength of the hybrid gel and enable bead formation as previously described—enhancing stiffness is the primary role played by the agarose network in these hybrid gels.^55^ A PG loading of 1.0% wt/vol was chosen, which is a significant increase over the 0.45% wt/vol used in our previous report of Pd-loaded hybrid hydrogels.^50^ Macroscopically, the beads were spherical in shape and had a diameter of *ca.* 2 mm (Figure 2a), in good agreement with the drop volume of 5 μL, although some small variation in size was observed. Thermal stability, evaluated *via* a simple tube-inversion method on equivalent gels made in vials, indicated a thermal stability (*T*_gel_ value) of *ca.* 99 °C for the hybrid gel—slightly more than the individual LMWG or PG (Table S1). The stiffness of the gels was significantly increased in the hybrid system as indicated by oscillatory rheology using parallel plate geometry on equivalent gels formed in vials, with the hybrid gel at this loading having a *G*′ value of 12,000 ± 100 Pa, much larger than either individual component (Table S7). This suggests that the two networks interpenetrate, and support and reinforce one another.^57^

On the molecular scale, infrared spectroscopy on the gel beads was used to reveal the presence of intermolecular interactions between the two gelators. There was some broadening of the bands corresponding to O–H and N–H stretching frequencies, and furthermore, there was a slight shift in the carbonyl stretching frequency of the **DBS-CONHNH**_**2**_ from 1638 to 1645 cm^–1^, suggesting a degree of noncovalent interaction between the two interpenetrated gel networks (Figures S22, S24, and S26). On the nanoscale, scanning electron microscopy clearly indicated the self-assembled nanofibrillar nature of the interior of the gel beads (Figure 2b). There was no evidence of microscale phase separation between LMWG and PG domains, and the homogeneous fibrillar nature of the gels suggests that, in agreement with our previous work on such materials,^55^ the two networks are interpenetrated with one another throughout the gel.

^1^H NMR experiments provide further insight into the gel networks in the hybrid beads. Initially, a ^1^H NMR spectrum of gel beads in D_2_O (Figure S18) was used to indicate that the LMWG was fully in the solid-like self-assembled state in the gel beads, with no characteristic signals being observed for **DBS-CONHNH**_**2**_. To quantify the amount of LMWG present in the hybrid beads, ten 5 μL beads were dried and completely dissolved in DMSO-*d*_6_. Using a known amount of acetonitrile as an internal standard, it was estimated that 87% of the LMWG used during the fabrication process was incorporated into the beads (Figure S21), indicating the effective incorporation of the LMWG into the gel beads. The hydrothermal stability of the hybrid beads was also evaluated by NMR methods. The beads, manufactured using D_2_O as a solvent, were placed in an NMR tube containing D_2_O and a DMSO internal standard. When the temperature was increased to 90 °C, the beads demonstrated reasonable hydrothermal stability with the amount of LMWG leaching reaching a maximum of 38% after 1 h (Figures S19 and S20 and Table S6).

### Pd Loading into Gel Beads

Next, we monitored the metal uptake achieved by the gel beads—specifically comparing the hybrid gel beads with an agarose-only control. Forty hybrid beads and 40 agarose beads (all made with 5 μL droplets) were each exposed to 3 mL of slightly acidified aqueous 5 mM PdCl_2_ solution. The absorbance of the solution was monitored over 72 h to determine the amount of Pd absorbed in each case. It was found that both gels have similar behaviors with the maximum rate of absorption in the first 6 h, slowly plateauing off within the first 24 h (Table S3). In this way, the beads are simply and effectively able to remediate Pd from Pd-containing aqueous waste—of importance in sustainable chemistry in terms of Pd recycling and reuse.^11,17−19^ The final amount of Pd taken up is 10.0 ± 0.7 μmol mL^–1^ (of gel) and 10.2 ± 1.1 μmol mL^–1^ for the hybrid and agarose beads, respectively (Figure S2). This corresponds to 50 ± 3 and 50 ± 5 nmol per bead for the hybrid and agarose beads, respectively. However, although the amount of Pd taken into the beads is similar in each case, its final fate is very different—the agarose gel beads simply retained the typical pale yellow color of the PdCl_2_ solution, whereas the hybrid beads turned dark orange-brown, clearly signifying a change in the oxidation state of the Pd in the latter case (Figure S3). This change has previously been ascribed to the reduction of Pd(II) to form Pd(0) nanoparticles (NPs).^50−52^ This process is vital for endowing the gel beads with catalytic activity (see below).

The metal loading is fractionally lower compared to the first-generation hybrid gel beads based on alginate/LMWG that sequestered 10.8 μmol mL^–1^ (of gel).^52^ In the first-generation gel beads containing alginate, the systems had a core–shell form, whereas in these agarose-containing systems, the two networks are formed simultaneously on cooling and are therefore fully interpenetrated and interwoven. In the case of these interwoven networks, the two gelators can interact as demonstrated by the IR evidence. We suggest this might slightly reduce the availability of the acylhydrazide moieties for the reduction of Pd(II) (see discussion below).

On the nanoscale, TEM images of the gel beads (Figure 2c) revealed that most of the PdNPs formed had diameter ≤ 3 nm (Table S4)—similar to what was observed in the alginate hybrid gel beads.^52^ However, this was smaller than that previously observed in hybrid gel samples with a lower loading of agarose,^50^ suggesting that the higher PG loading may help restrain the size to which the PdNPs can grow. Surprisingly, together with larger aggregates, there was also some evidence of PdNP formation inside the agarose-only beads (Figures S13–S15 and Table S5). However, the lack of color change means they cannot be definitively assigned as Pd(0), and they may well be based on Pd(II).

IR spectroscopy revealed a significant shift in a peak in the O–H and N–H stretching region from 3295 to 3361 cm^–1^ upon addition of Pd (Figures S23, S25, and S27). There is also a significant change in the carbonyl stretch with a new peak emerging at 1723 cm^–1^. We propose that the species formed upon the oxidation of **DBS-CONHNH**_**2**_ and/or coordination to the metal could be reasonable explanations for these changes, and this led us to consider in more detail the processes occurring on loading precious metals into these gels.

Although we have worked extensively with precious-metal-loaded gels based on **DBS-CONHNH**_**2**_,^49−52,58−60^ we have not previously confirmed the details of the chemical process that occurs upon reduction of the precious metal. Specifically, we wanted to understand here the impact this process has on the gel network and gain some insight into the environment surrounding the metal in the Pd-loaded gels as this may affect catalytic performance. Electron microscopy revealed that the NPs were found primarily in the proximity of the gel fibers, where the acylhydrazide moieties are expected to reside, in agreement with the view that these functional groups play a chemically active role in the Pd-loading process. Furthermore, calculating the uptake of Pd into the gel indicates that 10.0 μmol of Pd is taken up by a theoretical loading of 6.3 μmol of **DBS-CONHNH**_**2**_, which corresponds to 12.6 μmol of acylhydrazide units. This 0.8:1 molar ratio of Pd:acylhydrazide indicates a rough molar equivalence between the species, particularly given that NMR evidence (see above) indicated that in reality, 87% of the total possible LMWG was incorporated into the gel beads (and that some may be inaccessible or interacting with the agarose). This therefore suggests that the acylhydrazide is intimately involved in the palladium uptake/reduction process.

Research from Yates and co-workers on simple acyl hydrazides suggested that acylhydrazide oxidation occurs *via* the loss of N_2_ to form a radical intermediate, that they suggested was subsequently oxidized to form an acyl cation, which is then attacked by nucleophiles—in their case, methanol, giving rise to an ester product.^61^ Later developments from the same group confirmed the importance of the acyl radical intermediate but could not find experimental evidence of the acyl cation.^62^ Instead, they proposed that the nucleophilic methanol, when present in excess as solvent, reacted *via* nucleophilic substitution with the diimide, which is a precursor to the acyl radical, that is formed in the first step of oxidation of the acylhydrazide. However, this previous work did not apply precious-metal salts as oxidants. In our hydrogels, the most commonly encountered nucleophile is water, and therefore we might expect, assuming such an oxidative process can be driven by Pd(II) in our gels, that on Pd loading, **DBS-CONHNH**_**2**_ would be initially oxidized to the diimide **DBS-CON=NH**, which would then be converted to **DBS-CO**_**2**_**H***in situvia* nucleophilic attack of water. Interestingly, the proposed end-product, **DBS-CO**_**2**_**H**, is a well-known supramolecular hydrogelator in its own right and might be expected to retain its gel-type properties.^63^

To unambiguously determine what is occurring in our **DBS-CONHNH**_**2**_ gels, we performed ^1^H NMR experiments (Figures S16 and S17). First, the spectrum of the dried hybrid-xerogel loaded with PdNPs was recorded in DMSO-*d*_6_. This was then separated into two samples, one spiked with **DBS-CONHNH**_**2**_ and one with **DBS-CO**_**2**_**H**. The chemical shifts of the species formed upon **DBS-CONHNH**_**2**_ oxidation exactly matched those of **DBS-CO**_**2**_**H** indicative that the predicted conversion had taken place. Since **DBS-CONHNH**_**2**_ gelation is pH-independent, while **DBS-CO**_**2**_**H** only forms stable gels at pH values below its p*K*_a_ value of 5.4,^64^ we reasoned that by exposing the oxidized xerogel to a solution of NaOD in D_2_O, we would disassemble the **DBS-CO**_**2**_**H** and dissolve it into the aqueous phase. This is indeed what was observed, with the chemical shift of the species dissolved from the xerogel matching an NMR spectrum of **DBS-CO**_**2**_**H** in NaOD/D_2_O.

For the first time, we can therefore confidently assign the metal-loading process as occurring with conversion of **DBS-CONHNH**_**2**_ to **DBS-CO**_**2**_**H**. The fact that both of these species form effective hydrogels means this process can take place without significant loss of gel materials behavior. These observations are also informative when investigating Pd speciation, of relevance to catalytic performance (see below), as they signify that the NPs will be ligated (if at all) by carboxylic acid moieties, which are poor ligands for Pd(0), which can therefore most likely be considered as effectively naked PdNPs.

### Gel Bead-Catalyzed Suzuki–Miyaura Cross-Coupling Reactions

With the detailed characterization of the gel beads in hand, we commenced SMCC reaction optimization. Having previously obtained good results for such reactions in benign conditions consisting of an ethanol–water solvent mixture and K_2_CO_3_ as base,^50,52^ we decided to utilize similar reaction conditions. In our previous work, we had determined that stronger bases, such as KOH, tended to damage the gels, while alternative mild bases such as Cs_2_CO_3_ offered no advantage. Reaction screening was performed on a 5 mL scale at a temperature of 50 °C, using 0.20 mmol of 4′-iodoacetophenone, **a**, 0.24 mmol of 4-tolylboronic acid, **1**, 0.40 mmol of K_2_CO_3_, and four hybrid gel beads, which is equivalent to 0.1 mol % Pd (Table 1). The product was formed with 99% conversion in 1 h and was isolated in 82% yield. Interestingly, almost identical results were obtained with a lower catalyst loading of 0.05 mol % (two hybrid gel beads, entry 8). Hence, this catalyst loading was taken forward.

**Table 1:**
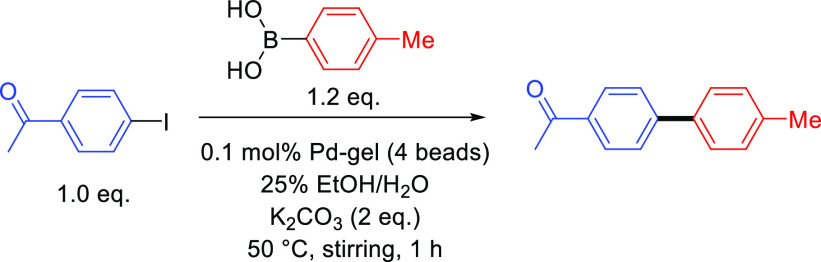
Optimization of Reaction Conditions for Suzuki–Miyaura Cross-Coupling (SMCC) Reaction

entry	deviation from above	conversion^a^ (%)
1	none	99 (82)^b^
2	Pd-loaded agarose-only beads	0
3	Pd-free hybrid beads	0
4	no stirring	20
5	room temperature (25 °C)	40
6	100% EtOH	7
7	100% water	3
8	0.05 mol % Pd gel	99 (83)^b^

a Conversions calculated by NMR analysis.

b Isolated yield after purification.

The solvent proved to be the most influential factor, with very poor conversion being obtained if pure ethanol or pure water were used (entries 6 and 7, respectively). Problems with the solubilization of the base in ethanol, and the solubilization of substrates in water, were proposed to limit reaction in these solvents. Finally, the control experiments (entries 2–4) very pleasingly highlight the synergism between the Pd; the agarose; and the **DBS-CONHNH**_**2**_. Both the presence of the LMWG and the Pd are essential for the beads to be catalytically active. The robust agarose PG is required for stirring to be introduced, significantly increasing the rate of reaction, as highlighted by the significantly reduced conversion when no stirring was applied (entry 4). This constitutes a major step forward in terms of turnover frequency compared with our previous alginate gel beads, for which stirring was impossible,^52^ and indicates how tuning the choice of gel can directly impact the engineering of a hybrid gel system targeted at a specific application.

A smaller optimization was then performed on aryl bromides and aryl chlorides. Since these are notoriously harder to activate than iodides, the catalyst loading was kept at 0.10 mol % (12 beads) and the temperature was raised to 70 °C, as this significantly increased the rate of reaction, without damaging the beads. To achieve effective product yields, reaction times extended a little for aryl bromides (5.5 h, 90%), and as expected, for aryl chlorides became significantly more extended (36 h, 63%). Nonetheless, this success of these reactions was still very pleasing given the benign reaction conditions. Our previous work with similar systems using unactivated aryl chlorides showed low reactivity^50^—this is not surprising as poor reactivity of aryl chlorides is well known for systems incorporating naked Pd nanoparticles.^65^

**Figure 3:**
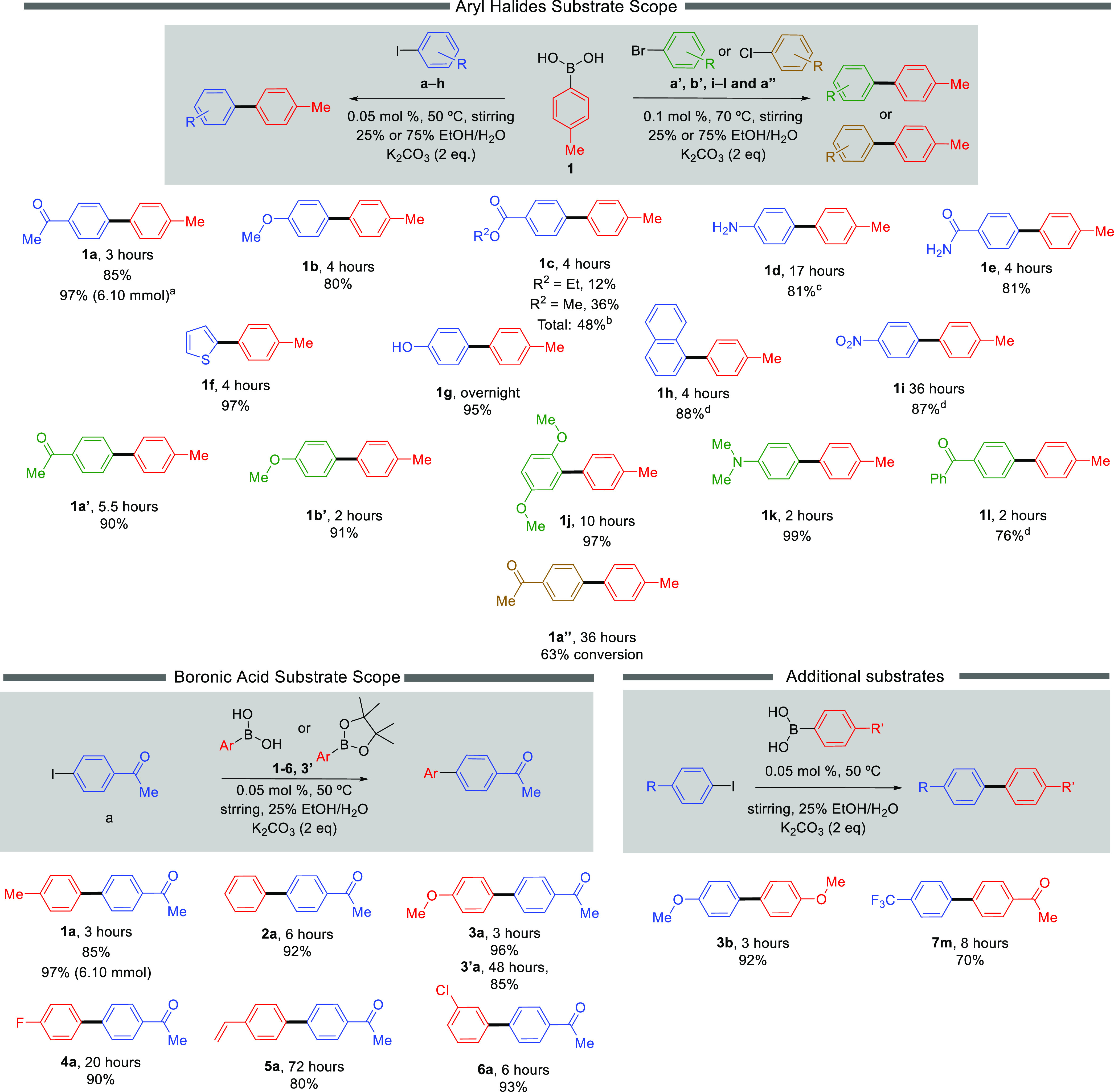
Substrate scope of SMCC reactions explored with the agarose/**DBS-CONHNH**_**2**_ gel beads in this study. ^a^Reaction carried out with 0.03 mol % Pd-gel. ^b^Trans-esterification caused a mixture of products that were not isolated. ^c^The reaction was carried out with 0.60 mol % Pd-gel (24 beads) on a 5 mL scale using 0.20 mmol of aryl iodide, 0.24 mmol of boronic acid, and 0.40 mmol of K_2_CO_3_. ^d^75% EtOH/H_2_O was used.

Satisfied with the outcomes of optimization, the standard coupling conditions were then applied to a range of aryl iodide and bromide substrates to demonstrate the scope of this methodology (Figure 3). In some cases, if the solubility of substrates was found to be problematic, the ethanol content of the solvent was raised to 75%. The Pd-gel catalyst was compatible with electron-donating and electron-withdrawing groups on the aryl halide, giving quantitative conversion, and very good to excellent isolated yields. Methyl ester substituents underwent some hydrolysis and trans-esterification to ethyl esters as a result of the basic conditions and use of aqueous ethanol as solvent. Importantly, in all standard cases, the reaction required no intensive purification, with removal of the slight excess of boronic acid being achieved by washing the organic layer with 1 M NaOH_aq_, once extracted from the reaction mixture. As such, this methodology is robust and operationally simple.

To further demonstrate the applicability of our method the standard reaction was scaled up to the gram scale (6.1 mmol scale). To achieve this, we employed a catalyst loading of *ca*. 0.03 mol % (48 beads), and using this methodology, 1.22 g of **1a** was obtained, in a very pleasing 97% yield. Product purification remained trivial.

**Figure 4:**
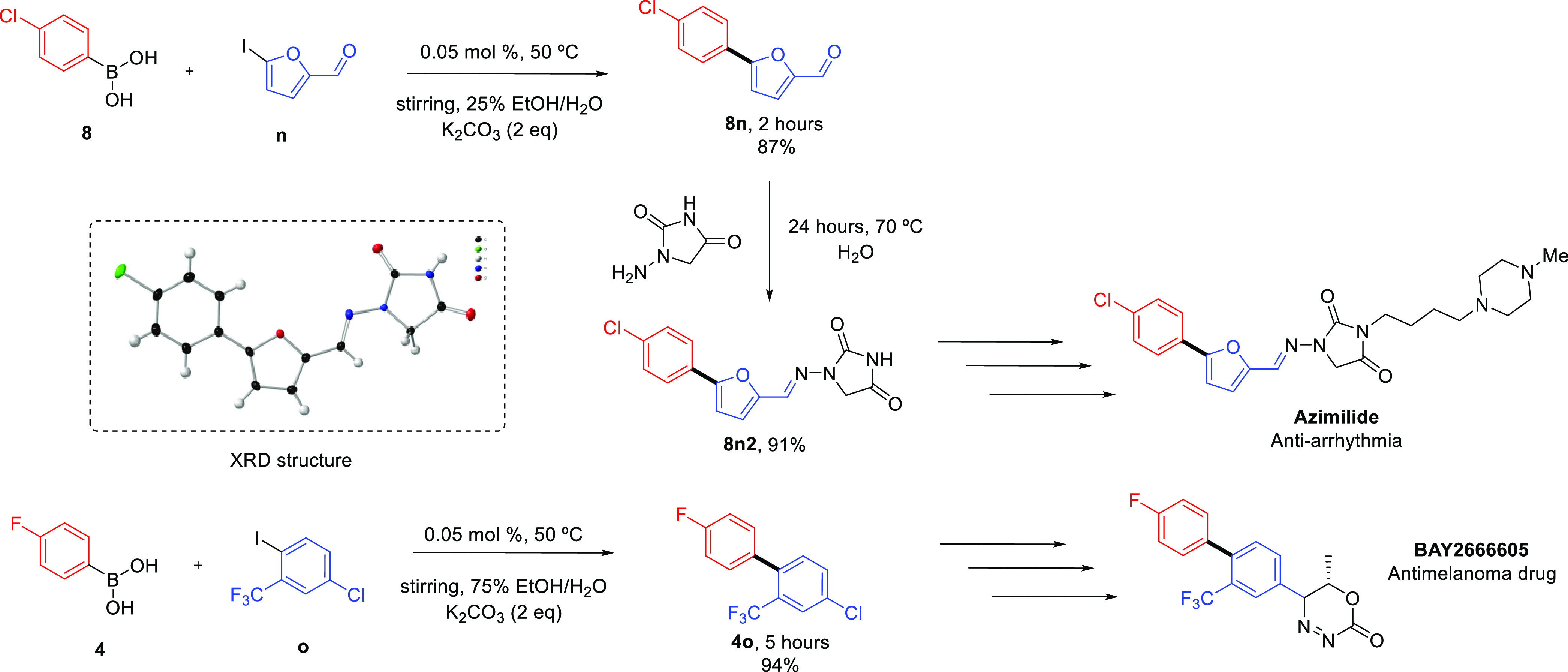
Synthesis of pharmaceutical precursors **8n** and **4o**, including the X-ray crystal structure of compound **8n2**.

In addition, we targeted the synthesis of pharmaceutically relevant substrates (Figure 4). Compound **8n**, a precursor to the anti-arrythmia drug Azimilide,^66^ was obtained in very good yield in just 2 h. Similarly, compound **4o**, which is structurally related to the proposed antimelanoma drug BAY2666605^67^ was obtained in 5 h in excellent yields. It is also interesting to note the aryl halide reagent in this case possesses an *ortho*-CF_3_ group, proving that our catalyst system retains its activity even in the presence of sterically hindered substrates.

The synthesis of compound **2a** allows us to draw a direct comparison between this hybrid gel bead catalyst system and the hybrid agarose/**DBS-CONHNH**_**2**_ gel blocks previously studied for the same transformation.^50^ The gel block exhibited a turnover number (TON) of 100 and a turnover frequency (TOF) of 5.4 h^–1^, as determined on reaction conversion (based on total Pd loading). This new gel bead catalyst had a TON of 2000 and a TOF of 333 h^–1^ at reaction conversion (based on total Pd loading), 1 and 2 orders of magnitude larger, respectively. This demonstrates the clear advantage of formulating the gel as beads rather than applying a simple gel block, resulting from the greater surface area of the gel beads giving rise to more effective substrate access to the catalytic PdNPs within the gel network.

Previous reports of the agarose/**DBS-CONHNH**_**2**_ gel blocks and alginate/**DBS-CONHNH**_**2**_ gel beads demonstrated some reusability of the catalyst, but the alginate hybrid beads were not robust enough to be easily removed from the reaction mixture by filtration.^50,52^ In this case, with the agarose/**DBS-CONHNH**_**2**_ beads, we found that the catalyst only suffered a small loss in activity when reused for the reaction between compounds **1** and **a** to yield **1a**. Indeed, the first two reactions run under these conditions reached completion, while the third run reached 50% conversion after 5 h. Longer reaction times would have likely improved the conversion further. After each reaction, the gel beads can simply be filtered out, washed with dichloromethane and water, and then reused (Table 2). It was therefore much easier to recycle the beads than the fragile hybrid gel beads using alginate.^52^ We note that washing with dichloromethane is not the most sustainable strategy; however, its replacement in process chemistry can be very challenging.^68^ We considered ethyl acetate, but it is not the best extraction solvent to use in the basic aqueous conditions of a Suzuki reaction as it can hydrolyze to difficult-to-remove products. We tested diethyl ether as an alternative; however, this was less effective.

**Table 2:** Reaction of 4′-Iodoacetophenone and 4-Tolylboronic Acid Using Recycled Gel Beads

run	time (h)	conversion (%)^a^
1	3	100 (85)^b^
2	5	100
3	5	50

a Conversion percentages calculate from NMR.

b Isolated yield.

It is worth noting that recycling experiments are obviously impacted by the loading of catalyst employed and the scale of the reaction—it is not appropriate to simply count how many times the reaction can be performed, but rather to determine the total capacity of the catalyst before it becomes ineffective. In the case of these agarose gel beads, we were using 0.05 mol % catalyst and achieved 2.5 uses of the catalyst on a 0.6 mmol scale reaction. In our previous study using agarose gel blocks,^50^ the catalyst loading was 20 times higher at 1.0 mol % and we achieved 13.74 uses of the catalyst on a 0.8 mmol scale reaction. To allow meaningful comparison between these recyclability studies, we calculated overall turnover numbers (TON_total_); this gave a TON_total_ of 5000 for the new agarose gel beads compared with a TON_total_ of 1375 for the agarose gel blocks previously reported. These numbers indicate that the agarose gel beads have greater inherent reusability than the agarose gel blocks, presumably because of the greater ease with which all catalytic sites can be accessed in the gel beads, and the greater mechanical integrity of the materials potentially helping stabilize the PdNPs. Given the nature of the naked PdNPs within these gels, without any stabilizing ligand being present, these observations are pleasing.

### Mechanistic Study of Gel Bead-Catalyzed Suzuki–Miyaura Cross-Coupling (SMCC) Reactions

Finally, we undertook mechanistic studies to understand the nature of our catalyst system. As noted above, evidence indicated that **DBS-CONHNH**_**2**_ was converted to **DBS-CO**_**2**_**H** during the *in situ* reduction of Pd(II) to Pd(0) and the resulting nanoparticles, with diameters *ca.* 3 nm, could therefore likely be considered as “naked” PdNPs. There has been considerable interest in catalysis using such “naked” ligand-free palladium nanoparticles.^65^ In general, smaller nanoparticles (<5 nm) such as those reported here are better as catalysts with catalysis probably occurring on the nanoparticle surface. However, such PdNPs generally do not catalyze coupling reactions of chloroarenes and can be prone to significant leaching unless stabilized by coordinating functional groups. However, from the earliest work, it was suggested that the absence of ligands could significantly enhance the catalytic activity of such surfaces.^69^ As such, a number of different approaches to stabilizing naked PdNPs have been taken, and used in Suzuki–Miyaura reactions.^70−73^ However, given the problems with naked PdNPs agglomerating and/or leaching, there can often be limits to the observed TONs that can be achieved using such systems.^74,75^ This is distinct from some of the ligand-stabilized Pd nanoparticles discussed in the introduction to this paper. As such, there are clear needs for more effective ways of stabilizing naked PdNPs, and we reason that our gel stabilization strategy reported here is one such way of doing so. Importantly, it has been shown that naked NPs, ligated NPs, and ligated Pd can give rise to different products in some reactions,^76^ and as such, there is a value in developing different types of supported Pd nanomaterials. There has been very considerable debate about the mechanism by which naked PdNPs achieve catalysis,^65,77^ with evidence being present both for heterogeneous and homogeneous leaching-type mechanisms—this may of course differ depending on the reaction conditions and nature of the catalyst support. For example, Fairlamb and co-workers previously reported that defect sites on nanoparticle surfaces played a key role in catalysis *via* a heterogeneous mechanism.^78,79^ We therefore wanted to explore the behavior of our new PdNP-loaded gels to understand the mechanism in this case as best as possible.

**Figure 5:**
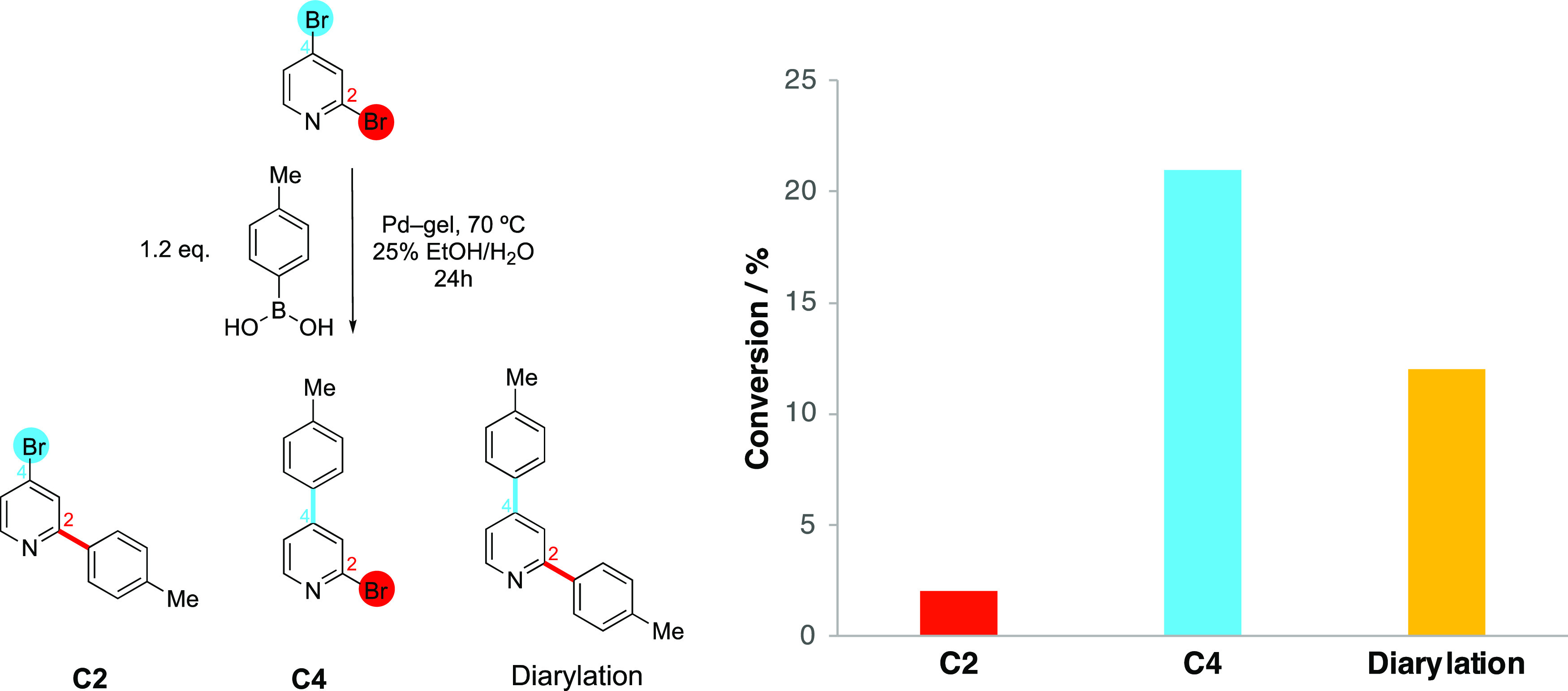
Regioselectivity test of SMCC reaction using 2,4-dibromopyridine with agarose/**DBS-CONHNH**_**2**_ gel beads as catalyst gives the C4-substituted compound as the major product, indicative of a catalytically active nonligated palladium source.

We first tested the regioselectivity of our gel system for the arylation of 2,4-dibromopyridine (Figure 5). Fairlamb and co-workers have previously demonstrated that nonligated PdNPs have a preference for reaction in the 4-position, contrary to more classical Pd catalysts with phosphine ligands where selectivity at the 2-position dominates.^80^ After 24 h at 70 °C with 1 equiv of 2,4-dibromopyridine and 1.2 equiv of **1**, the crude reaction mixture indicated 35% of the starting material was consumed. When analyzed by ^1^H NMR (Figure S36), this translated into 21% conversion into the C4 product, 12% into the diarylated product, and only 2% into the C2 product, demonstrating very good regioselectivity. Pleasingly, the beads did not show any sign of mechanical degradation even after 24 h at 70 °C. As such, we confirmed the nonligated nature of the PdNP catalyst in this system.

With the nature of the catalyst revealed, we were subsequently interested in determining whether catalysis occurred homogeneously or heterogeneously. There is already some evidence that the hybrid gel block composed of agarose and the LMWG acted predominantly as a heterogeneous catalyst. Relatively little leaching seemed to occur and when the reaction mixture from a completed reaction was isolated, then recharged with reagents, in the absence of the gel catalyst, the conversion only reached 34% in the time it normally takes for the reaction to reach quantitative conversions.^50^ However, for the alginate/**DBS-CONHNH**_**2**_ gel beads previously investigated, this conversion increased to 90%, indicative of very significant leaching problems and casting doubt onto the nature of the active catalyst.^52^

Pleasingly, when this test was performed on the new agarose/**DBS-CONHNH**_**2**_ gel bead system, it pointed clearly to a heterogeneous catalyst system. When the reaction mixture of the first recyclability run was separated from the gel catalyst and charged with 1 equiv of **a** and 1.2 equiv of **3**, only 6% conversion was reached, suggesting very little Pd leaching has taken place. Obviously, obtaining no conversion for this highly activated Suzuki–Miyaura cross-coupling would have been ideal, but given the challenges in stabilizing naked PdNPs,^74,75^ we were very pleased with this result as it was a significant improvement over our previous reports. We suggest that the shorter reaction time and the presence of the mechanically robust PG help minimize Pd leaching. The interwoven network of the agarose hybrid bead could potentially also play a role as a superior architecture for maintaining the integrity of the PdNPs. We also note that the **DBS-CO**_**2**_**H** formed upon the oxidation of **DBS-CONHNH**_**2**_ cannot gel as effectively in basic solutions (such as a Suzuki–Miyaura reaction mixture),^63^ hence the core–shell architecture of the alginate beads, with the core primarily composed of the LMWG, may cause the interior of the bead to be substantially damaged, leading to Pd leaching. Conversely, the interwoven nature of the agarose-based beads may better stabilize the PdNPs even if the **DBS-CO**_**2**_**H** network becomes somewhat damaged during the SMCC reaction. In future, it may be possible to eliminate the last traces of leaching using an unloaded bead as a Pd-capture agent in the reaction. Alternatively, the catalytic beads could deliberately be only part-loaded with Pd such that they have additional acylhydrazide groups present to re-reduce any Pd(II) that gets released from the naked PdNP surface. Experiments to test these concepts are ongoing in our laboratories.

To further confirm the heterogeneous nature of our catalyst system we carried out a poisoning experiment using Hg(0). Historically, it has been argued that Hg(0) inhibits reactions catalyzed by Pd colloids and nanoparticles; however, in recent years, the selectivity of this process has been disputed.^81,82^ The reaction between compounds **a** and **3** was studied. The kinetic profiles (Figure S37) clearly indicate that, within experimental error, there is no significant difference when Hg(0) is present; therefore, there is no inhibitory effect in this case. Further evidence for this is provided by TEM where there is no visual difference between the PdNPs after one run in the presence or absence of Hg(0) (Figure S35). We suggest that in this case, the results of the Hg test indicate that the catalytically active PdNP species is encapsulated and protected within the gel beads, and hence does not come into contact with the Hg(0)—as such, our PdNPs do not suffer from inhibition. It is also important to note that Hg(0) was not expected to perturb the proposed particular catalyst system, as noted by Ananikov and co-workers.^82^ This experiment also allowed us to use the kinetic plot for the reaction (Figure S37) to determine that the TON at conversion (60 min) was 1000, while the initial TOF (first 15 min of reaction) reached as high as 2000 h^–1^, indicative of pleasing performance from these gel-immobilized naked Pd nanoparticles.

Finally, a three-phase test further supported the hypothesis that catalysis occurs mostly heterogeneously (Figure S38). When using a resin-immobilized aryl iodide, only leached Pd can potentially catalyze the coupling by diffusing into the resin.^83,84^ After 4 h, the conversion was <5%, indicating that the leachate has minimal catalytic activity. The fact that using the gel beads for the solution phase reaction allows the isolation of compound **1e** after 4 h in very good yields serves as an excellent positive control, demonstrating that the catalytically active Pd is effectively gel-immobilized.

As such, we conclude that the PdNPs in these interwoven agarose-**DBS-CONHNH**_**2**_ gel beads can be considered as nonligated “naked” catalysts, that are firmly held within the gel phase network, and operate in a heterogeneous manner.

## Conclusions

In conclusion, self-assembling hybrid agarose/**DBS-CONHNH**_**2**_ (PG/LMWG) metallogel beads were synthesized and fully characterized to reveal an interwoven gel network benefitting from both components. The LMWG could remediate Pd(II) from aqueous solution, reducing Pd(II) to Pd(0), with the resulting PdNPs being stabilized inside the gel network—a process now mechanistically understood as being coupled with oxidation of the LMWG **DBS-CONHNH**_**2**_ in the aqueous hydrogel environment to give another LMWG, **DBS-CO**_**2**_**H**. The formation of interpenetrated PG/LMWG networks means agarose enhances the stiffness of the LMWG (and *vice versa*), facilitating easy handling and endowing these gel beads with enhanced mechanical robustness.

The beads were optimal catalysts for sustainable Suzuki–Miyaura cross-coupling (SMCC) reactions using aryl iodides, bromides, and even chlorides, with good functional group tolerance and easy gel bead recyclability. The reactions are trivial to purify, easy to scale up, and can give rise to pharmaceutically relevant building blocks.

Compared to the catalytically active gel block first reported by Slavik and co-workers,^50^ these new beads showed enhanced turnover frequencies. Furthermore, the superior robustness of the beads, in comparison to first-generation **DBS-CONHNH**_**2**_–alginate hybrid gel bead counterparts,^52^ allowed the introduction of stirring without any mechanical breakdown, significantly increasing the rate of reaction and enabling recyclability and reuse by simple filtration of the robust beads from the reaction mixture. Importantly, with a view to sustainability and efficiency, the new gel beads reported here suffer significantly less from Pd leaching than the previously reported systems.

Mechanistically, these systems behave as heterogeneous nonligated “naked” Pd catalysts with a pronounced preference for the nontypical C4-position^80^ in 2,4-dibromopyridine, little influence of Hg(0) on the reaction rate and very low conversion in a three-phase test. The shorter reaction times, higher PG loading, the interwoven architecture, and the choice of the more mechanically robust agarose over alginate all play a role.

In addition to the credentials of this system in terms of sustainable chemistry, we believe that catalysts (and reagents) encapsulated within gel delivery systems are an appealing format for further development as researchers increasingly look for ways to carry out chemical reactions in simple “kit form,” making complex reactions more appealing for use by nonspecialists, scientists working in other fields, or for application in automated systems. In this context, we can see very significant potential of these optimized PdNP-loaded agarose/**DBS-CONHNH**_**2**_ gel beads.

## Supporting Information Available

Full experimental methods, further characterization of gel beads, data from all assays, and characterization data for all organic compounds synthesized (PDF)
