# Supplementary material for: Mechanically Robust Hybrid Gel Beads Loaded with “Naked” Palladium Nanoparticles as Efficient, Reusable, and Sustainable Catalysts for the Suzuki–Miyaura Reaction
Source: ACS Sustain Chem Eng. 2023 Jan 24;11(5):1678–89. doi: 10.1021/acssuschemeng.2c05484 (PMC9906743; doi:10.1021/acssuschemeng.2c05484)
Supplement: Supplementary file 1 — sc2c05484_si_001.pdf [file sc2c05484_si_001.pdf]

SUPPORTING INFORMATION

Mechanically-Robust Hybrid Gel Beads Loaded with 'Naked' Palladium Nanoparticles as Efficient, Reusable, and Sustainable Catalysts for the Suzuki-Miyaura Reaction

Matteo Albino,[a] Thomas J. Burden,[a] Carmen C. Piras,[a] Adrian C. Whitwood,[a] Ian J. S. Fairlamb,[a] and David K. Smith[,a]*

Department of Chemistry, University of York, Heslington, York, YO10 5DD, UK

Number of Pages: 74; Number of Figures: 92; Number of Tables 16

S1.1 General Information

Solvents and Reagents: All reagents and solvents purchased from chemical suppliers including Fisher scientific, Fluorochem, Merck, Sigma-Aldrich and TCI, were used without any additional purification, except for 4'-iodoacetophenone (**a**) that was purified through flash-column chromatography. We thank Neda Jeddi from IJSF group (York) for providing aryl iodide resin used in the three-phase test.

Chromatography: Automated column chromatography was carried out using a Teledyne Combiflash Rf with standard Redisep® Rf 4 and 12 g disposable SiO_2 columns. Thin layer chromatography (TLC) plates used were Merck 5554 aluminium backed silica. Spots visualized with short (254 nm) and long wave (365 nm) UV.

NMR: All NMR spectra were recorded on a Jeol ECS400. Chemical shifts in ppm were calibrated from proton chemical shifts of the solvents. ^{1}H spectra were recorded at 400 MHz, ^{13}C spectra recorded at 101 MHz and ^{19}F recorded at 376 MHz. Coupling constants are reported in Hz. All spectra were processed using MestReNova. All spectra were reported at room temperature.

Mass Spectrometry: Electrospray ionisation (ESI) and (APCI) mass spectrometry was performed using a Bruker Daltronics micrOTOF spectrometer. GC-Electron impact (EI) Waters GCT Premier mass spectrometer. Mass to charge ratios (*m/z*) are reported in atomic mass units (Daltons).

Infrared Spectroscopy (University of York): Solid state spectra were recorded on a Perkin Elmer UATR Two FT-IR spectrometer. Absorption maxima (ν_{max}) are reported in wavenumbers (cm^{-1}) to the nearest whole number.

T_{gel} values: T_{gel} values were recorded using Huber Ministat cc2. Sample preparation and data measurement are described below.

Rheology: All rheological measurements were recorded on a Kinexus machine. Sample preparation and parameters are described below.

SEM and TEM: For TEM the gel beads were squashed between two glass slides, and then affixed to HT-treated formvar/carbon coated 200-mesh copper grids. Washed with drops of distilled water and left to dry for 30-60 minutes. Grids were then imaged with FEI Tecnai 12 Biotwin G2 transmission electron microscope operating at 120kV. Appropriate images were collected with SIS CCD camera. For SEM the samples were either were obtained by freeze drying the gels on copper shim pieces, and were then left untreated or sliced in order to expose the interior of the gel.

UV-Vis: All spectra were recorded on a Shimadzu UV-2401 PC with using 1 cm cuvettes. Initially, before each cycle of measurements two cuvettes containing pure deionized water were used as blank.

X-Ray Crystallography: Diffraction data were collected at 110 K using an Oxford Diffraction SuperNova diffractometer with Cu-Kα radiation (λ = 1.54184 Å) using an EOS CCD camera. The crystal was cooled with an Oxford Instruments Cryojet. Diffractometer control, data collection, initial unit cell determination, frame integration and unit-cell refinement were carried out with “Crysalis”.[1] Face-indexed absorption corrections were applied using spherical harmonics, implemented in SCALE3 ABSPACK scaling algorithm. OLEX2 was used for overall structure solution and refinement. Within OLEX2, the algorithm used for structure solution was “ShelXT dual-space”.[2] Refinement was carried out by full- matrix least-squares used the SHELXL-97[3] algorithm within OLEX2.[4] All non-hydrogen atoms were refined anisotropically. Crystalmaker® software was used to visualise the structures as well as generating the figures presented herein.

S1.2 Synthesis of Gelators

DBS-CO_2Me, DBS-CONHNH_2 and DBS-CONHNH_2 were all synthesised according to previously published methods and characterisation was in agreement with previous reports.[5,6]

S1.3 Gelation Procedures and Bead Production

General gelation procedures are listed below for producing gels in vials or gel beads, as the procedures are independent of the gel type. Section 1.3.3. then lists the proportion for producing **DBS-CONHNH$_2$**, agarose and hybrid gels.

S1.3.1 Gelation in Vials

The gelator was added in deionised water inside a vial. The vial was then heated with a heat-gun until full dissolution was achieved. The solution was allowed to cool down to room temperature forming the gel.

S1.3.2 Gel Bead Production

Bead production was performed using the literature procedure previously reported by our group.[7] The gelator was suspended in deionised water inside a vial. The vial was then heated with a heat-gun until full dissolution was achieved. Using a pipette, the hot solution was dropped drop-wise into ice-cold paraffin oil with the volume of the drop determining the final size of the bead. The beads were then left for 30 minutes in the paraffin oil to ensure complete formation of the gel network, washed in 40–60 ºC petrol ether (3 x 10 mL), ethanol (3 x 10 mL) and deionised water (3 x 10 mL), by leaving the beads in each solution for 10 minutes.

S1.3.3 Proportions for the Production of Gel Beads or Gels in Vials

The proportions to produce gels in vials or in beads are the same and are reported in Table S1.

Table S1 Mass of **DBS-CONHNH$_2$**, agarose and volume of water ratio for the production of gel in vials or gel beads.

	**DBS-CONHNH$_2$ / mg**	**Agarose / mg**	**H_2O / mL**
**DBS-CONHNH$_2$**	3	0	1
**Agarose**	0	13	1
**Hybrid**	3	10	1

S1.4 NMR Studies

S1.4.1 Proof of Gelation

Ten 5 µL hybrid beads were placed in an NMR tube with D_2O and a ^1H NMR recorded.

S1.4.2 Thermal Stability

Ten 20 µL beads placed in an NMR tube with D_2O and 1.4 µL of DMSO-H_6 as internal standard. A ^1H NMR spectrum was recorded at t = 0 min and room temperature. The sample was then heated to 90 ºC and a spectrum recorded every after 30 minutes, followed by one every 15 minutes for the first hour, and then at t = 1.5, 2.5, 4.5, 8.5 and 13.5 hours. The integration of the aromatic peaks (δ 7.56 ppm) with respect to the integration of the DMSO signal (δ 2.50 ppm) was used to find the mass of **DBS-CONHNH$_2$** that leached over time.

S1.4.3 Quantification of **DBS-CONHNH$_2$** Content in Hybrid Beads

Ten 5 µL beads were left to dry overnight under high vacuum to constant mass. They were then dissolved in DMSO-d_6 together with 1.4 µL of acetonitrile-H_3 as an internal standard. A ^1H NMR spectrum was then recorded. The integration of the aromatic peaks with respect to the integration of the acetonitrile signal was used to find the mass of **DBS-CONHNH$_2$** present in the sample.

S1.5 Metal Uptake and UV-Vis Studies

40 5 µL hybrid beads, 40 5 µL agarose beads and 1 mL of **DBS-CONHNH$_2$** were added to a vial together with 3 mL of a 5 mM [Pd^{II}] aqueous solution. absorbance of the solution was monitored over 72 hours. To assure complete dissolution, the $PdCl_2$ solution was slightly acidified by addition of a few drops of 4 M HCl. The procedure was repeated three times per material type to ensure reliability.

S1.6 Investigation into **DBS-CONHNH$_2$** Oxidation

S1.6.1 Oxidised Xerogel Dissolution into DMSO-d_6

Three separate 1 mL hybrid gel blocks in vials were exposed to 3 mL of slightly acidified 5 mM $PdCl_2$ solution. After 24 hours, the gel Pd solution was removed and the gel left to dry to

constant mass. The xerogel was then dissolved in DMSO-d_6 and filtered through a plug of Celite® twice. The NMR samples were then run normally. Successively, the sample was split into two new samples, and each one was spiked with *ca.* 5mg of **DBS-CONHNH$_2$** and **DBS-CO$_2$H**, respectively. A ^{1}H NMR spectrum was then run again as normal for each sample.

S1.6.2 NMR of Water-Soluble Products

1 mL of the hybrid system was exposed to 3 mL of slightly acidified aqueous 5 mM $PdCl_2$ solution. After 24 hours, the gel Pd solution was removed and the gel left to dry to constant mass. The xerogel was added to a 0.1 M NaOD solution in D_2O and sonicated for 15 minutes. The solution was filtered through a plug of Celite® and a ^{1}H NMR spectrum recorded. For comparison, 3 mg of **DBS-CO$_2$H** were dissolved in 0.1 M NaOD solution in D_2O and a ^{1}H NMR spectrum recorded.

S1.7 IR Sample Preparation

Hybrid and agarose beads were prepared following the procedure described in section 7.3.2. The **DBS-CONHNH$_2$** was prepared in vials as described in section 7.3.1. Ten hybrid beads, ten agarose beads and 1 mL of **DBS-CONHNH$_2$** were immersed in an aqueous metal ion solution concentrated enough to allow maximum uptake. These three samples together with ten hybrid beads, ten agarose beads and 1 mL of **DBS-CONHNH$_2$** without any metal were left to dry under high vacuum to constant mass. The powders were then used to record IR spectra for each system.

S1.8 Measurement of T_{gel} Values

Samples were prepared in vials and immersed in an oil bath. The temperature of the oil bath was increased regularly from room temperature to 100 ºC in 30 minutes. When the structure began collapsing (the gel could not support its weight), T_{gel} was recorded. This was performed in triplicate, and an average taken.

S1.9 Rheology Sample Preparation and Investigation Parameters

Samples were prepared in bottomless vials and then transferred to the instrument. For samples containing metals, the samples were left for 24 hours to ensure full incorporation of the former

in the gel network. The measurements were carried out at room temperature using a 20 mm parallel plate and a gap of 2 mm. To avoid solvent evaporation and keep the sample hydrated, a solvent trap was used, and the internal atmosphere was kept saturated. For the amplitude sweep, the shear strain ranged from 0.05% to 100% with a constant frequency of 1 Hz. The frequency in the frequency sweep ranged from 100 to 0.1 Hz at a constant shear strain of 0.05%. The temperature was kept constant at 25 ºC for both measurements.

S1.10 General Procedure for Suzuki-Miyaura Cross Couplings

Arylhalide (0.60 mmol, 1.0 eq.), boronic acid (0.72 mmol, 1.2 eq.) and K_2CO_3 (167.40 mg, 1.20 mmol, 2.0 eq.) were added to a round-bottom flask equipped with a stirring bar, containing 15 mL of 25% aqueous EtOH solution which produced a suspension. The temperature was raised to 50 ºC for aryl iodides or 70 ºC for aryl bromides and the solution was then stirred vigorously for a period of 5 minutes, after which the stirring was turned to a gentle rate. 6 x 5 µL Pd-loaded hybrid gel beads (equivalent to *ca.* 0.05 mol% Pd) for aryl iodides, or 12 x 5 µL Pd-loaded hybrid gel beads (equivalent to *ca*. 0.10 mol% Pd) for aryl bromides were added to the reaction mixture. Reaction progress was monitored *via* ^{1}H NMR analysis by monitoring product appearance and starting material consumption. Upon completion, the product was extracted with 30 mL of CH_2Cl_2 from both the solution mixture and beads. The combined organic solutions were washed with 1 M NaOH solution. The organic phase was separated, dried over $MgSO_4$, and after filtration, the solvent was removed *in vacuo* to yield the expected product which was dried to constant mass.

S1.10.1 Gram-Scale Synthesis of **1a**

The procedure for the gram-scale synthesis of **1a** was scaled-up from the procedure in section 7.11 by using 1.50 g of aryl iodide (6.10 mmol, 1.0 eq.), 1.00 grams of boronic acid (7.32 mmol, 1.2 eq.), 1.69 g of K_2CO_3 (12.20 mmol, 2 eq.), 45 x 5 µL Pd-loaded hybrid gel beads (*ca*. 0.03 mol % [Pd]) and 152 mL of a 25% aqueous EtOH solution. The product was isolated in 97% yield after 6 hours.

S1.11 Control Studies and Pd-Speciation Investigation.

S1.11.1 Standard Reaction with No Stirring

The standard reaction between 4'-iodoacetophenone, **a**, (147.6 mg, 0.60 mmol, 1.0 eq.) and 4-tolylboronic acid, **1**, (97.9 mg, 0.72 mmol, 1.2 eq.) was performed exactly as described in section 1.11 with the exception of the stirring being turned completely off before addition of the beads. The conversion after an hour was determined *via* ^{1}H NMR.

S1.11.2 Standard Reaction Using No Pd-Loaded Beads

The standard reaction between 4'-iodoacetophenone, **a**, (147.6 mg, 0.60 mmol, 1.0 eq.) and 4-tolylboronic acid, **1**, (97.9 mg, 0.72 mmol, 1.2 eq.) was performed exactly as described in section 1.11 with the exception of no addition of Pd-loaded beads. The conversion after an hour was determined *via* ^{1}H NMR.

S1.11.3 Standard Reaction Using Metal Free-Gel Beads

The standard reaction between 4'-iodoacetophenone, **a**, (147.6 mg, 0.60 mmol, 1.0 eq.) and 4-tolylboronic acid, **1**, (97.9 mg, 0.72 mmol, 1.2 eq.) was performed exactly as described in section 1.11 with the exception of addition of Pd-free beads instead of the metallogel beads. The conversion after an hour was determined *via* ^{1}H NMR.

S1.11.4 Reaction using Agarose–only Beads

The standard reaction between 4'-iodoacetophenone, **a**, (147.6 mg, 0.60 mmol, 1.0 eq.) and 4-tolylboronic acid, **1**, (97.9 mg, 0.72 mmol, 1.2 eq.) acid was performed exactly as described in section 1.11, with the exception of six Pd–loaded agarose beads being added instead of 6 hybrid beads. Conversion was monitored *via* ^{1}H NMR.

S1.11.5 Recyclability Studies

The standard reaction between 4'-iodoacetophenone, **a**, (147.6 mg, 0.60 mmol, 1.0 eq.) and 4-tolylboronic acid, **1**, (97.9 mg, 0.72 mmol, 1.2 eq.) acid was performed exactly as described in section 1.11. After washing, the beads were placed in a new reaction mixture. Conversion was monitored *via* ^{1}H NMR.

S1.11.6 Mercury Poisoning

The control reaction between 4'-iodoacetophenone, **a**, (147.6, 0.60 mmol, 1.0 eq.) and 4-methoxyphenylboronic acid, **3**, (109.4 mg, 0.72 mmol, 1.2 eq.) was carried out as described in section 1.11, with the catalyst loading doubled to twelve 5 µL Pd-loaded hybrid gel beads (0.10 mol % [Pd]) in order to allow more facile monitoring of the reaction progress. This was achieved *via* ^{1}H NMR analysis of samples taken from the reaction in different time intervals by monitoring the acetyl CH_3 protons in the starting material and products. The aforementioned reaction was then repeated (with higher rate of stirring) with the addition of metallic mercury (2.6 µL, 0.18 mmol, 0.3 eq) at 15 minutes after starting time, when the reaction had appeared to reach *ca.* 50% conversion to the expected product as determined by ^{1}H NMR of the control experiment. The Hg^0 was added *via* syringe to the stirring reaction and aliquots at time intervals were taken again for ^{1}H NMR analysis.

S1.11.7 Three-Phase System Suzuki-Miyaura Cross Couplings

4-Tolylboronic acid (40.8 mg, 0.30 mmol, 1.0 eq.), aminomethyl polystyrene-immobilised 4-iodobenzamide (199.5 mg, 0.30 mmol, 1.0 eq.), K_2CO_3 (83.7 mg, 0.60 mmol, 2.0 eq.) and 1,3,5-trimethoxybenzene (25.2 mg, 0.15 mmol, 0.5 eq. internal standard) were added to a round-bottom flask equipped with a stirring bar, containing 7.5 mL of 25% aqueous EtOH solution. The mixture was heated to 50 ºC and stirred vigorously for 5 minutes. The stirring was then turned down to gentle, and 3 x 5 µL Pd-loaded hybrid gel beads (ca. 0.05 mol % [Pd]) were added to the mixture. Aliquots taken at 1 hour and 4 hours from the reaction media were analysed by ^{1}H NMR to determine the conversion of 4-tolylboronic acid (in respect to internal standard) to the expected immobilised product. To ensure that no boronic acid degradation occurred under these conditions, a reaction mixture containing the same reagents, but no beads, lead to no decrease in boronic acid integral with respect to the internal standard.

S1.11.8 Leaching Experiment

The reaction between 4'-iodoacetophenone and 4-tolylboronic acid was allowed to react for 3 hours using the same procedure as described in section 1.11. The product was then extracted and the aqueous layer was made up to 15 mL by the addition of a 25% aqueous EtOH solution. 4'-iodoacetophenone, **a**, (0.60 mmol) and 4-methoxyphenylboronic (0.72 mmol) acid, **3**, were added and the reaction mixture heated at 50 ºC for 3 hours. The reaction mixture was then extracted using CH_2Cl_2, dried over $MgSO_4$ and ^{1}H NMR was used to determine the conversion.

S2 Suzuki-Miyaura Product Characterisation

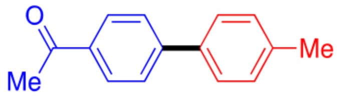

1a: Light yellow solid; Yield: 90%; FTIR (ATR) / cm^{-1}: 2962, 1678, 1599, 1495, 1361, 1260, 1181, 1081, 961, 847, 805, 752; 1H NMR (400 MHz, Chloroform-*d*) δ 8.05 – 7.98 (m, 2H), 7.71 – 7.64 (m, 2H), 7.58 – 7.50 (m, 2H), 7.33 – 7.27 (m, 2H), 2.64 (s, 3H), 2.41 (s, 3H); ^{13}C NMR (101 MHz, Chloroform-*d*): δ 198.0, 145.9, 138.4, 137.1, 135.7, 129.8, 129.1, 127.2, 127.1, 26.8, 21.3; GC-EI (m/z) calculated for $C_{15}H_{14}O$ 210.1045; found 210.1030 (M^+). Characterisation data is consistent with literature.[8] Crystals were grown from slow evaporation of hexane.

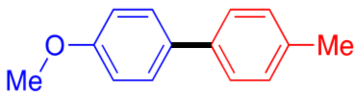

1b: colourless powder; Yield: 91%; FTIR (ATR) / cm^{-1}: 2914, 1606, 1582, 1531, 1499, 1440, 1374, 1326, 1287, 1250, 1218, 1182, 1138, 1115, 1037, 1013, 1000, 842, 805, 783, 723; ^{1}H NMR (400 MHz, Chloroform-*d*) δ 7.52 (d, *J* = 8.4 Hz, 2H), 7.46 (d, *J* = 7.8 Hz, 2H), 7.24 (d, *J* = 7.7 Hz, 2H), 6.98 (d, *J* = 8.2 Hz, 2H), 3.85 (s, 3H), 2.39 (s, 3H); ^{13}C NMR (101 MHz, Chloroform-*d*) δ 159.1, 138.1, 136.5, 133.9, 129.6, 128.1, 114.3, 55.5, 21.2; ESI-MS (m/z) calculated for $C_{14}H_{15}O$ 199.1123; found 199.1117 ($[M+H]^+$). Characterisation data is consistent with literature.[8]

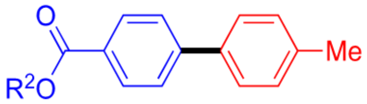

1c[1]: colourless powder; Yield: R = Me 36%, R = Et 12%, (48% total). The two compounds were not isolated; FTIR (ATR) / cm^{-1}: 2947, 1705, 1603, 1532, 1495, 1432, 1399, 1367, 1331, 1284, 1271, 1223, 1199, 1178, 1110, 1018, 1003, 965, 861, 818, 804, 766, 698; R = Me, ^{1}H NMR (400 MHz, Chloroform-*d*) δ 8.14 – 8.04 (m, 2H), 7.70 – 7.60 (m, 2H), 7.58 – 7.50 (m, 2H), 7.31 – 7.27 (m, 2H), 3.94 (s, 3H), 2.40 (s, 3H); R = Et, ^{1}H NMR (400 MHz, Chloroform-*d*) δ 8.14 – 8.04 (m, 2H), 7.70 – 7.60 (m, 2H), 7.58 – 7.50 (m, 2H), 7.31 – 7.27 (m, 2H), 4.40 (q, *J* = 7.1 Hz, 2H), 2.40 (s, 3H), 1.42 (t, *J* = 7.1 Hz, 3H); R = Me, ^{13}C NMR (101 MHz, Chloroform-*d*) δ 167.2, 145.7, 138.3, 137.3, 130.2, 129.8, 128.7, 127.3, 126.9, 52.2, 21.3; R = Et ^{13}C NMR (101 MHz, Chloroform-*d*) δ 166.7, 145.6, 138.2, 137.2, 130.2, 129.6, 129.1,

127.3, 126.9, 61.1, 21.2, 14.5; R = Me, ESI-MS (m/z) calculated for $C_{15}H_{15}O_2$ 227.1072 and for $C_{15}H_{14}O_2Na$ 249.0891; found 227.1067 ($[M+H]^+$), 249.0881 ($[M+Na]^+$); R = Et, ESI-MS (m/z) calculated for $C_{16}H_{17}O_2$ 241.1229 and for $C_{16}H_{16}O_2Na$ 263.1048; found 241.1215 ($[M+H]^+$), 263.1045 ($[M+Na]^+$). Characterisation data is consistent with literature for R = Me,[9] and for R = Et.[10]

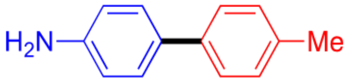

1d: brown powder; Yield: 81%; FTIR (ATR) / cm^{-1}: 3423, 3383, 3291, 3189, 2917, 1624, 1604, 1529, 1500, 1270, 1178, 1136, 1039, 1003, 948, 854, 840, 804, 749, 723, 710, 661; ^{1}H NMR (400 MHz, Chloroform-*d*) δ 7.54 – 7.32 (m, 4H), 7.22 (d, *J* = 7.9 Hz, 2H), 6.80 – 6.70 (m, 2H), 2.38 (s, 3H); ^{13}C NMR (101 MHz, Chloroform-*d*) δ 145.7, 138.4, 136.0, 131.7, 129.5, 128.0, 126.4, 115.5, 21.2; ESI-MS (m/z) calculated for $C_{13}H_{14}N$ 184.1126; found 184.1128 ($[M+H]^+$). Crystals were grown from a saturated solution by slow evaporation of cyclohexane. Characterisation data is consistent with literature.[11]

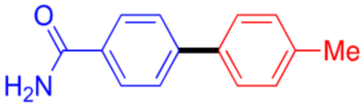

1e: product extracted with diethyl ether; white powder; Yield: 81%; FTIR (ATR) / cm^{-1}: 3400, 3159, 2918, 2855, 1644, 1614, 1577, 1556, 1421, 1400, 1377, 856, 815, 796, 776, 761; ^{1}H NMR (400 MHz, DMSO-d_6) δ 8.01 (s, 1H), 7.98 – 7.92 (m, 2H), 7.75 – 7.70 (m, 2H), 7.64 – 7.60 (m, 2H), 7.38 (s, 1H), 7.31 – 7.26 (m, 2H), 2.35 (s, 3H). ^{13}C NMR (101 MHz, DMSO-d_6) δ 167.6, 142.7, 137.5, 136.3, 132.8, 129.6, 128.2, 128.1, 126.7, 126.1, 20.7. ESI-MS (m/z) calculated for $C_{14}H_{14}NO$ 212.1075 and for $C_{14}H_{13}NONa$ 234.0895; found 212.1067 ($[M+H]^+$), 234.0894 ($[M+Na]^+$). Crystals were grown from slow evaporation of a saturated warm MeCN solution.

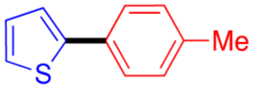

1f: colourless powder; Yield: 97%; FTIR (ATR) / cm^{-1}: 2912, 1729, 1533, 1500, 1431, 1375, 1313, 1259, 1211, 1124, 1052, 1018, 957, 850, 807, 684, 636; ^{1}H NMR (400 MHz, Chloroform-*d*) δ 7.52 (d, *J* = 8.2 Hz, 2H), 7.28 (dd, *J* = 3.6, 1.1 Hz, 1H), 7.26 – 7.24 (m, 1H), 7.20 (d, *J* = 7.8 Hz, 2H), 7.08 (dd, *J* = 5.1, 3.5 Hz, 1H), 2.38 (s, 3H); ^{13}C NMR (101 MHz,

Chloroform-*d*) δ 144.7, 137.5, 131.8, 129.7, 128.1, 126.0, 124.4, 122.7, 21.3. ESI-MS (m/z) calculated for $C_{11}H_{11}S$ 175.0581; found 175.0565 ($[M+H]^+$). Characterisation data is consistent with literature.[11]

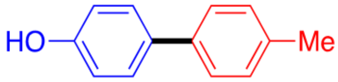

1g: note: both the reaction mixture and the NaOH solution were slightly acidified by addition of a few drops of concentrated HCl as the deprotonated product was water soluble. Colourless powder; Yield: 95%; FTIR (ATR) / cm^{-1}: 3326, 3031, 2913, 2856, 1609, 1598, 1580, 1498, 1447, 1372, 1317, 1298, 1261, 1217, 1195, 1178, 1135, 1111, 1022, 1003, 840, 806, 733, 683, 665, 494; ^{1}H NMR (400 MHz, Chloroform-*d*) δ 7.46 (t, *J* = 8.6 Hz, 4H), 7.24 (d, *J* = 7.9 Hz, 2H), 6.90 (d, *J* = 8.1 Hz, 2H), 4.96 (s, 1H), 2.40 (s, 3H). ^{13}C NMR (101 MHz, Chloroform-*d*) δ 155.0, 138.0, 136.6, 134.1, 129.6, 128.3, 126.7, 115.7, 21.2. ESI-MS (m/z) calculated for $C_{13}H_{13}O$ 185.0966; found 183.0815 ($[M+H]^+$). Characterisation data is consistent with literature.[12]

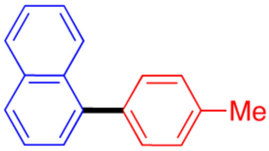

1h: colourless dense oil; Yield: 88%; FTIR (thin film) / cm^{-1}: 3044, 2918, 1908, 1641, 1515, 1505, 1445, 1184, 1110, 1021, 1903, 821, 798, 775, 749, 721, 692, 672, 569, 553, 500; ^{1}H NMR (400 MHz, Chloroform-*d*) δ 7.92 (ddt, *J* = 8.7, 8.0, 1.0 Hz, 2H), 7.85 (dd, *J* = 8.2, 1.1 Hz, 1H), 7.58 – 7.36 (m, 6H), 7.36 – 7.29 (m, 2H), 2.47 (s, 3H). ^{13}C NMR (101 MHz, Chloroform-*d*) δ 140.4, 138.0, 137.1, 134.0, 132.0, 130.1, 129.1, 128.4, 127.6, 127.0, 126.3, 126.0, 125.9, 125.5, 21.4. APCI-MS (m/z) calculated for $C_{17}H_{15}$ 219.1174; found 219.1158 ($[M+H]^+$). Characterisation data is consistent with literature.[13]

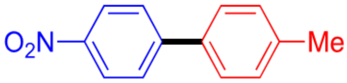

1i: orange-brown powder; Yield: 87%; FTIR (ATR) / cm^{-1}: 2922, 1594, 1510, 1484, 1338, 1191, 1108, 1005, 855, 822, 753, 716, 696; ^{1}H NMR (400 MHz, Chloroform-*d*) δ 8.45 – 8.15 (m, 2H), 7.85 – 7.65 (m, 2H), 7.63 – 7.44 (m, 2H), 7.35 – 7.28 (m, 2H), 2.43 (s, 3H); ^{13}C NMR (101 MHz, Chloroform-*d*) δ 147.7, 147.0, 139.2, 136.0, 130.0, 127.6, 127.4, 124.2, 21.3; ESI-

MS (m/z) calculated for $C_{13}H_{11}NO_2Na$: 236.0682; found 236.0691 ($[M+Na]^+$). Characterisation data is consistent with literature.[11]

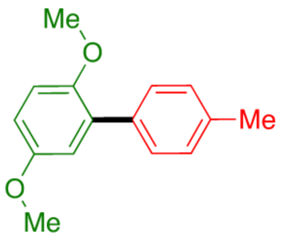

1j: colourless oil; Yield: 97%; FTIR (thin film) / cm^{-1}: 2941, 2832, 1721, 1608, 1518, 1495, 1463, 1397, 1294, 1259, 1216, 1177, 1112, 1050, 1026, 881, 821, 803, 735, 608; ^{1}H NMR (400 MHz, Chloroform-*d*) δ 7.49 – 7.42 (m, 2H), 7.25 – 7.22 (m, 2H), 6.94 – 6.89 (m, 2H), 6.84 (dd, *J* = 8.7, 3.2 Hz, 1H), 3.81 (s, 3H), 3.75 (s, 3H), 2.40 (s, 3H); ^{13}C NMR (101 MHz, Chloroform-*d*) δ 153.9, 151.0, 137.0, 135.6, 131.8, 129.6, 129.4, 128.9, 127.0, 116.8, 113.0, 112.7, 56.4, 55.9, 21.4. ESI-MS (m/z) calculated for $C_{15}H_{17}O_2$ 229.1229 and for $C_{15}H_{16}O_2Na$ 251.1048; found 229.1225 ($[M+H]^+$), 251.1046 ($[M+Na]^+$).

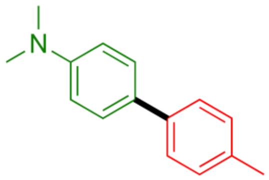

1k: colourless powder; Yield: 99%; FTIR (ATR) / cm^{-1}: 2917, 1606, 1528, 1502, 1441, 1351, 1218, 1197, 1158, 1114, 1061, 1016, 946, 834, 805, 731, 634; ^{1}H NMR (400 MHz, Chloroform-*d*) δ 7.53 – 7.49 (m, 2H), 7.49 – 7.44 (m, 2H), 7.25 – 7.19 (m, 2H), 6.85 – 6.78 (m, 2H), 3.00 (s, 6H), 2.39 (s, 3H). ^{13}C NMR (101 MHz, Chloroform-*d*) δ 150.0, 138.5, 135.8, 129.6, 129.5, 127.7, 127.0, 126.3, 113.0, 40.8, 21.2. ESI-MS (m/z) calculated for $C_{15}H_{18}N$ 212.1439; found 212.1435 ($[M+H]^+$). Characterisation data is consistent with literature.[14]

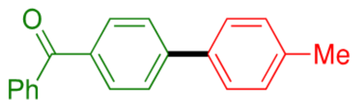

1l: colourless powder; Yield: 76%; FTIR (ATR) / cm^{-1}: 3022, 2912, 1643, 1596, 1578, 1444, 1314, 1288, 1273, 1225, 1201, 1150, 939, 923, 818, 789, 748, 732, 690, 666, 598, 460; ^{1}H NMR (400 MHz, Chloroform-*d*) δ 7.93 – 7.87 (m, 2H), 7.87 – 7.81 (m, 2H), 7.75 – 7.65 (m, 2H), 7.65 – 7.58 (m, 1H), 7.58 – 7.54 (m, 2H), 7.54 – 7.46 (m, 2H), 7.29 (d, *J* = 7.9 Hz, 2H), 2.42 (s, 3H). ^{13}C NMR (101 MHz, Chloroform-*d*) δ 196.5, 145.4, 138.3, 138.0, 137.2, 136.1,

132.5, 130.9, 130.1, 129.9, 128.4, 127.3, 126.9, 21.3. ESI-MS (m/z) calculated for $C_{20}H_{17}O$ 273.1279 and for $C_{15}H_{16}O_2Na$ 295.1099; found 273.1276 ($[M+H]^+$), 295.1097 ($[M+Na]^+$). Characterisation data is consistent with literature.[15]

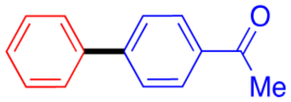

2a: yellow powder; Yield: 92%; FTIR (ATR) / cm^{-1}: 3343, 3075, 3000, 2921, 2859, 1995, 1676, 1601, 1583, 1559, 1516, 1451, 1442, 1424, 1403, 1357, 1311, 1283, 1261, 1208, 1178, 1120, 1078, 1039, 1020, 1005, 916, 860, 834, 763, 721, 678, 689, 591, 536, 495. ^{1}H NMR (400 MHz, Chloroform-*d*) δ 8.07 – 8.01 (m, 2H), 7.74 – 7.64 (m, 2H), 7.65 – 7.62 (m, 2H), 7.52 – 7.45 (m, 2H), 7.44 – 7.38 (m, 1H), 2.64 (s, 3H). ^{13}C NMR (101 MHz, Chloroform-*d*) δ 197.9, 145.9, 140.0, 136.0, 129.1, 129.04, 128.4, 127.4, 127.4, 26.8**.** ESI-MS (m/z) calculated for $C_{20}H_{17}O$ 197.0966 and for $C_{15}H_{16}O_2Na$ 219.0786; found 197.0967 ($[M+H]^+$), 219.0786 ($[M+Na]^+$). Characterisation data is consistent with literature.[16]

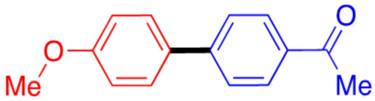

3a: light yellow powder; Yield: 96%; FTIR (ATR) / cm^{-1}: 2959, 1728, 1673, 1598, 1580, 1527, 1495, 1462, 1426, 1400, 1360, 1312, 1289, 1253, 1199, 1138, 1032, 1011, 999, 959, 815, 713, 639, 591; ^{1}H NMR (400 MHz, Chloroform-*d*) δ 8.09 – 7.97 (m, 2H), 7.68 – 7.61 (m, 2H), 7.60 – 7.54 (m, 2H), 7.04 – 6.94 (m, 2H), 3.87 (s, 3H), 2.63 (s, 3H). ^{13}C NMR (101 MHz, Chloroform-*d*) δ 197.9, 160.1, 145.5, 135.4, 132.4, 129.1, 128.5, 126.8, 114.5, 55.5, 26.8. GC-EI (m/z) calculated for $C_{15}H_{14}O_2$ 226.0994; found 226.0981 (M^+). Characterisation data is consistent with literature.[17]

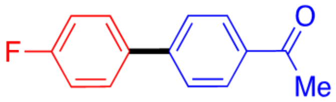

4a: yellow powder; Yield: 90%; FTIR (ATR) / cm^{-1}: 2981, 1680, 1599, 1528, 1495, 1422, 1396, 1360, 1324, 1279, 1253, 1195, 1163, 1015, 1004, 961, 817, 707, 639, 589; ^{11}H NMR (400 MHz, Chloroform-*d*) δ 8.08 – 7.96 (m, 2H), 7.68 – 7.62 (m, 2H), 7.62 – 7.56 (m, 2H), 7.20 – 7.12 (m, 2H), 2.64 (s, 3H); ^{19}F NMR (376 MHz, Chloroform-*d*) δ -113.83 – -113.96 (m). ^{13}C NMR (101 MHz, Chloroform-*d*) δ 197.9, 163.1 (d, *J* = 248.1 Hz), 144.9, 136.1 (d, *J* = 3.4 Hz), 136.0, 129.1, 129.0, 127.2, 116.1 (d, *J* = 21.6 Hz), 26.8; ESI-MS (m/z) calculated

for $C_{15}H_{12}OF$ 215.0872 and for $C_{15}H_{11}OFNa$ 237.0692; found 215.0864 ($[M+H]^+$), 237.0682 ($[M+Na]^+$). Characterisation data is consistent with literature.[18]

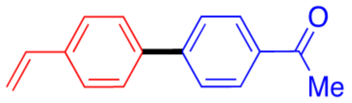

5a: yellow powder; Yield: 80%; FTIR (ATR) / cm^{-1}: 3100, 2919, 1821, 3346, 3002, 1678, 1627, 1579, 1551, 1526, 1494, 1415, 1397, 1360, 1330, 1265, 1221, 1185, 1140, 1082, 1016, 1003, 991, 960, 906,m 822, 748, 723, 638, 591, 579, 527. ^{1}H NMR (400 MHz, Chloroform-*d*) δ 8.25 – 7.90 (m, 2H), 7.73 – 7.66 (m, 2H), 7.61 (d, *J* = 8.1 Hz, 2H), 7.55 – 7.49 (m, 2H), 6.77 (dd, *J* = 17.6, 10.9 Hz, 1H), 5.82 (dt, *J* = 17.7, 0.8 Hz, 1H), 5.31 (dt, *J* = 10.9, 0.8 Hz, 1H), 2.64 (s, 3H). ^{13}C NMR (101 MHz, Chloroform-*d*) δ 197.9, 145.4, 139.3, 137.7, 136.3, 136.0, 129.1, 127.5, 127.1, 127.0, 114.7, 26.8. ESI-MS (m/z) calculated for $C_{16}H_{15}O$ 223.1123 and for $C_{15}H_{16}O_2Na$ 245.0942; found 223.1120 ($[M+H]^+$), 245.0941 ($[M+Na]^+$). Characterisation data is consistent with literature.[19]

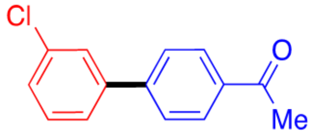

6a: light yellow solid; Yield: 93%; FTIR (ATR) / cm^{-1}: 2923, 1684, 1603, 1574, 1553, 1470, 1421, 1393, 1353, 1209, 1291, 1262, 1183, 1165, 1098, 1076, 1034, 1010, 959, 874, 845, 832, 804, 785, 733, 725, 691, 663, 595; ^{1}H NMR (400 MHz, Chloroform-*d*) δ 8.04 (d, *J* = 8.4 Hz, 1H), 7.68 – 7.63 (m, 1H), 7.60 (t, *J* = 1.9 Hz, 1H), 7.50 (dt, *J* = 7.1, 1.8 Hz, 1H), 7.43 – 7.37 (m, 1H), 2.64 (s, 2H). ^{13}C NMR (101 MHz, Chloroform-*d*) δ 197.8, 144.4, 141.8, 136.5, 135.0, 130.3, 129.1, 128.4, 127.5, 127.4, 125.6, 26.8. ESI-MS (m/z) calculated for $C_{14}H_{11}ClONa$ 253.0396; found 253.0391 ($[M+Na]^+$). Characterisation data is consistent with literature.[20]

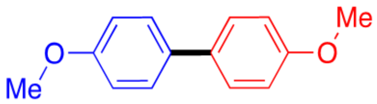

3b: pale yellow crystalline powder; Yield: 92%; FTIR (ATR) / cm^{-1}: 2959, 1605, 1570, 1498, 1438, 1329, 1274, 1249, 1138, 1040, 1012, 997, 823, 809, 781; ^{1}H NMR (400 MHz, Chloroform-*d*) δ 7.60 – 7.33 (m, 4H), 7.06 – 6.81 (m, 4H), 3.85 (s, 6H); ^{13}C NMR (101 MHz, Chloroform-*d*) δ 158.8, 133.6, 127.9, 114.3, 55.5; ESI-MS (m/z) calculated for $C_{14}H_{15}O_2$ 215.1072; found 215.1055 ($[M+H]^+$). Characterisation data is consistent with literature.[9]

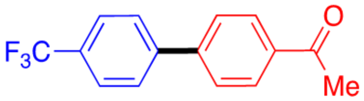

7m: pale yellow powder; Yield: 70%; FTIR (ATR) / cm^{-1}: 2981, 1683, 1605, 1559, 1420, 1396, 1354, 1320, 1251, 1158, 1111, 1068, 1021, 1004, 965, 863, 846, 822, 754, 725, 603, 592; ^{1}H NMR (400 MHz, Chloroform-*d*) δ 8.20 – 7.90 (m, 2H), 7.73 (s, 4H), 7.71 – 7.67 (m, 2H), 2.65 (s, 3H); ^{19}F NMR (376 MHz, Chloroform-*d*) δ -62.43. ^{13}C NMR (101 MHz, Chloroform-*d*) δ 197.8, 144.3, 143.5, 136.7, 130.4 (q, *J* = 32.9 Hz), 129.2, 127.8, 127.6, 126.1 (q, *J* = 2.9 Hz), 122.9, 26.9; ESI-MS (m/z) calculated for $C_{15}H_{11}OF_3Na$ 287.0660; found 287.0655 ([M+Na]$^+$). Characterisation data is consistent with literature.[21]

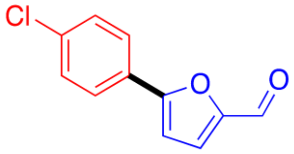

8n: orange powder; Yield: 87%; FTIR (ATR) / cm^{-1}: 2981, 1675, 1662, 1601, 1521, 1477, 1414, 1356, 1291, 1278, 1255, 1212, 1108, 1094, 1040, 968, 928, 832, 810, 792, 783, 768, 738, 669, 630, ; ^{1}H NMR (400 MHz, Chloroform-*d*) δ 9.65 (s, 1H), 7.85 – 7.64 (m, 2H), 7.48 – 7.35 (m, 2H), 7.32 (d, *J* = 3.7 Hz, 1H), 6.83 (d, *J* = 3.7 Hz, 2H); ^{13}C NMR (101 MHz, Chloroform-*d*) δ 177.4, 158.4, 152.3, 135.8, 129.4, 127.6, 126.7, 123.7, 108.1; ESI-MS (m/z) calculated for $C_{11}H_8O_2Cl$ 207.0213 and for $C_{11}H_7O_2ClNa$ 229.0032; found 207.0207 ([M+H]$^+$); 229.0030 ([M+Na]$^+$). Characterisation data is consistent with literature.[22]

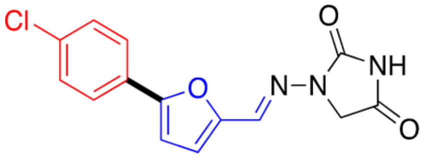

8n2: **8n** (72.2 mg, 0.35 mmol), 1-aminohydantoin (106.1 mg, 0.70 mmol) and 12 mL of water were added to a reaction vessel together and heated for 24 hours at 70 °C. The precipitate was then filtered, and washed with ice-cold water (3 x 15 mL). The product was dried *in vacuo* to give a brown powder;[23] Yield: 91%; FTIR (ATR) / cm^{-1}: 3284, 2981, 1802, 1773, 1763, 1710, 1623, 1599, 1478, 1444, 1413, 1392, 1342, 1279, 1235, 1211, 1201, 1115, 1094, 1026, 975, 937, 922, 898, 826, 791, 700, 685, 667, 645, 630, 614, 600; ^{1}H NMR (400 MHz, DMSO-d_6) δ 11.29 (s, 1H), 7.79 (d, *J* = 8.7 Hz, 2H), 7.73 (s, 1H), 7.53 (d, *J* = 8.7 Hz, 2H), 7.18 (d, *J* = 3.6 Hz, 1H), 6.96 (d, *J* = 3.6 Hz, 1H), 4.35 (s, 2H); ^{13}C NMR (101 MHz, DMSO-d_6) δ 206.6, 169.0, 153.3, 153.3, 149.5, 132.9, 132.6, 129.2, 128.4, 125.6, 115.5, 109.0, 49.0, 30.7; ESI-MS (m/z)

calculated for $C_{14}H_{11}O_3ClN_3$ 304.0489 and for $C_{14}H_{10}O_3ClN_3Na$ 326.0308; found 304.0483 ($[M+H]^+$); 326.0303 ($[M+Na]^+$); Crystals were grown from a saturated solution by slow evaporation of MeCN. Characterisation data is consistent with literature.[24]

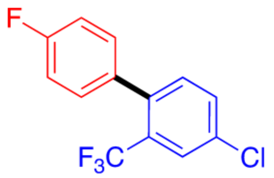

4o: colourless oil; Yield: 94%; FTIR (thin film) / cm^{-1}: 3093, 1601, 1512, 1498, 1481, 1403, 1304, 1232, 1173, 1159, 1125, 1064, 1008, 892, 827, 811, 729, 665, 581, 536, 517, 472; ^{1}H NMR (400 MHz, Chloroform-*d*) δ 7.73 (d, *J* = 2.2 Hz, 1H), 7.57 – 7.52 (m, 1H), 7.32 – 7.23 (m, 3H), 7.16 – 7.03 (m, 2H); ^{19}F NMR (376 MHz, Chloroform-*d*) δ -57.34, -114.02 (td, *J* = 8.7, 4.4 Hz); ^{13}C NMR (101 MHz, Chloroform-*d*) δ 162.8 (d, *J* = 247.3 Hz), 139.0 (d, *J* = 2.1 Hz), 134.6 (d, *J* = 3.7 Hz), 133.9, 133.7, 131.6, 130.8 (dd, *J* = 8.1, 1.7 Hz), 130.2 (d, *J* = 30.6 Hz), 126.6 (q, *J* = 5.7 Hz), 123.4 (d, *J* = 274.4 Hz), 115.1 (d, *J* = 21.6 Hz). GC-EI (m/z) calculated for $C_{13}H_7ClF_4$ 274.0172; found 274.0154 (M^+). Characterisation data is consistent with literature.[25]

S3 T_{gel} Values

Table S1. T_{gel} values for the different gel-systems

Sample	T_{gel} / ºC
DBS-CONHNH$_2$	96.6 ± 2.0
Agarose	98.1 ± 0.5
Hybrid	98.9 ± 0.3
Agarose Pd	95.3 ± 1.6
Hybrid Pd	96.6 ± 2.0

S4 Metal Uptake

S4.1 Calibration curve for $PdCl_2$

Table S2. Pd(II) Concentration versus absorbance used to generate a calibration curve for a Pd^{II} aqueous solution.

$[Pd^{II}]$ / mM	Absorbance[A]
5.0	1.087
4.5	0.969
4.0	0.883
3.5	0.767
3.0	0.678
2.5	0.543
2.0	0.443
1.5	0.320
1.0	0.220
0.5	0.098
0.0	0.000

[A] Absorbance was monitored at 421 nm.

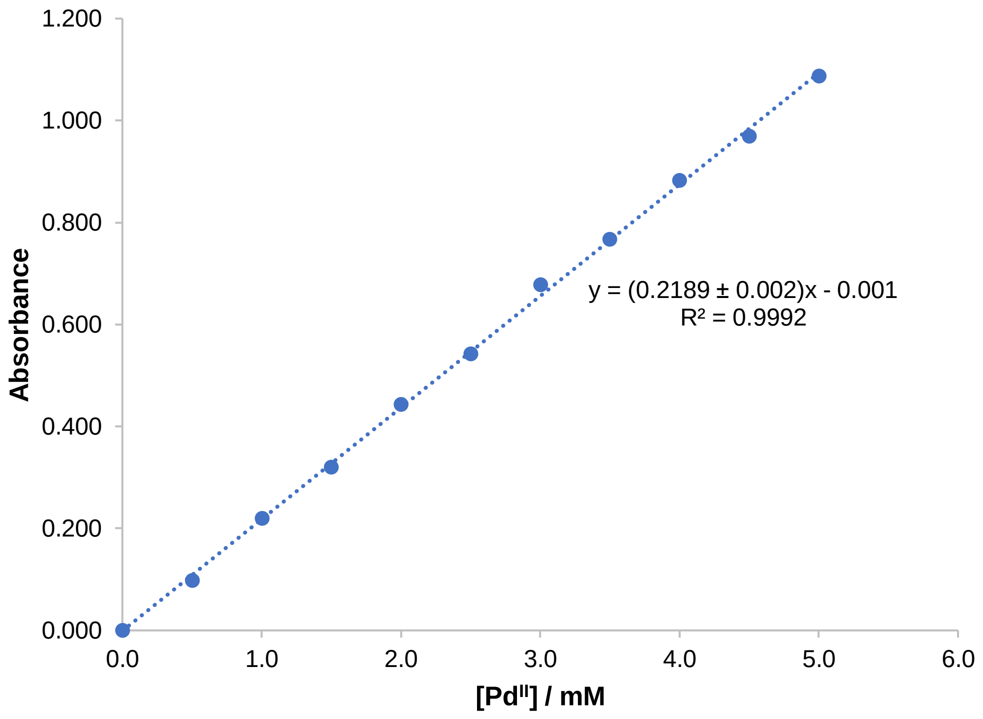

Figure S1. Absorbance at 421 nm versus concentration calibration curve used to find the concentration of $[Pd^{II}]$ in solution. (Solvent = H_2O)

S4.2 Metal Uptake Over Time

Table S3. Mean absorbance of the [Pd^{II}] aqueous solution exposed to different gel systems over time.

		Absorbance		
		**DBS-CONHNH$_2$**	Hybrid	Agarose
Time / min	0	1.081	1.081	1.081
	30	0.65 ± 0.02	0.961 ± 0.11	0.955 ± 0.003
	60	0.55 ± 0.03	0.969 ± 0.007	0.916 ± 0.011
	120	0.62 ± 0.04	0.94 ± 0.02	0.883 ± 0.006
	270	0.63 ± 0.07	0.94 ± 0.02	0.899 ± 0.005
	390	0.44 ± 0.03	0.93 ± 0.03	0.887 ± 0.006
	1440	0.57 ± 0.12	0.962 ± 0.011	0.918 ± 0.003
	1620	0.51 ± 0.04	0.953 ± 0.012	0.914 ± 0.006
	2880	0.32 ± 0.03	0.933 ± 0.008	0.914 ± 0.007
	3090	0.30 ± 0.04	0.938 ± 0.003	0.913 ± 0.010
	3270	0.27 ± 0.06	0.940 ± 0.002	0.923 ± 0.008
	4320	0.34 ± 0.03	0.935 ± 0.004	0.932 ± 0.011

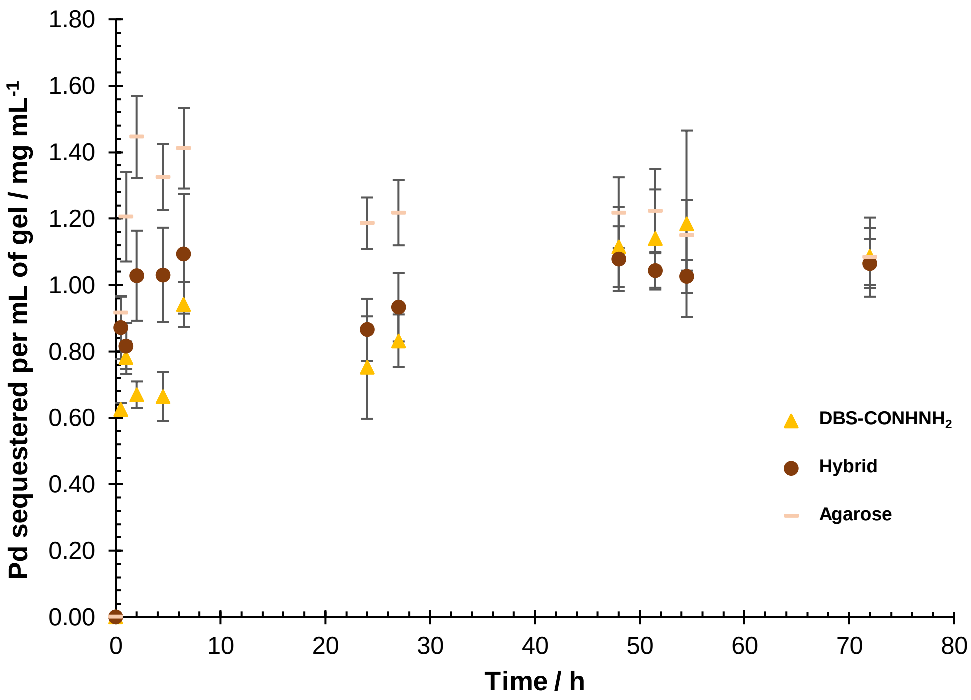

Figure S2. Absorbance at 421 nm versus time for different samples. (Solvent = H_2O)

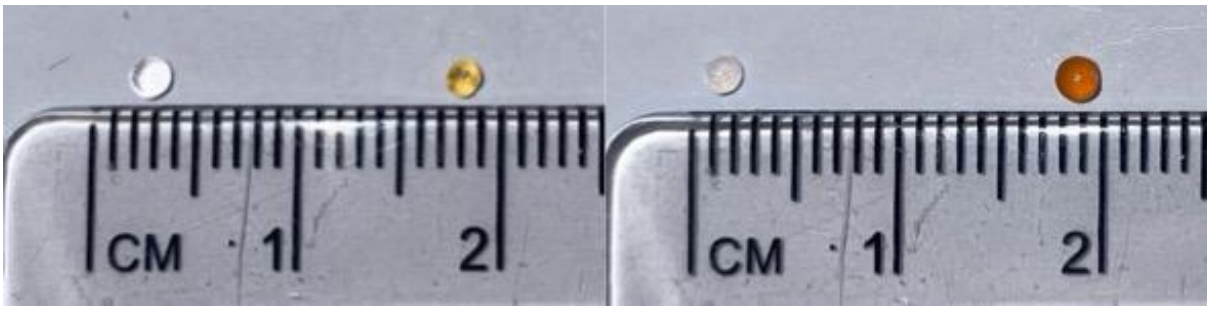

Figure S3. 5 µL agarose beads before and after being exposed to Pd^{II} solution (left), and 5 µL hybrid beads before and after being exposed to Pd^{II} solution.

S5 SEM and TEM

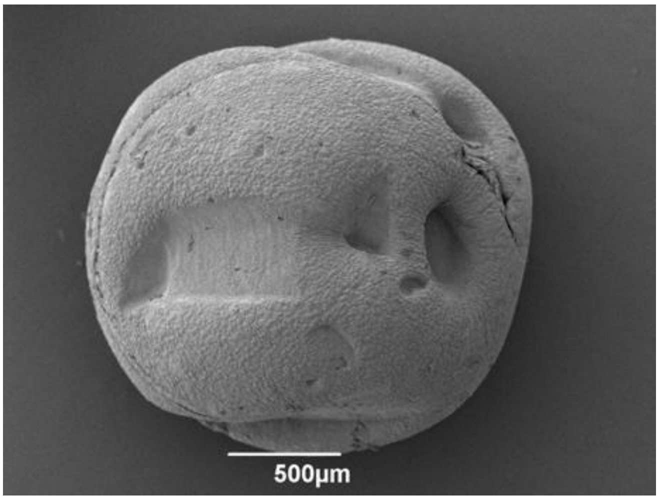

Figure S4. SEM image of a full hybrid bead after being exposed to Pd^{II} solution

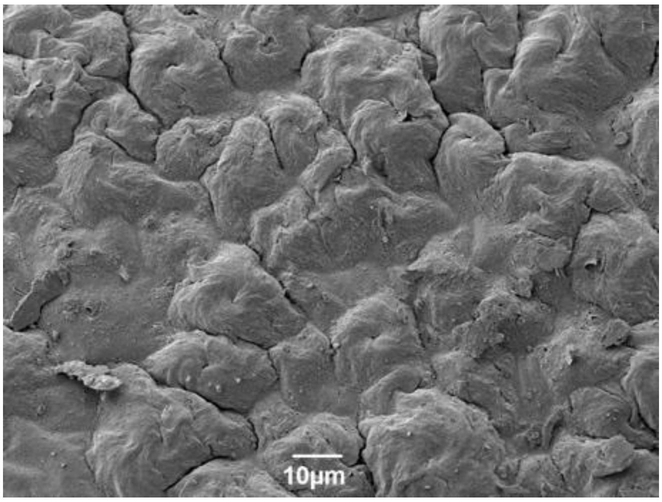

Figure S5. SEM image of the surface of a hybrid bead after being exposed to Pd^{II} solution

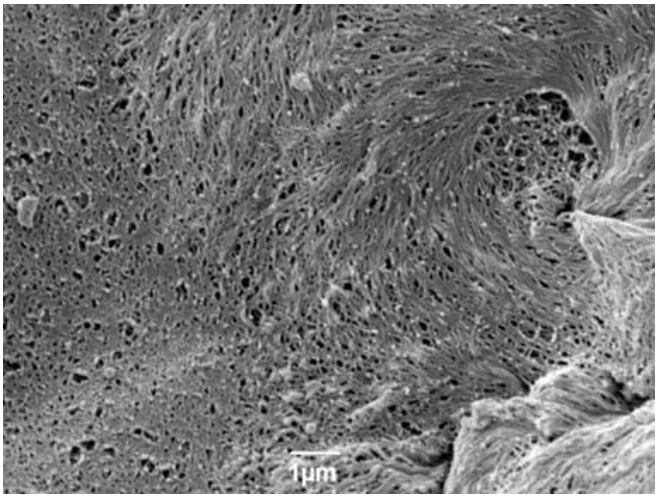

Figure S6. SEM image of the inside of a hybrid bead after being exposed to Pd^{II} solution

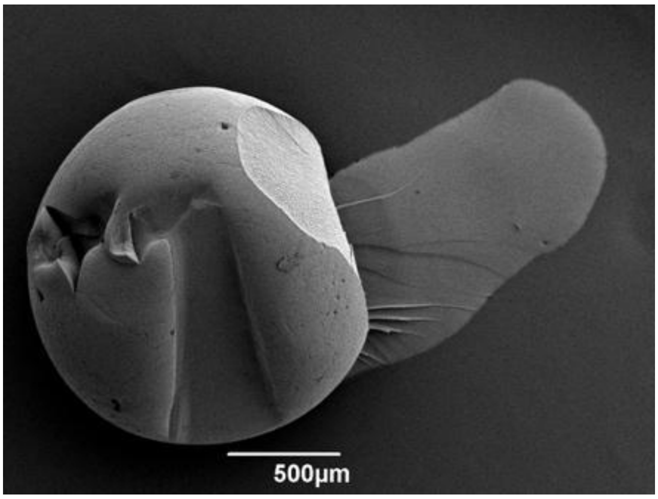

Figure S7. SEM image of a full agarose bead after being exposed to Pd^{II} solution

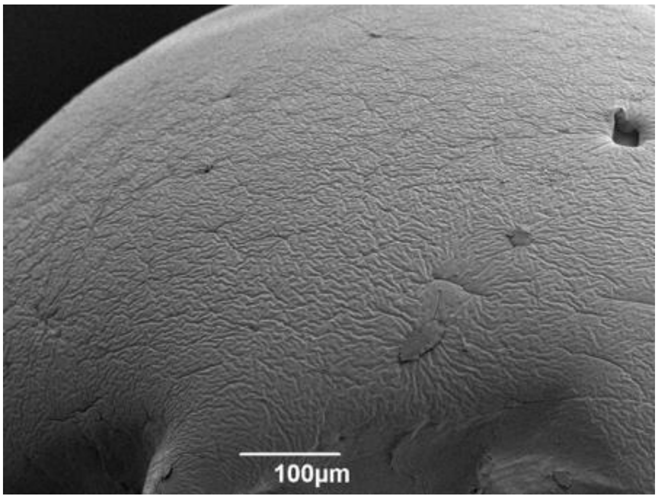

Figure S8. SEM image of the surface of an agarose bead after being exposed to Pd^{II} solution

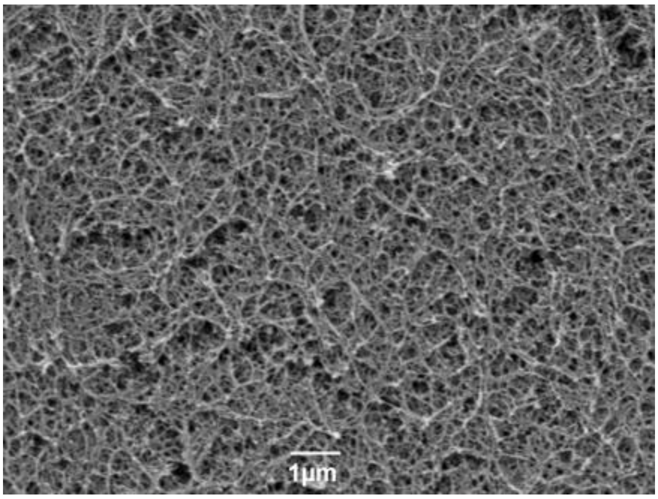

Figure S9. SEM image of the inside of an agarose bead after being exposed to Pd^{II} solution

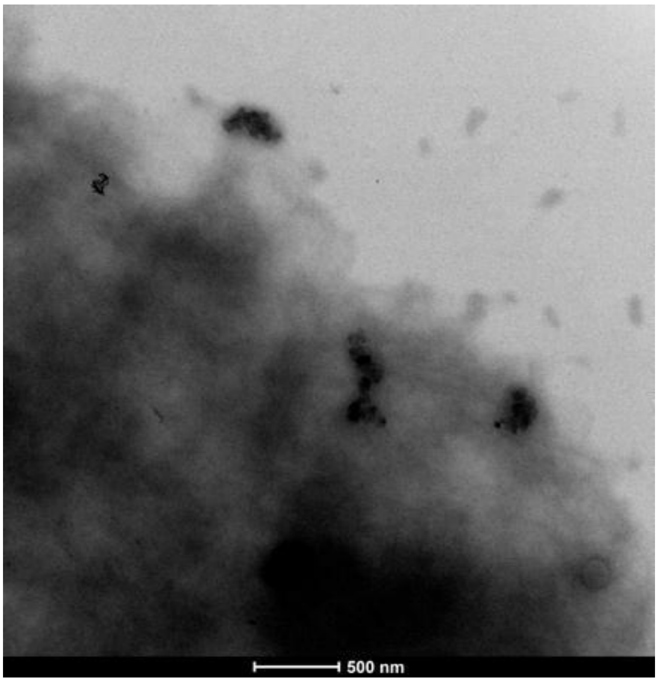

Figure S10. TEM image of a hybrid bead after being exposed to Pd^{II} solution showing NPs

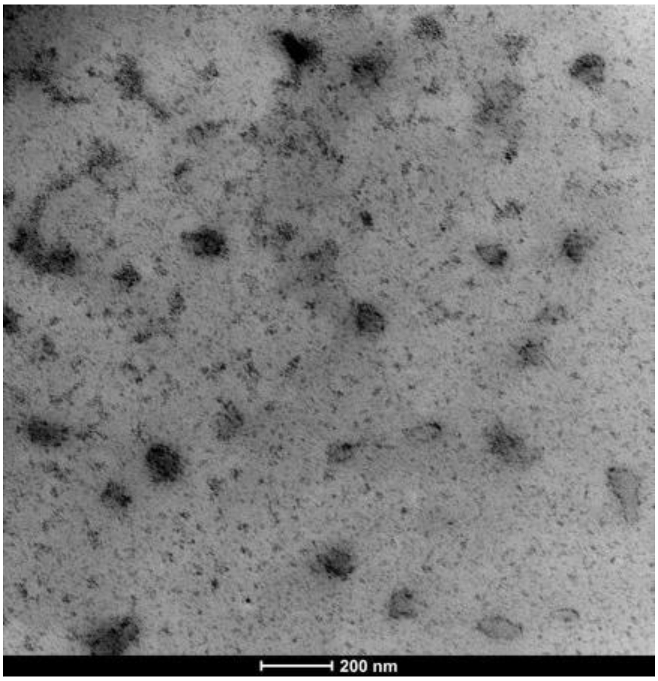

Figure S11. TEM image of a hybrid bead after being exposed to Pd^{II} solution showing NPs

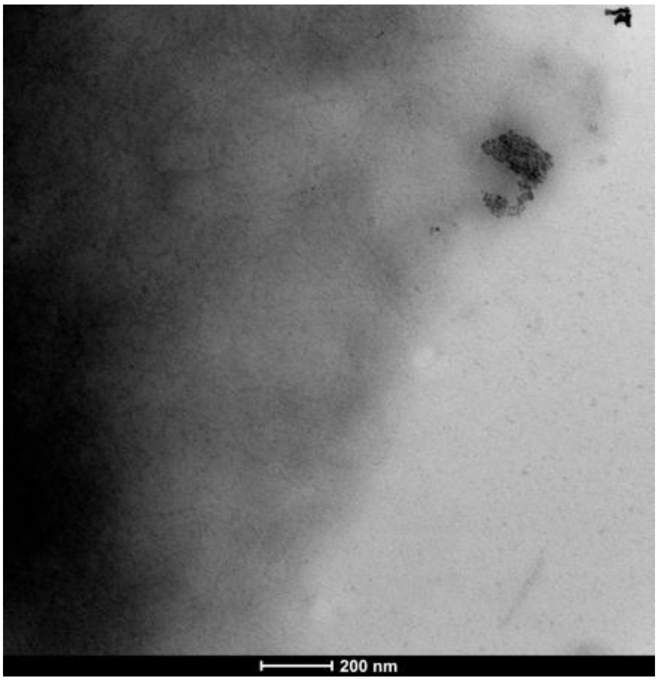

Figure S12. TEM image of a hybrid bead after being exposed to Pd^{II} solution showing NPs

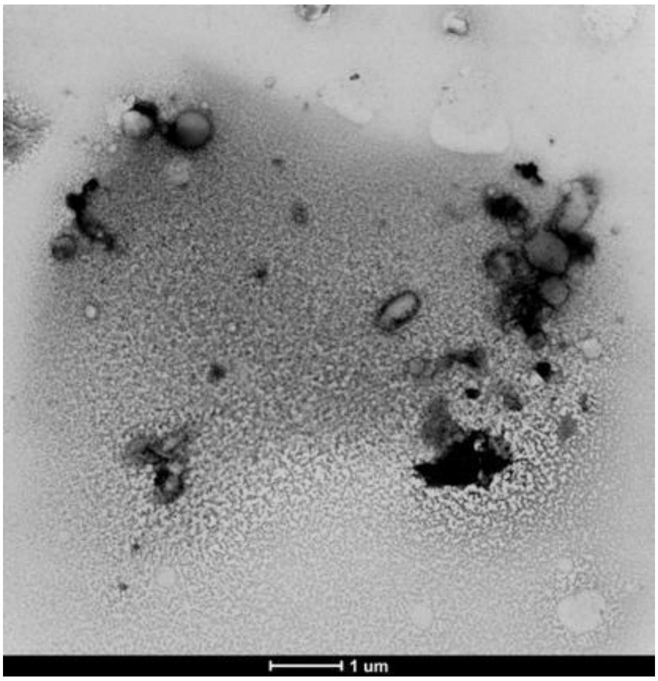

Figure S13. TEM image of an agarose bead after being exposed to Pd^{II} solution showing some evidence of NPs formation together with larger Pd–aggregates

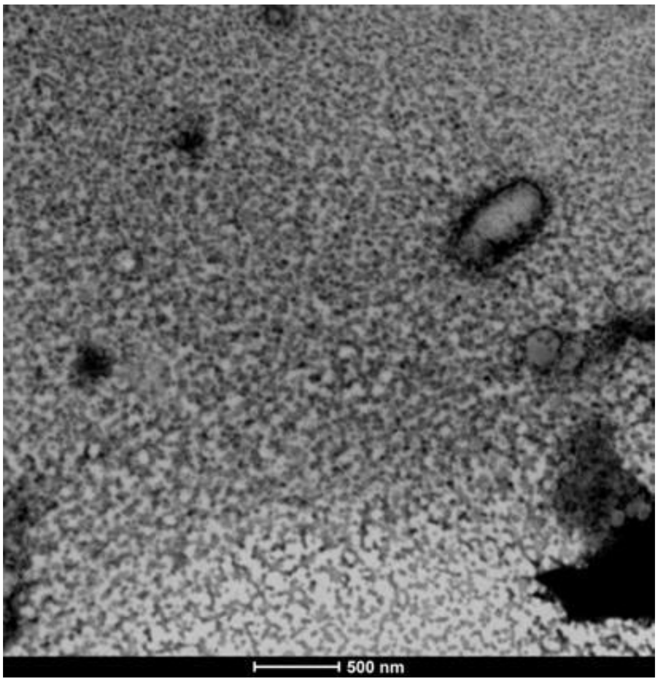

Figure S14. TEM image of an agarose bead after being exposed to Pd^{II} solution showing some evidence of NPs

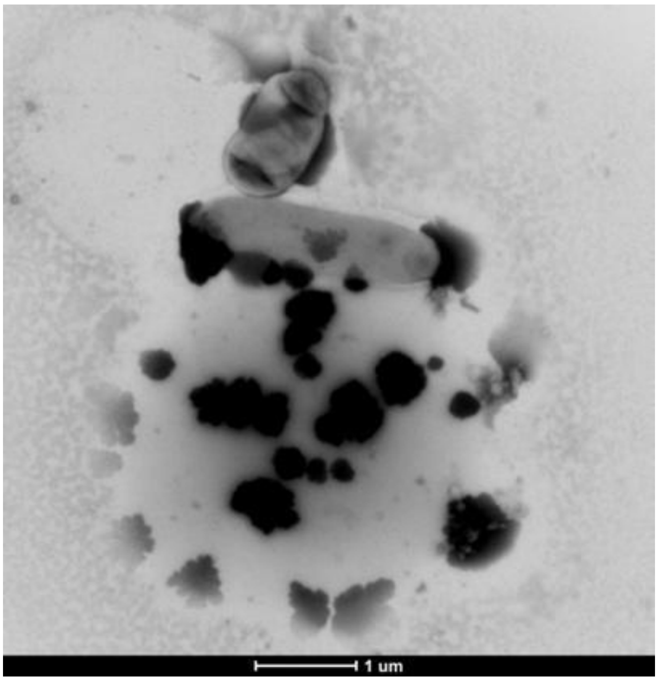

Figure S15. TEM image of an agarose bead after being exposed to Pd^{II} solution showing large Pd–aggregates

S5.1 NP Size and Frequency

Table S4. Distribution of NPs size in the hybrid system. Data derived from analysis of Fig. S11.

Diameter, x / nm	Frequency
x ≤ 3	162
3 < x < 7	19
7 < x < 9	2
9 < x < 11	2
11 < x < 15	4
15 < x < 25	3
25 < x	8

Table S5. Distribution of NPs size in agarose. Data derived from analysis of Fig. S14.

**Diameter, x / nm**	**Frequency**
x ≤ 3	388
3 < x ≤ 7	193
7 < x ≤ 9	52
9 < x ≤ 11	54
11 < x ≤ 15	129
15 < x ≤ 25	220
25< x ≤ 100	173

S6 Oxidation assays with **DBS-CONHNH$_2$**

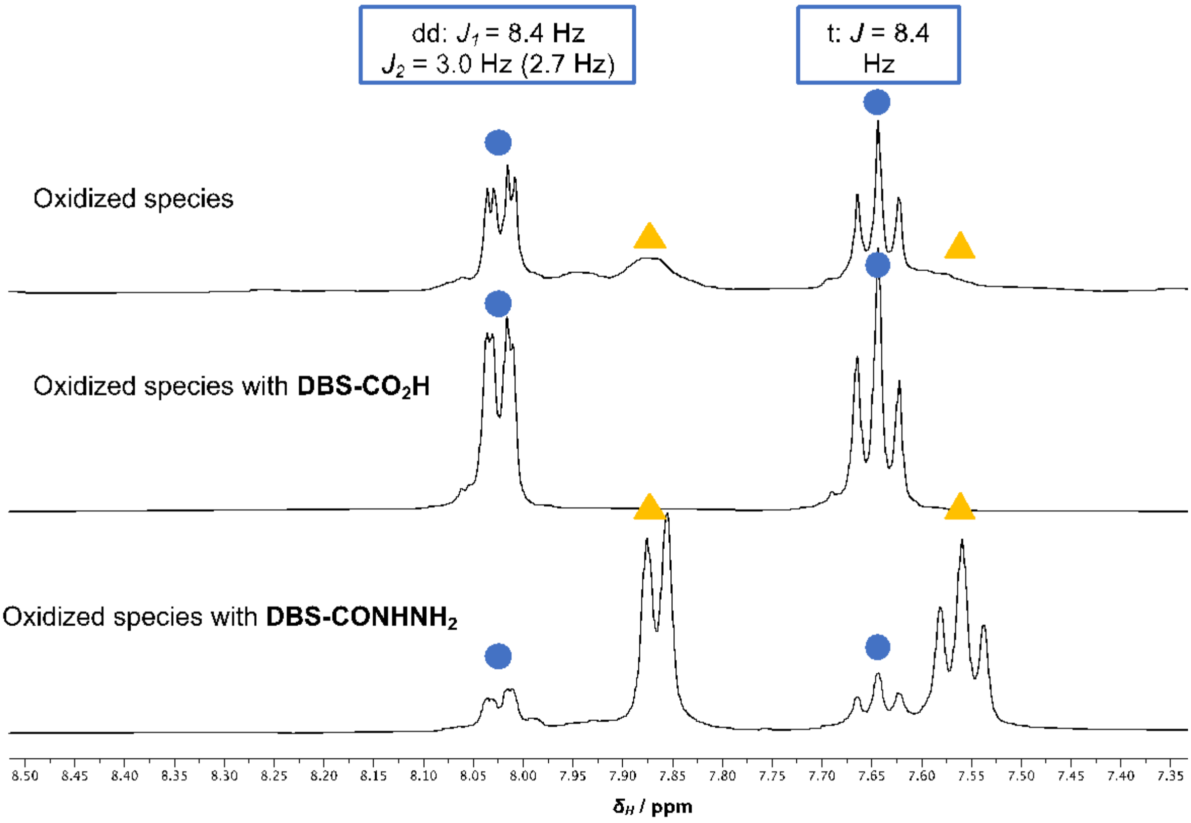

Figure S16. Stacked NMR spectra in DMSO-d6 of the oxidised sample (top), the oxidised sample spiked with **DBS-CO$_2$H** (middle) and lastly the oxidised sample spiked with **DBS-CONHNH$_2$** (bottom).

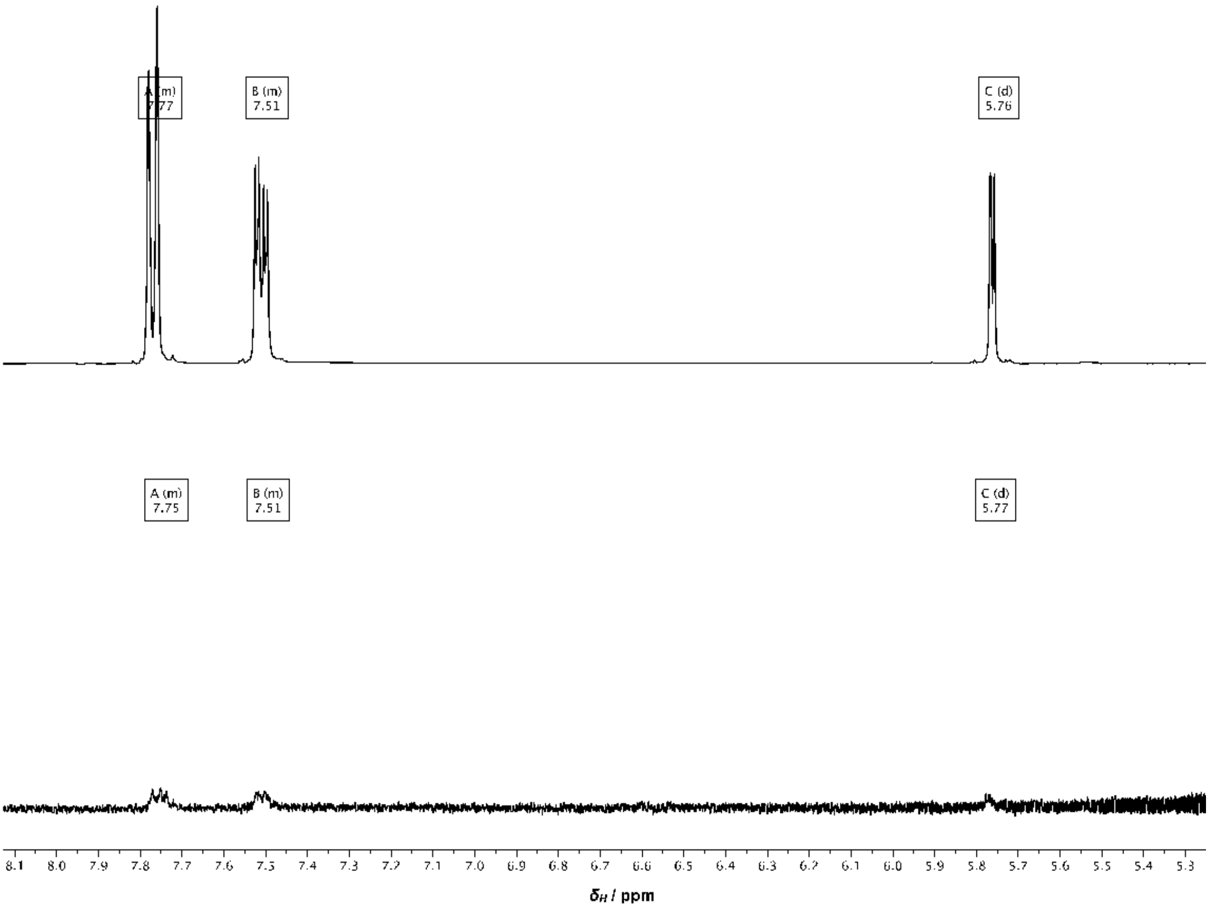

Figure S17. NMR of **DBS-CO_2H** (top) and product of the oxidation of **DBS-CONHNH_2** (bottom) in 0.1 M NaOD in D_2O

S7 NMR Studies on Gel Systems

S7.1 Proof of Gelation

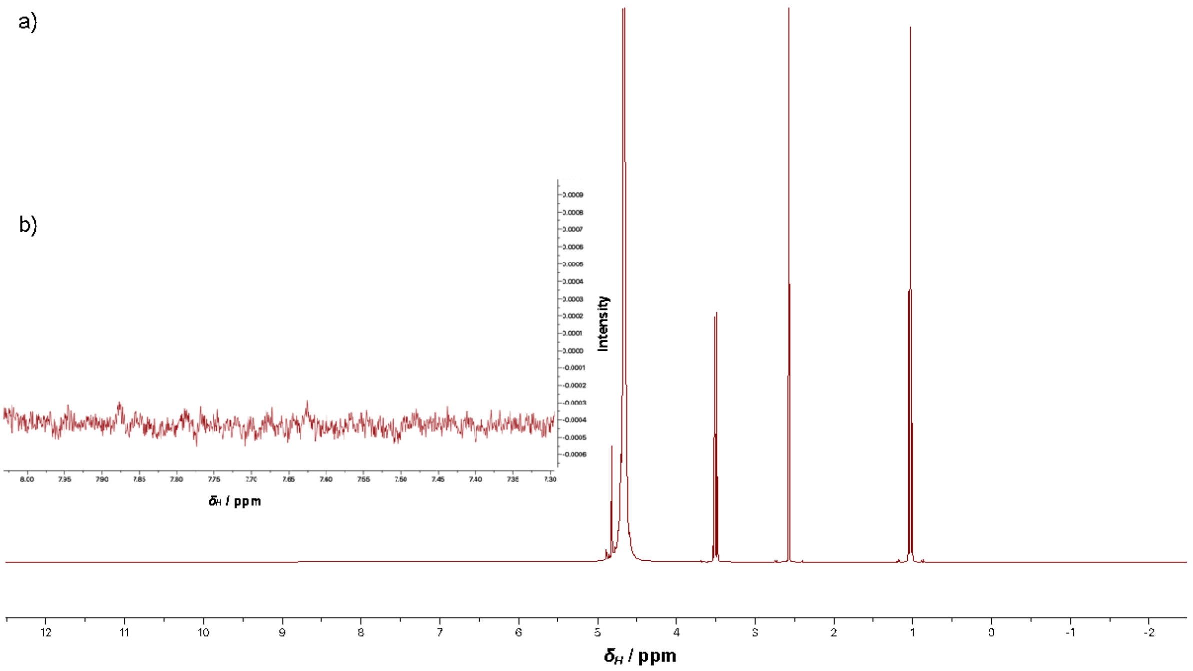

Figure S18 a) 400 MHz ^{1}H NMR of hybrid beads in D_2O clearly showing the silent aromatic region in b)

S7.2 Thermal Stability

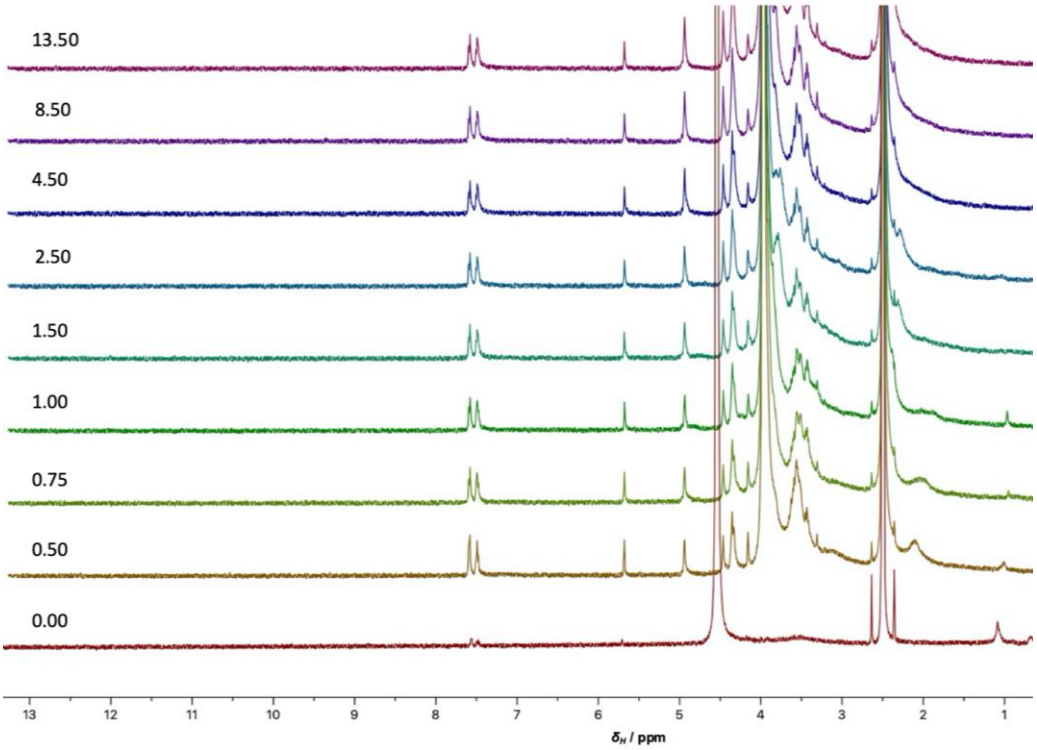

Figure S19. 400 MHz ^{1}H NMR stacked spectra in D_2O showing gradual thermal degradation of the hybrid beads with **DBS-CONHNH$_2$** leaching out. Time in hours is noted on left hand side of the graph

Table S6. Mass of **DBS-CONHNH$_2$** present in solution versus time

Time / min	Mass of DBS-CONHNH$_2$ in solution / mg
0	0.026
30	0.188
45	0.196
60	0.199
90	0.199
150	0.199
270	0.199
510	0.196
810	0.195

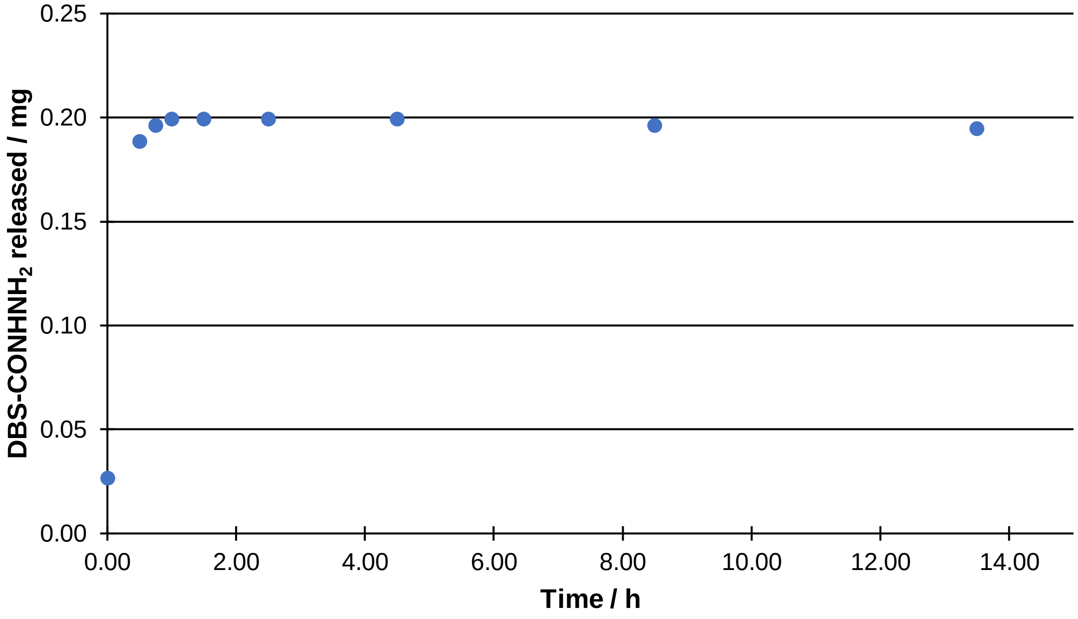

Figure S20. Degradation of the hybrid beads over time at 90 ºC monitored *via* **DBS-CONHNH$_2$** leaching quantified using ^{1}H NMR.

S7.3 Quantification of **DBS-CONHNH$_2$** Content in Hybrid Beads

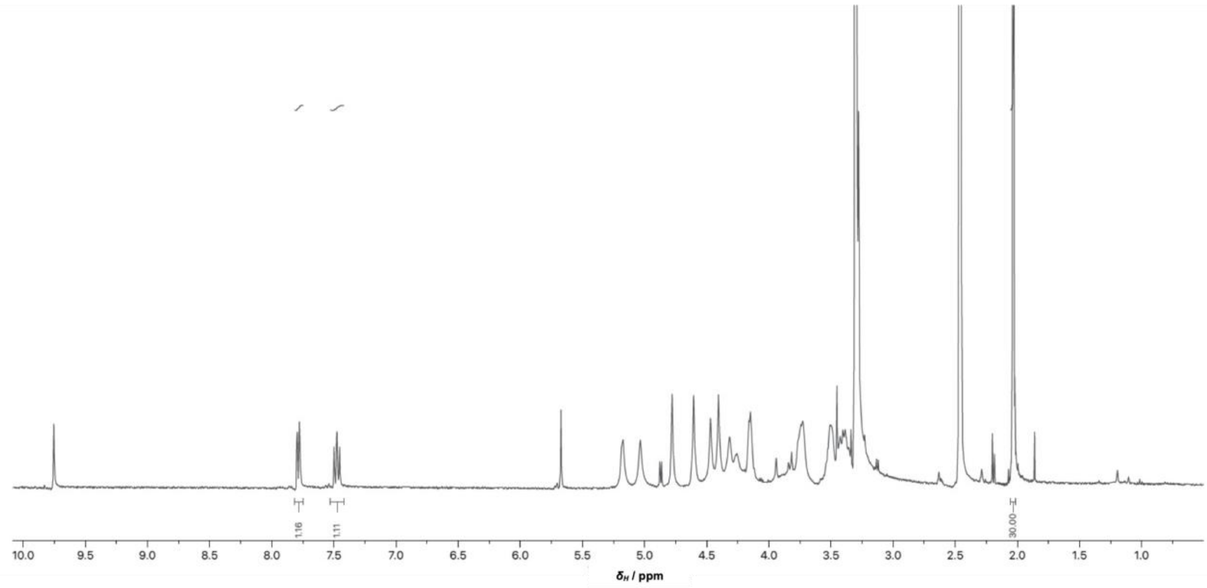

Figure S21. 400 MHz ^{1}H NMR of the dried hybrid beads in DMSO-d_6 used to calculate the amount of **DBS-CONHNH$_2$** incorporated in the former

S8 IR of Gel Systems

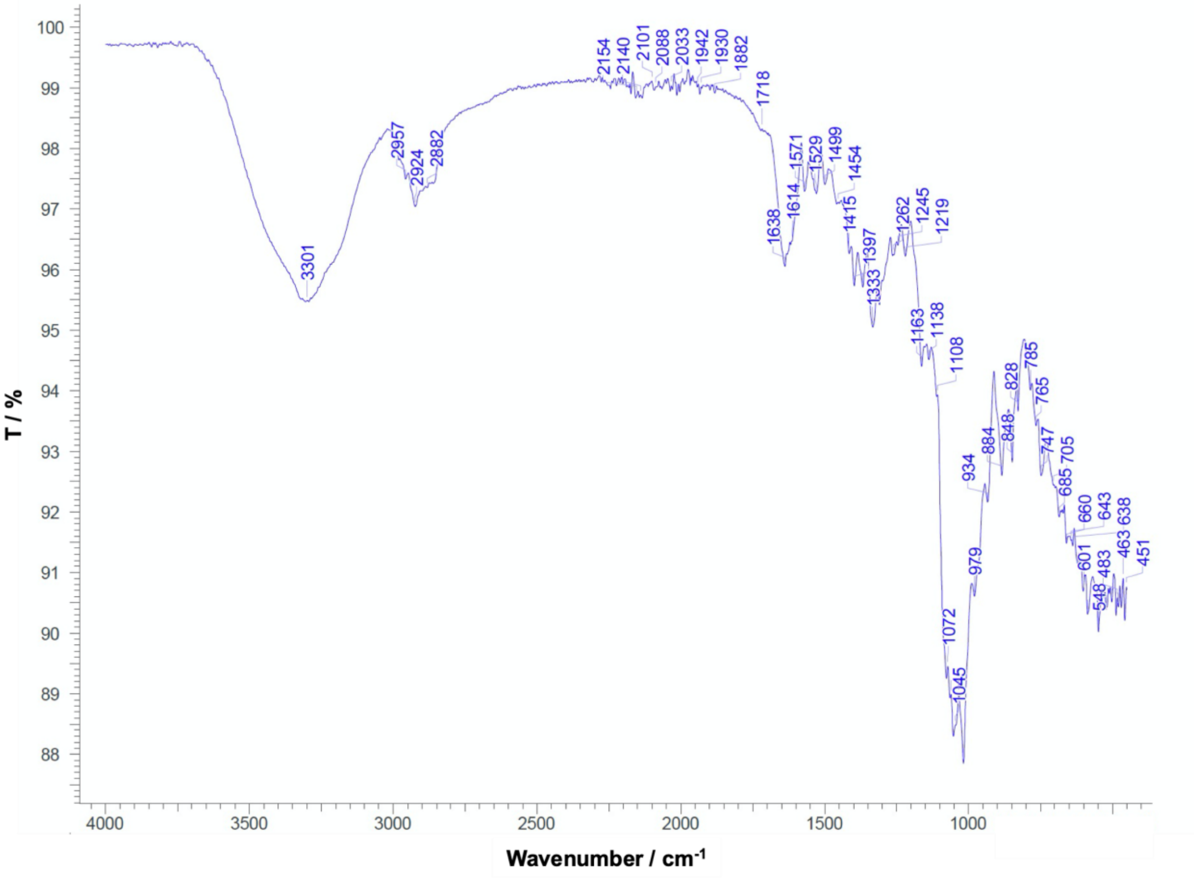

Figure S22. FTIR spectrum of dried **DBS-CONHNH$_2$** prepared using the standard procedure.

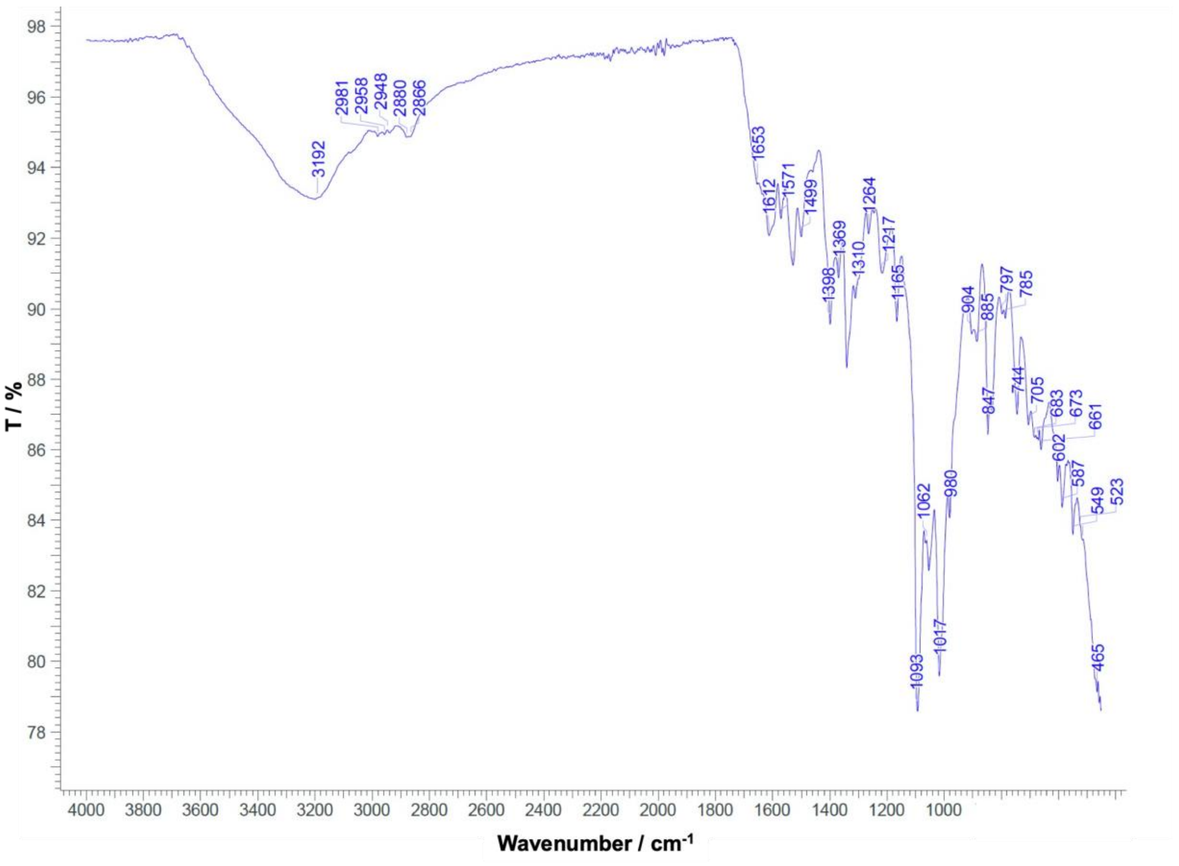

Figure S23. FTIR spectrum of dried **DBS-CONHNH$_2$** prepared from the standard procedure, with Pd.

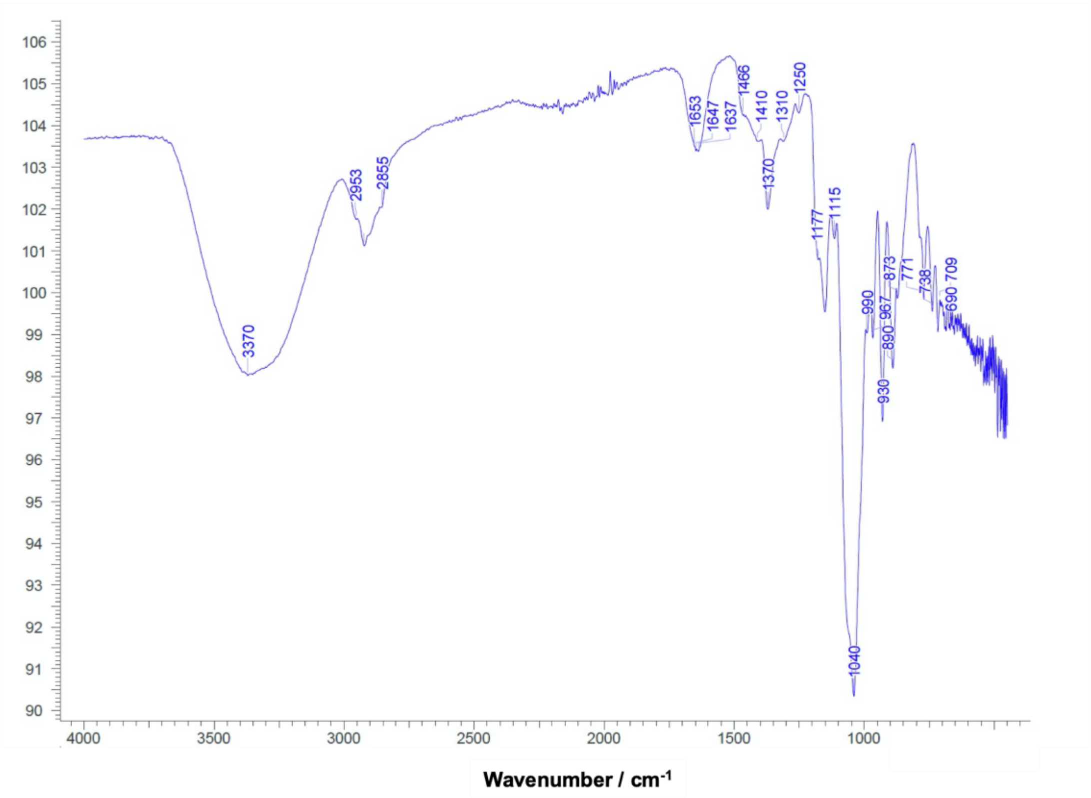

Figure S24. FTIR spectrum of dried agarose beads prepared from the standard procedure.

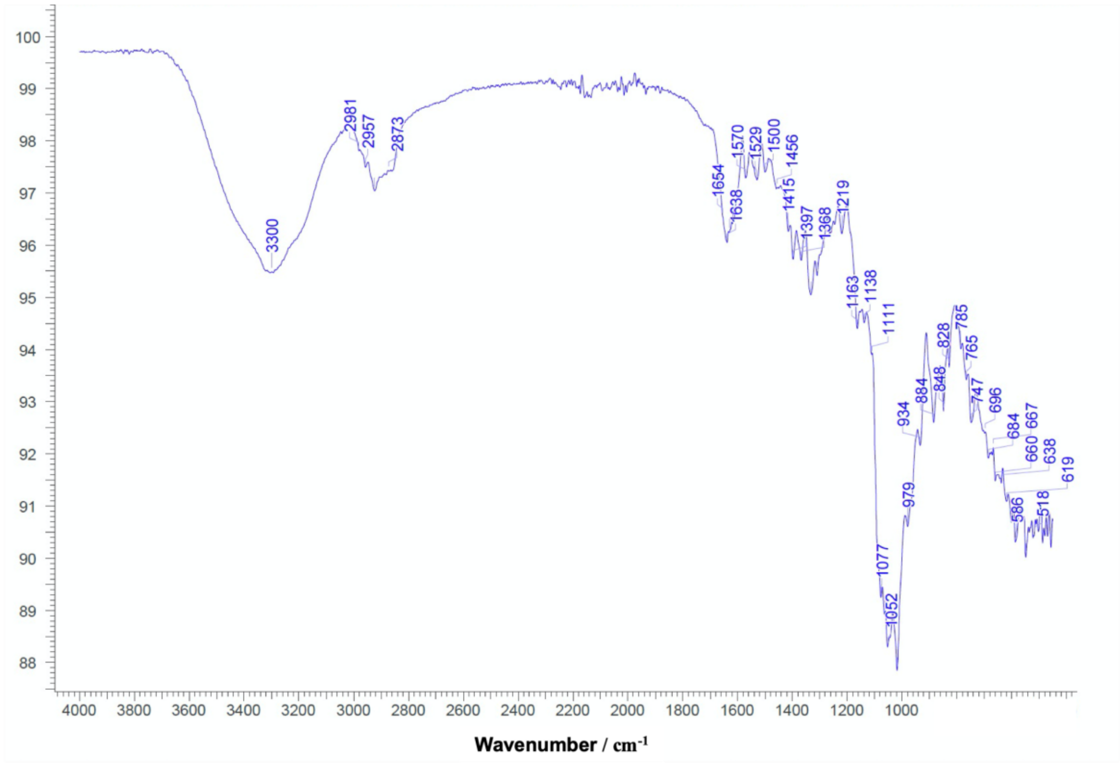

Figure S25. FTIR spectrum of dried agarose beads prepared from the standard procedure with Pd

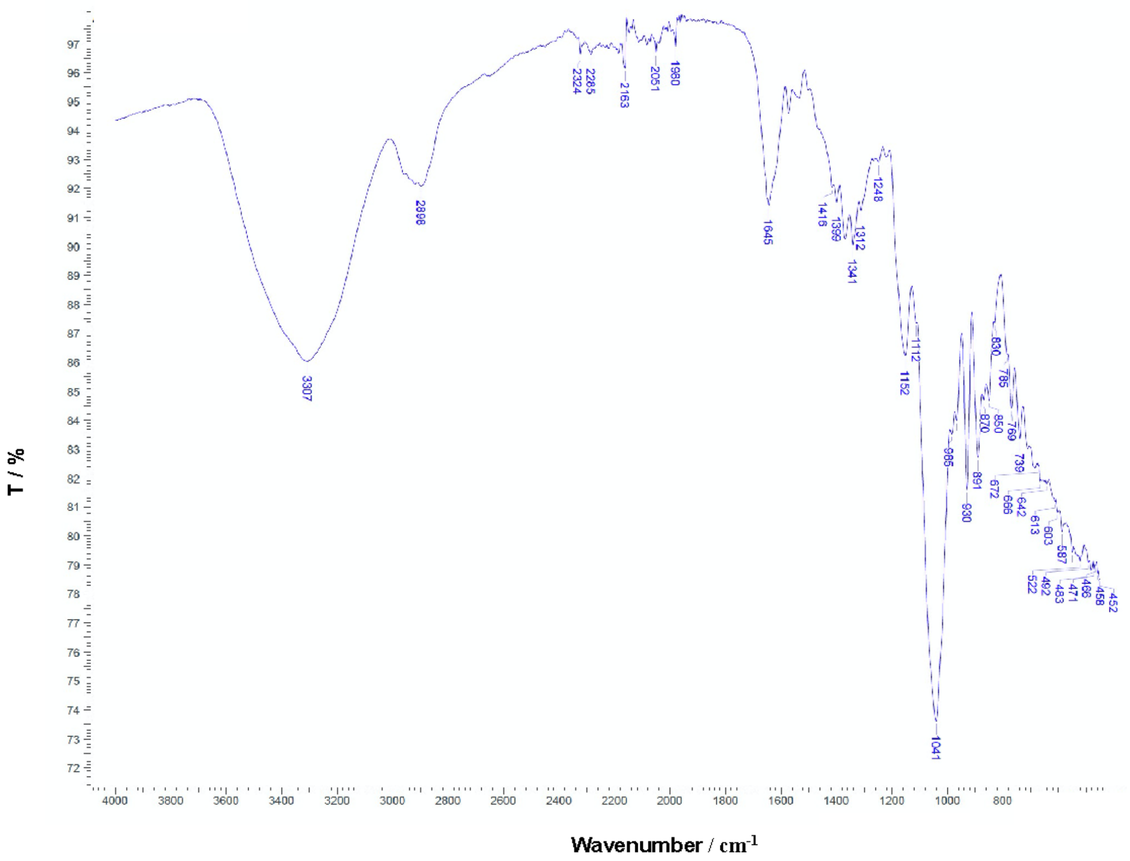

Figure S26. FTIR spectrum of dried hybrid beads prepared from the standard procedure.

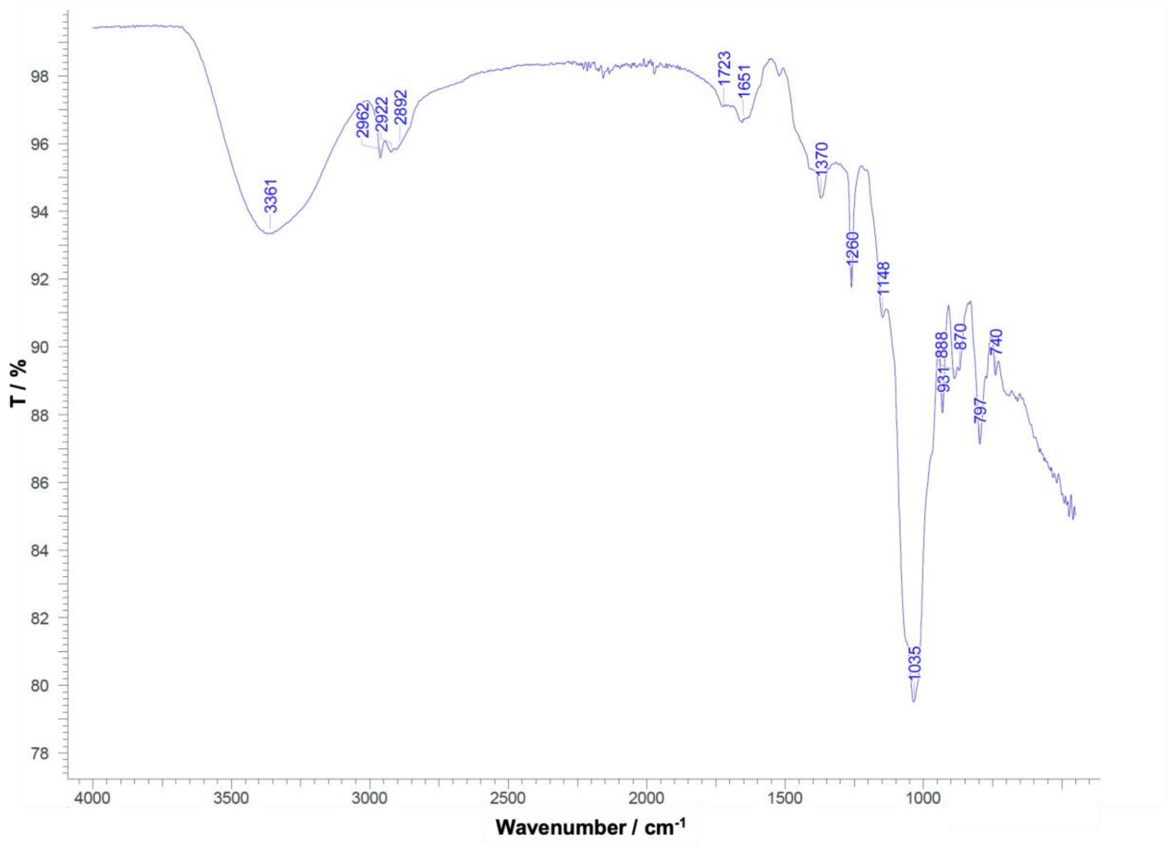

Figure S27. FTIR spectrum of dried hybrid beads prepared from the standard procedure with Pd.

S9 Rheology

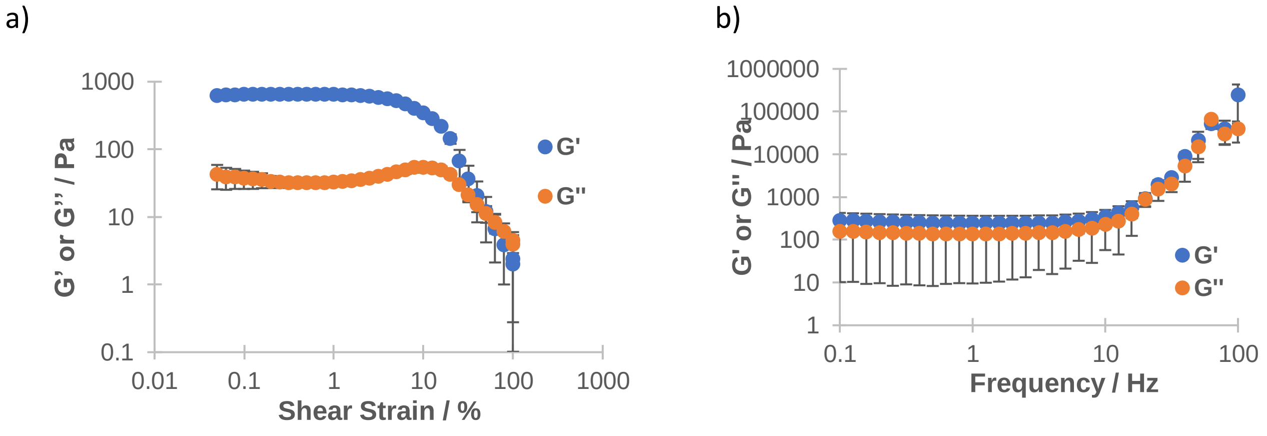

Figure S28. a) Amplitude sweep (constant frequency = 1 Hz) and b) frequency sweep (constant shear strain 0.05%) for **DBS-CONHNH$_2$** prepared using the standard procedure.

a) b)

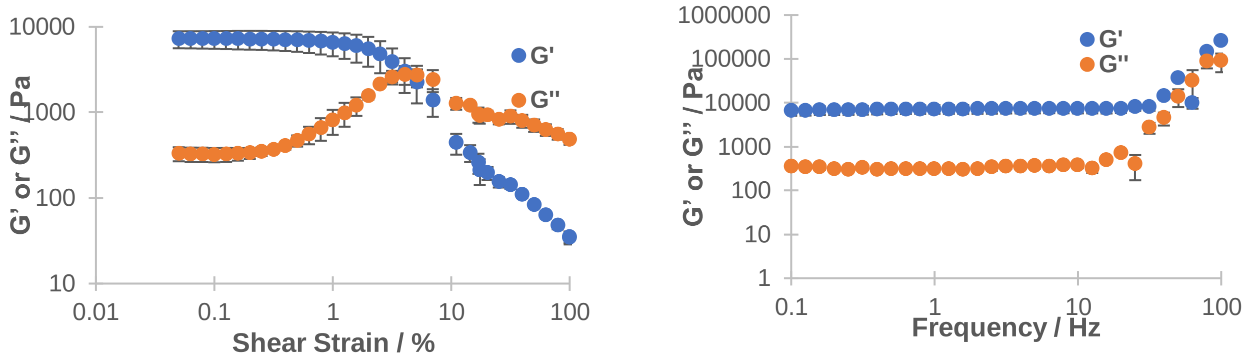

Figure S29. a) Amplitude sweep (constant frequency = 1 Hz) and b) frequency sweep (constant shear strain 0.05%) for agarose prepared using the standard procedure.

a) b)

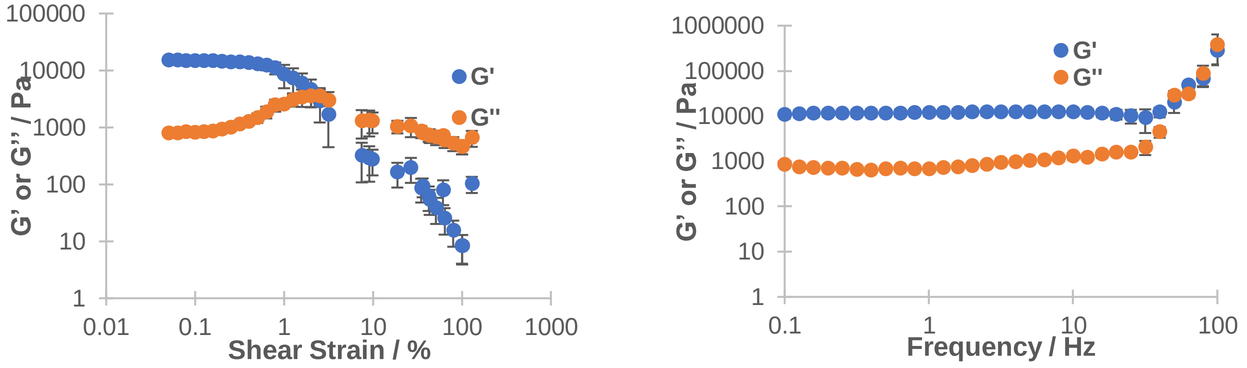

Figure S30. a) Amplitude sweep (constant frequency = 1 Hz) and b) frequency sweep (constant shear strain 0.05%) for the hybrid system prepared using the standard procedure.

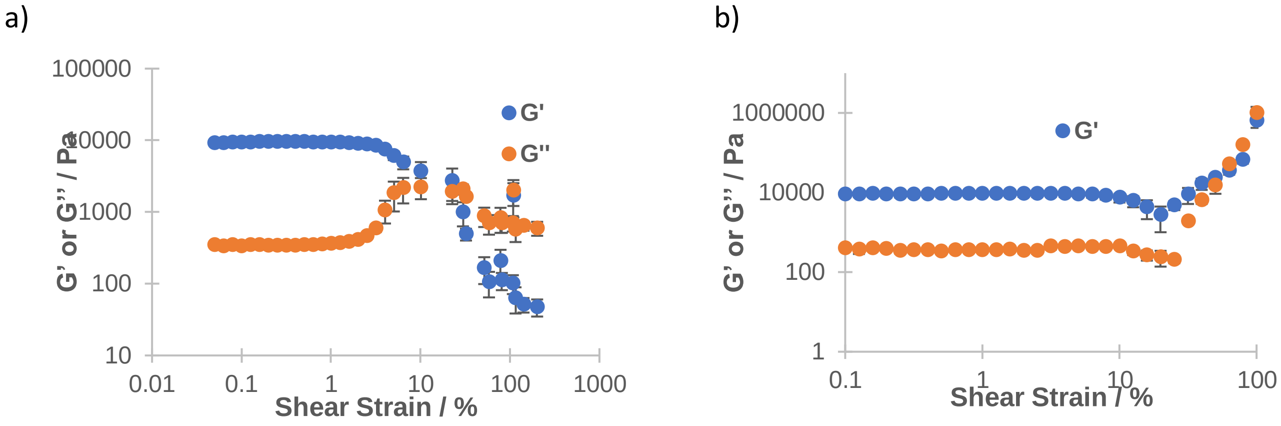

Figure S31. a) Amplitude sweep (constant frequency = 1 Hz) and b) frequency sweep (constant shear strain 0.05%) for the agarose with Pd prepared using the standard procedure.

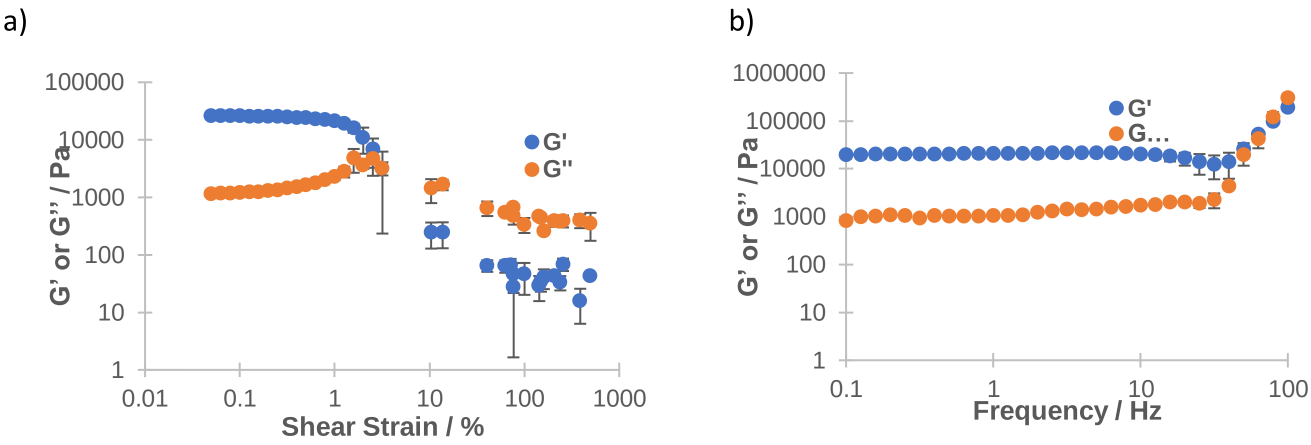

Figure S32. a) Amplitude sweep (constant frequency = 1 Hz) and b) frequency sweep (constant shear strain 0.05%) for the agarose with Pd prepared using the standard procedure.

Table S7. Summary of the values for G' and G'' for the systems studied in this investigation

	G' / Pa	G'' / Pa
**DBS-CONHNH$_2$**	255 ± 4	143 ± 2
Agarose	7120 ± 60	333 ± 6
Hybrid	12000 ± 100	830 ± 40
Agarose Pd	9280 ± 40	377 ± 8
Hybrid Pd	20610 ± 110	11800 ± 50

S10 Process Optimisation

S10.1 Solvent System

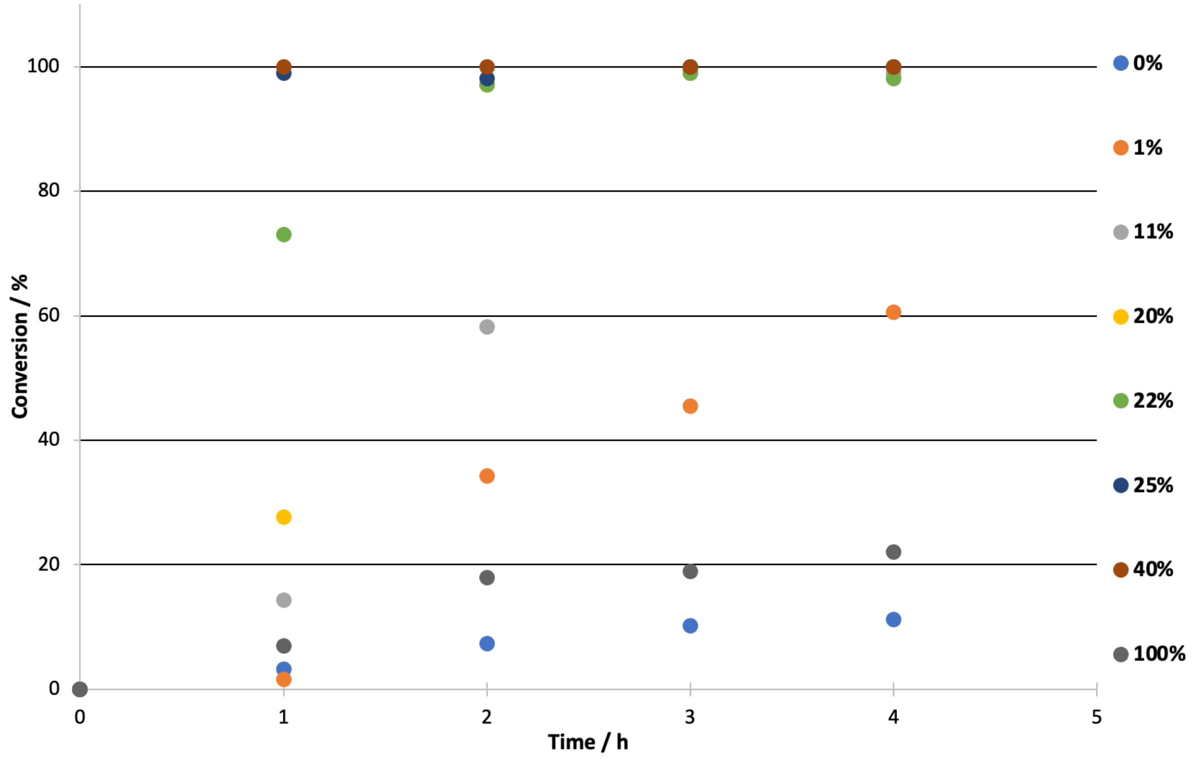

Figure S33. Conversion versus time for the different solvent systems investigated in the screening process at 50 °C with 0.1 mol% Pd

S10.2 Temperature

Table S8. Conversion versus temperature after 1 hour with 0.1 mol % Pd in 1:3 EtOH-H_2O

T / °C	Time / h
	1
25	40
50	99
70	99
80	N.A.

S10.3 Catalyst Loading

Table S9. Conversion versus catalyst loading after 1 hour at 50 °C in 1:3 EtOH-H_2O

Pd / mol % (No. bead)	Time / h
	1
0.025 (1)	99
0.050 (2)	99
0.100 (4)	99

S10.4 Process Optimisation for Aryl Bromides and Chlorides

Since aryl chlorides and bromides are notoriously more difficult to activate than their iodide counterparts, a temperature screening including higher reaction temperature (70 and 80 °C) was performed on **a’** using 0.10 mol % catalyst loading, and 25% EtOH in water. Unfortunately, at 80 °C the beads melted, however, at 70 °C, full conversion was achieved in 4 hours, significantly shorter compared to 6 hours at 50 °C. For chlorides, no temperatures below 70 °C were investigated and the conversion reached 63% after 36 hours. Longer reaction times would have likely resulted in further conversion but the reaction was halted due to proof the system could activate aryl chlorides.

S11 Mechanistic Studies

S11.1 Mercury Drop Test Conversion Data

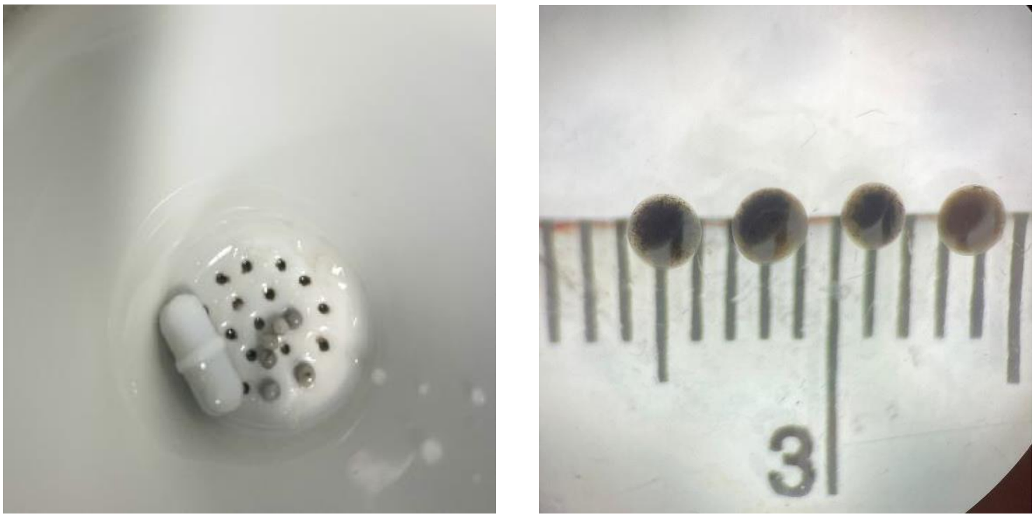

Figure S34. (left) Pd–Hybrid gel filtered out of reaction mixture and (right) after being washed visualized under a microscope

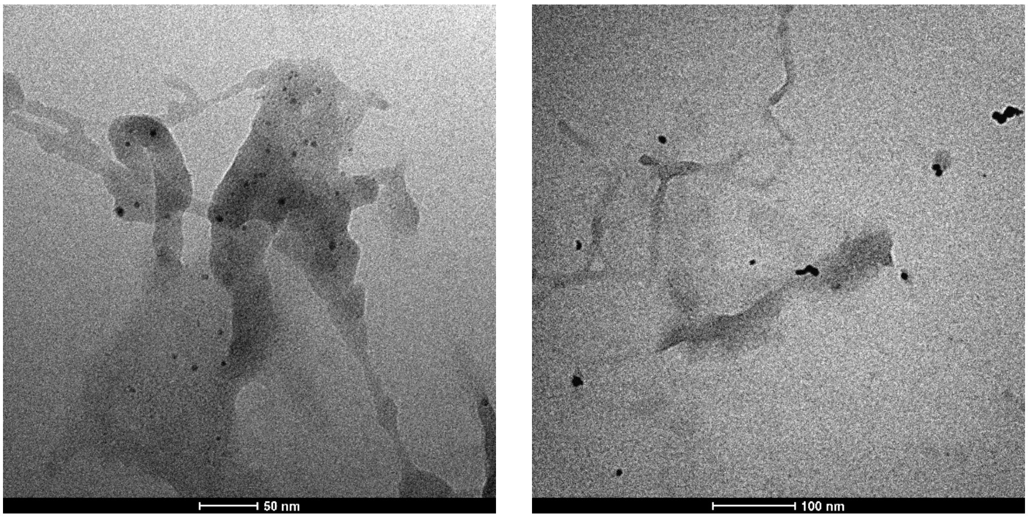

Figure S35. TEM of the Pd–NPs after (left) 1 run with Hg^0 and (right) 1 run without any Hg^0, both showing some level of aggregation but demonstrating indistinguishable appearance between the two.

Table S10. Conversion versus time for the reaction between 4'-iodoacetophenone, **a**, and 4-methoxyphenylboronic acid, **3**, under standard conditions and in the presence of Hg^0

Time / min	Conversion % for control run	Conversion % with Hg^0
0	0.0	0.00
3	7	5
6	18	17
9	28	50.00
12	33	/
15	50	/
20	57	71
40	91	87
50	97	93
60	100	98
70	100	99
80	100	100
90	100	100

S11.2 Resin Experiment Conversion Data

Table S11. Ratios of 4-tolylboronic acid and the internal standard, 1,3,5-trimethoxybenzene, versus time used to calculate conversion.

Time / h	4-tolylboronic acid (**1.0** eq.)		1,3,5-trimethoxybenzene (**0.5** eq.)		Ratio	Conversion
	Integral, I_A	(I_A) x 3 x **0.5**	Integral, I_B	(I_B) x 2 x **1.0**	R = (I_A) x 3 x **0.5**/(I_B) x 2 x **1.0**	(1 – R) x 100
0	1.0	1.5	0.75	1.50	1.0	0%
1	1.0	1.5	0.76	1.52	0.98	2%
4	1.0	1.5	0.76	1.52	0.98	2%

S11.3 2,4-Dibromopyridine Selectivity

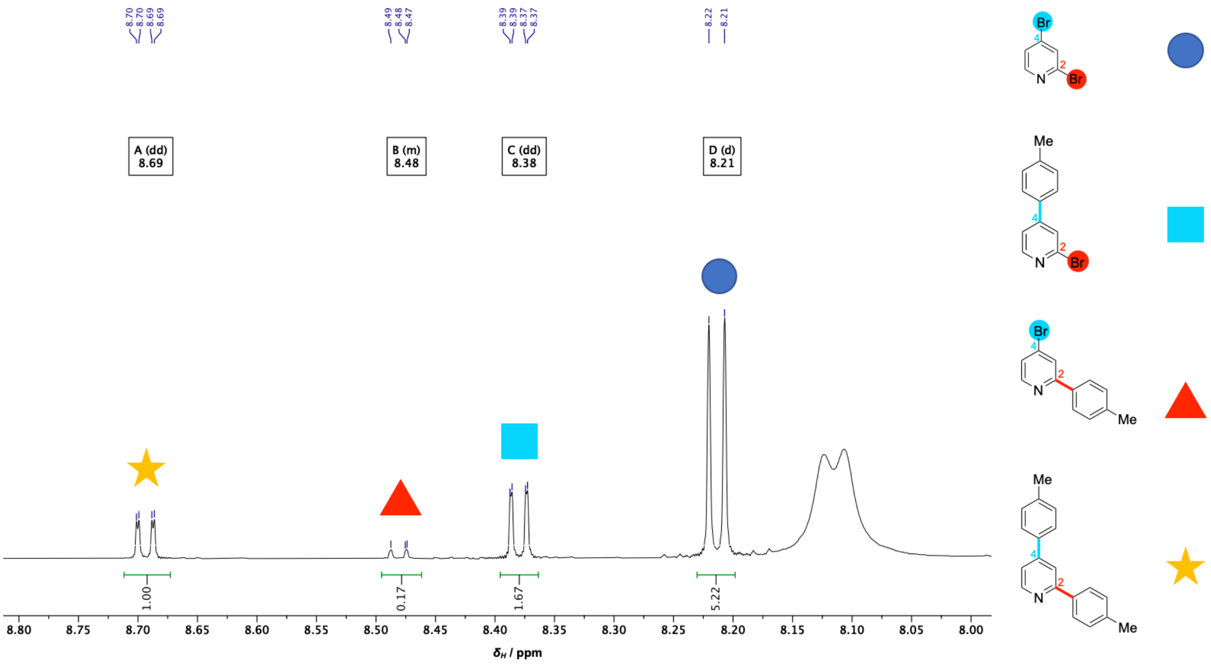

Figure S36. Raw NMR after 6 hours used to estimate the selectivity for the catalyst system on 2,4-dibromopyridine. Peaks were assigned according to the literature.[26]

Table S12. Conversion for the C2, C4, diarylated product and total conversion for the reaction between 2,4-dibromopyridine and 4-tolylboronic acid , **1,** using Pd-gel as the catalyst after 24 hours.

Time / h	Conversion of C2 product / %	Conversion of C4 product / %	Conversion of diarylated product / %	Total Conversion / %
24	2.1	20.7	12.4	35.2

11.4 Resin-Supported Suzuki-Miyaura Cross Coupling Reaction

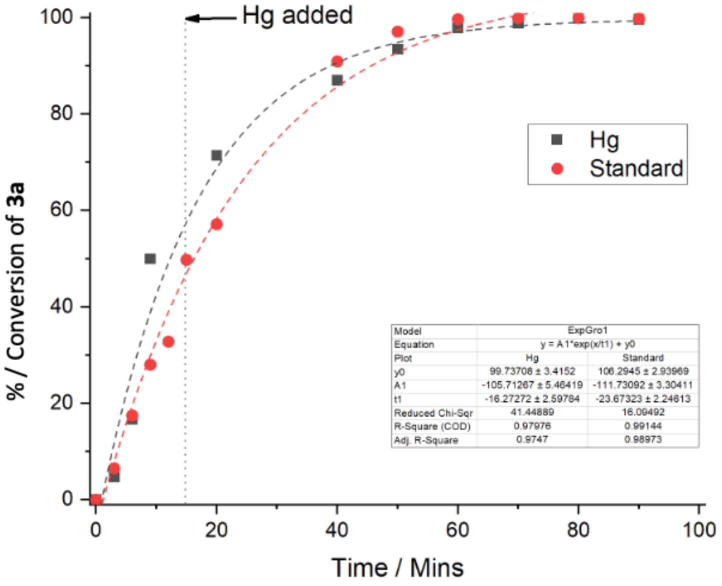

Figure S37. Kinetic profile of the reaction between **3** and **a** under standard conditions, with 0.1 mol% Pd–gel beads with (black line) and without Hg (red line).

11.5 Resin-Supported Suzuki-Miyaura Cross Coupling Reaction

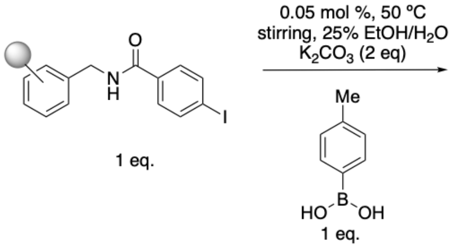

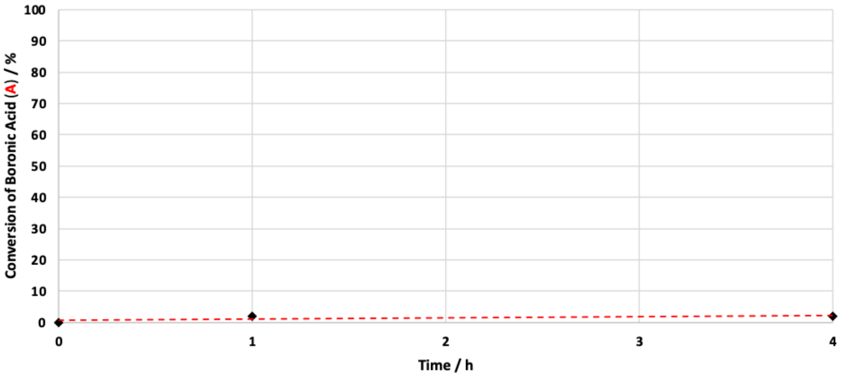

Figure S38. a) Resin-supported aryl iodide used to test activity of leachate towards SMCC reaction. b) Reaction conversions after specified times indicate conversions <5%, demonstrating that the leachate does not contain significant amounts of catalytically-active Pd.

S12 Reaction Set-up

S12.1 Standard Reaction set–up

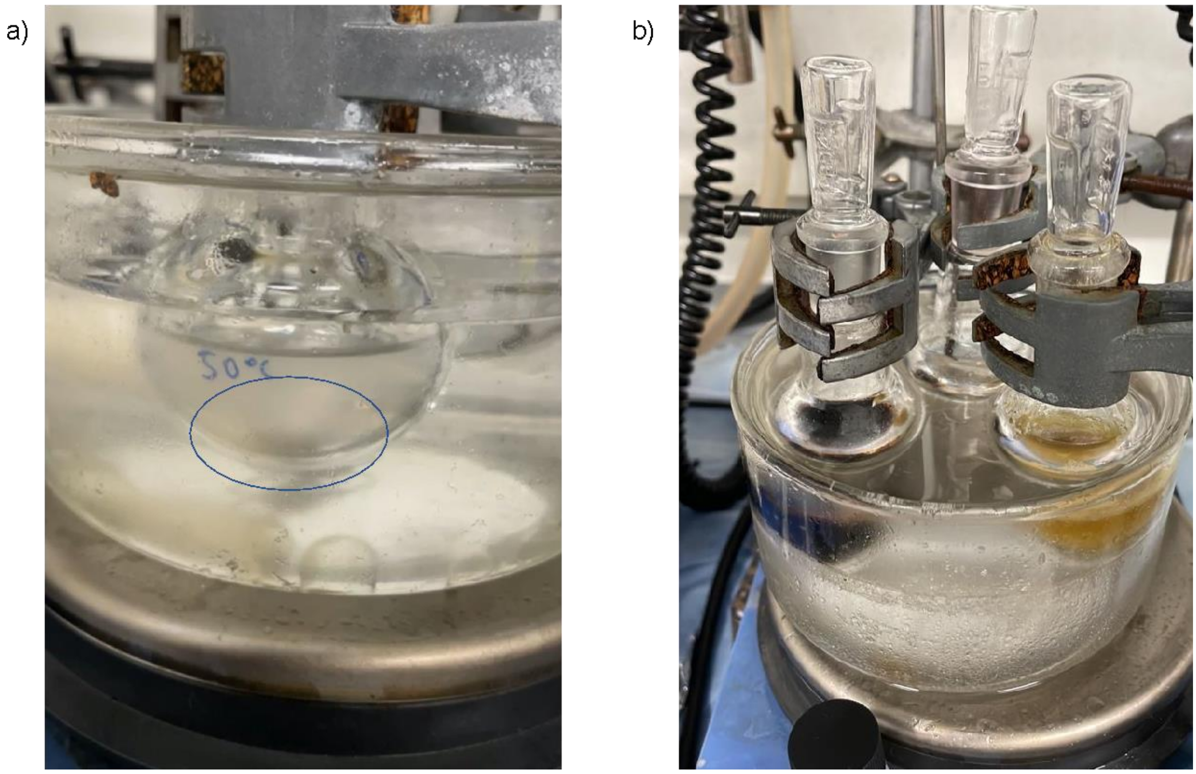

Figure S39. Typical reaction set up. In **a)** the beads and stirrer bar are circled.

S12.2 Gram-Scale Reaction Set-up

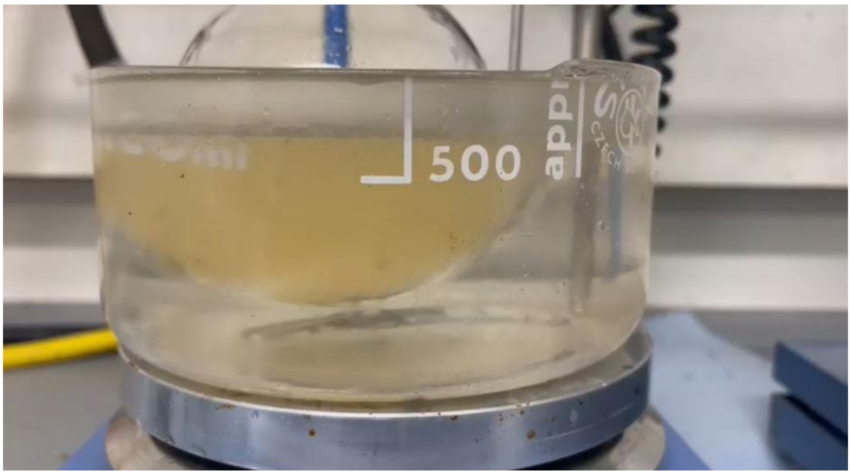

Figure S40. Gram-scale reaction set-up for the synthesis of **1a**.

S13 Characterisation of Suzuki-Miyaura Cross Coupling Products

1a

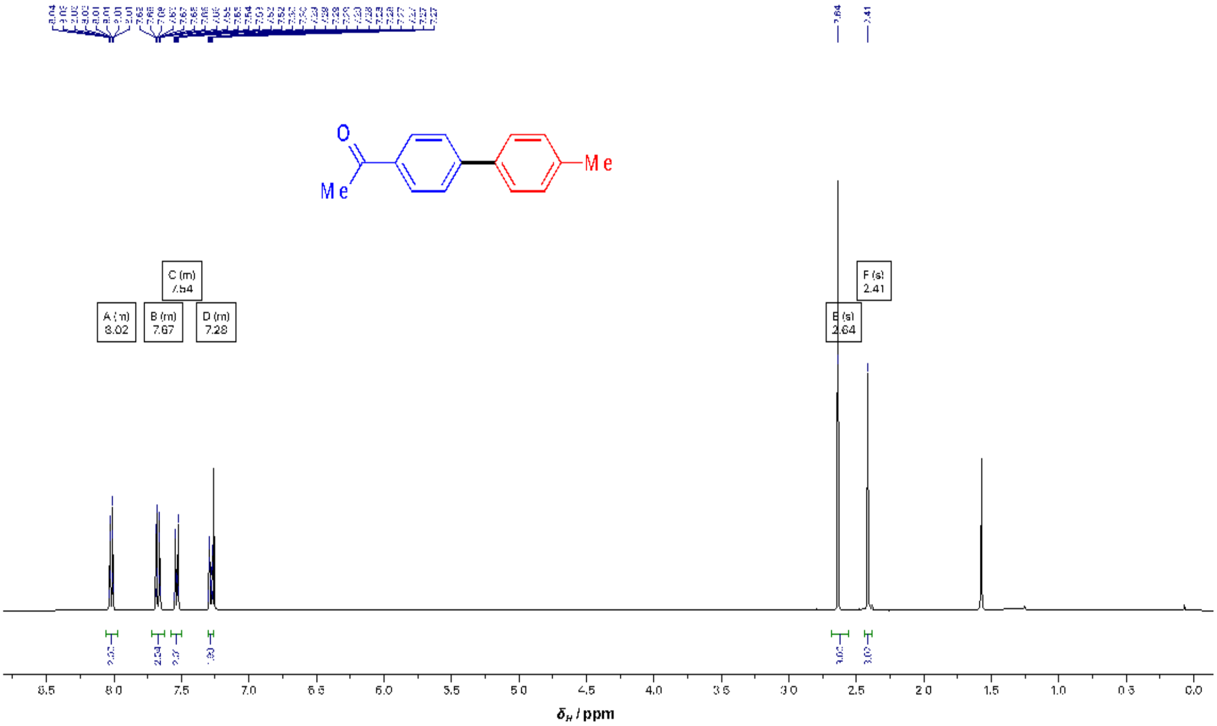

Figure S41. 400 MHz ^{1}H NMR of **1a** in $CDCl_3$

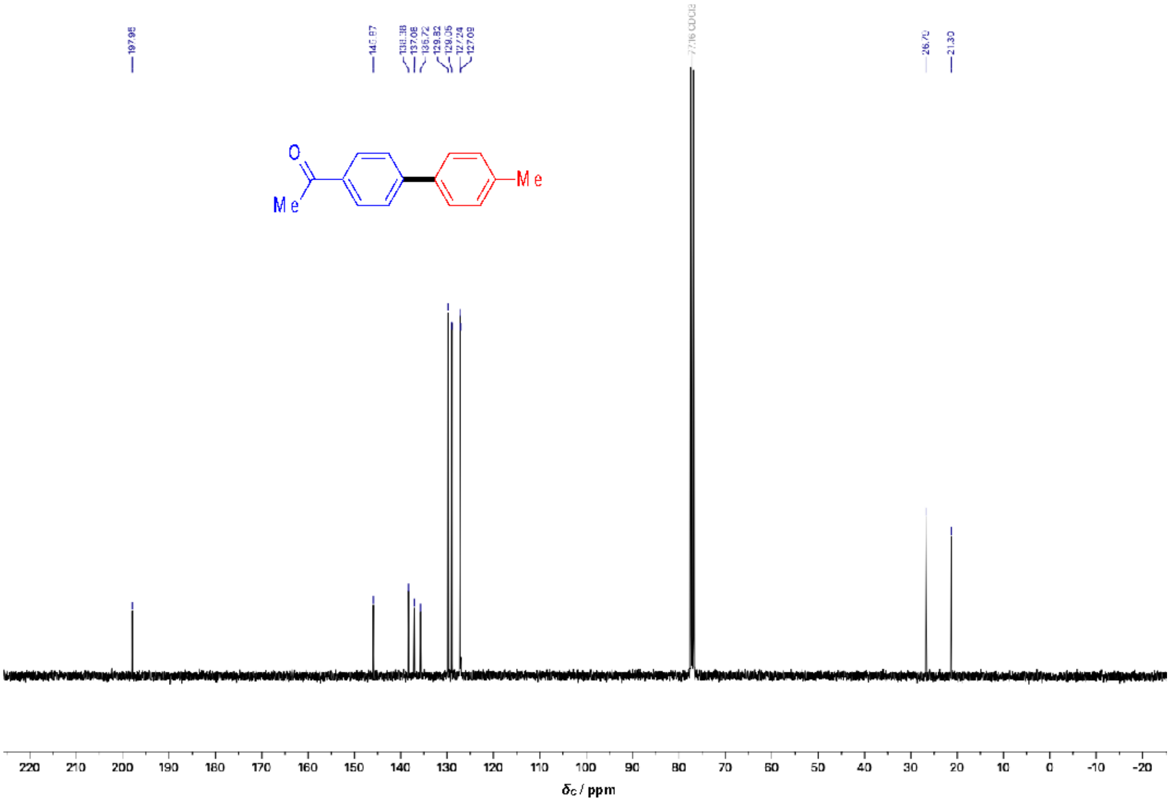

Figure S42. 101 MHz ^{13}C NMR of **1a** in $CDCl_3$

1b

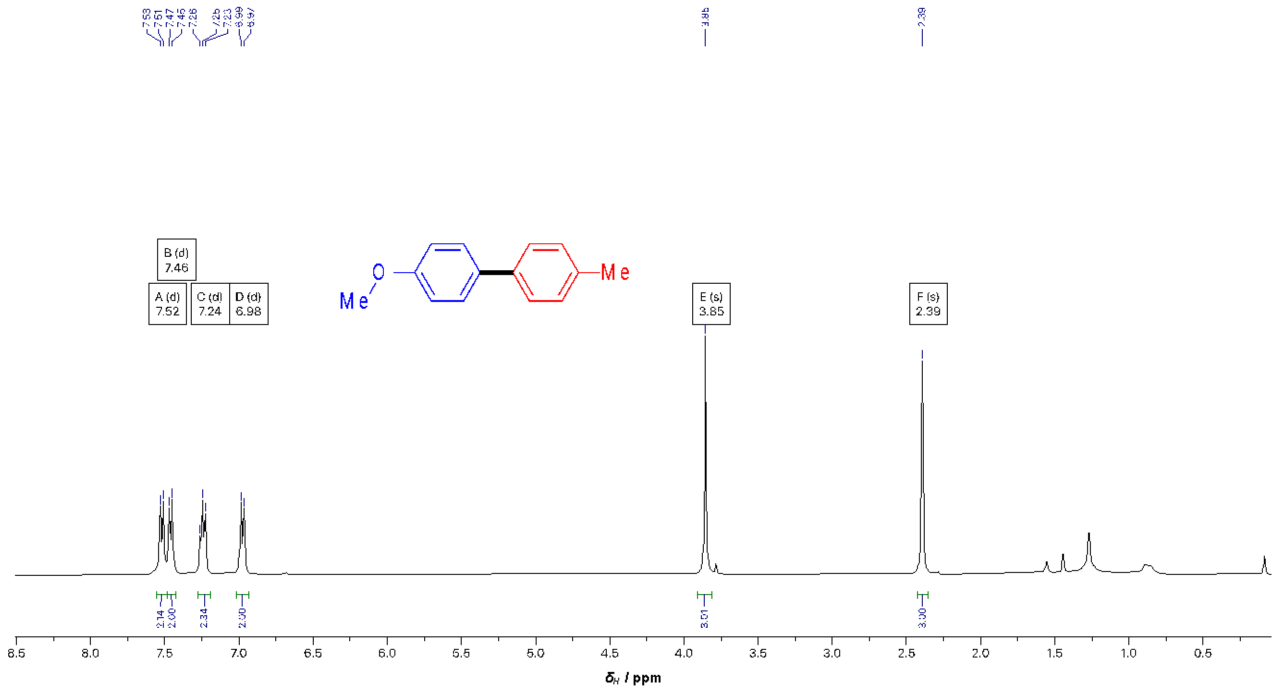

Figure S43. 400 MHz ^{1}H NMR of **1b** in $CDCl_3$

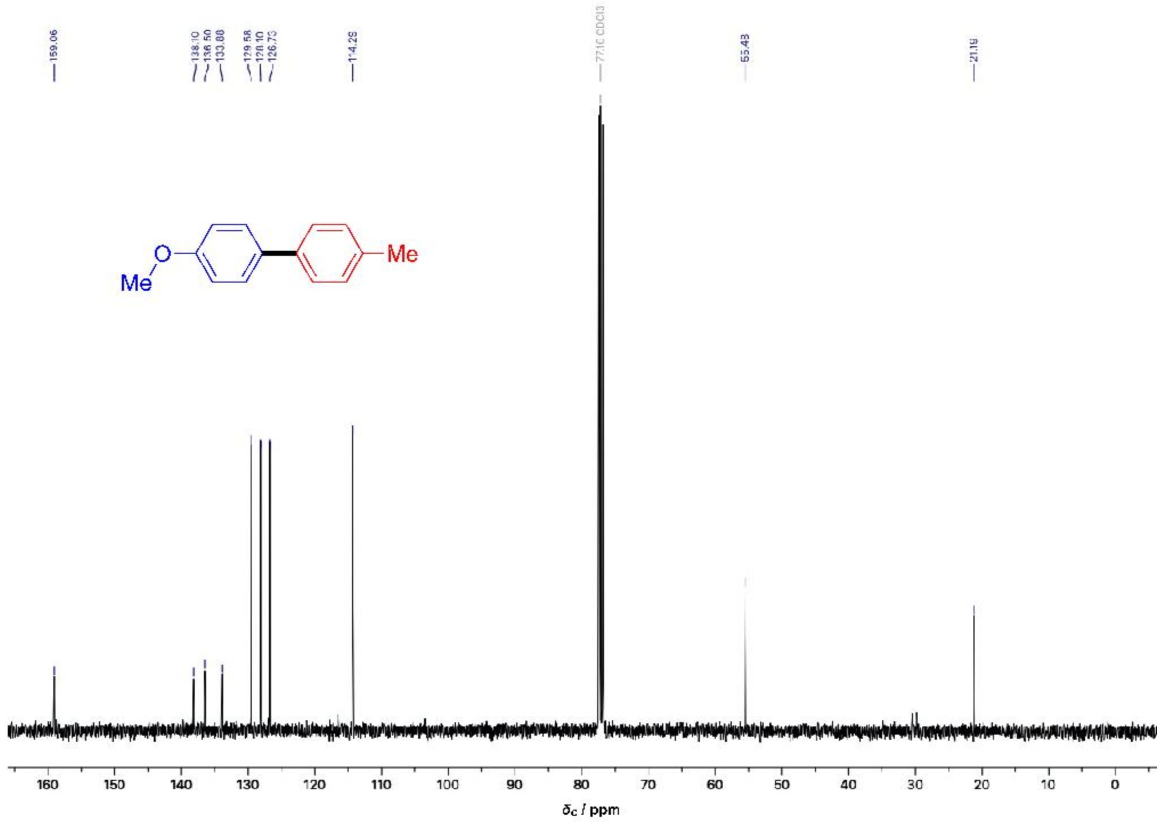

Figure S44. 101 MHz ^{13}C NMR of **1b** in $CDCl_3$

1c

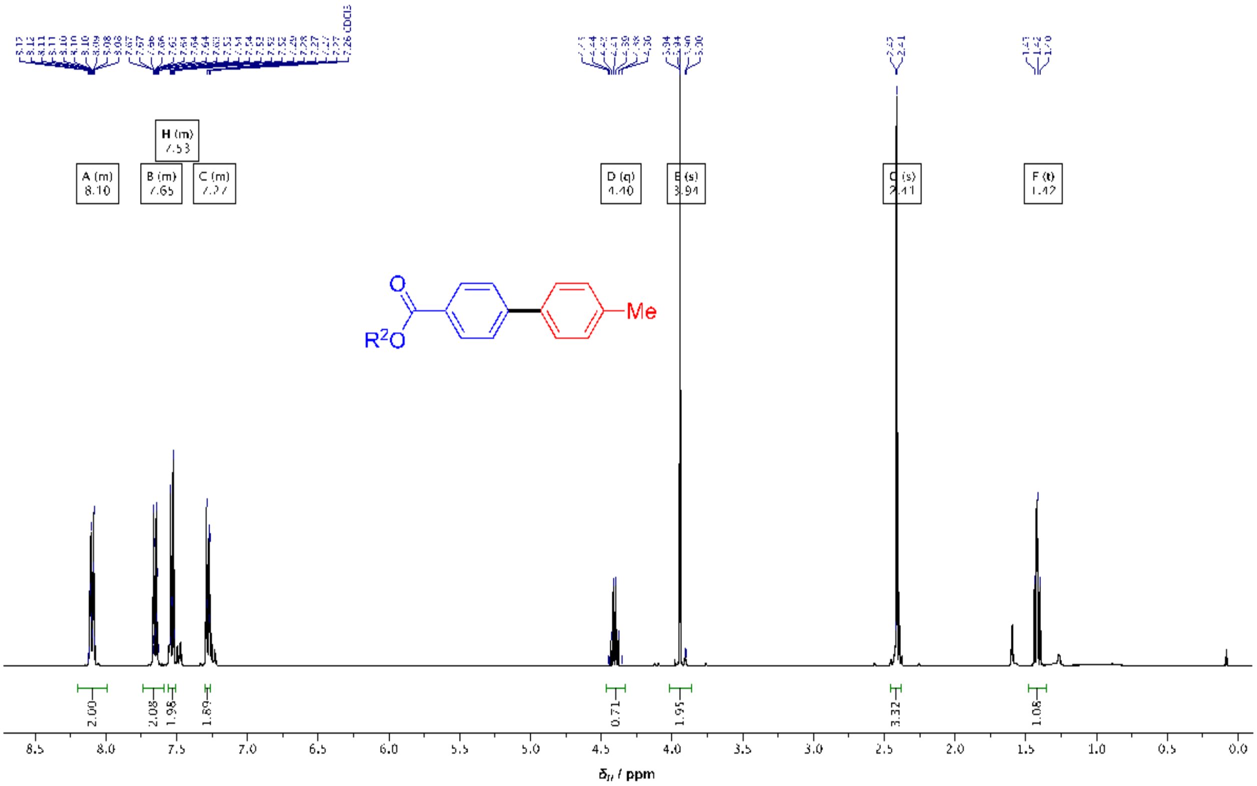

Figure S45. 400 MHz ^{1}H NMR of **1c** in $CDCl_3$

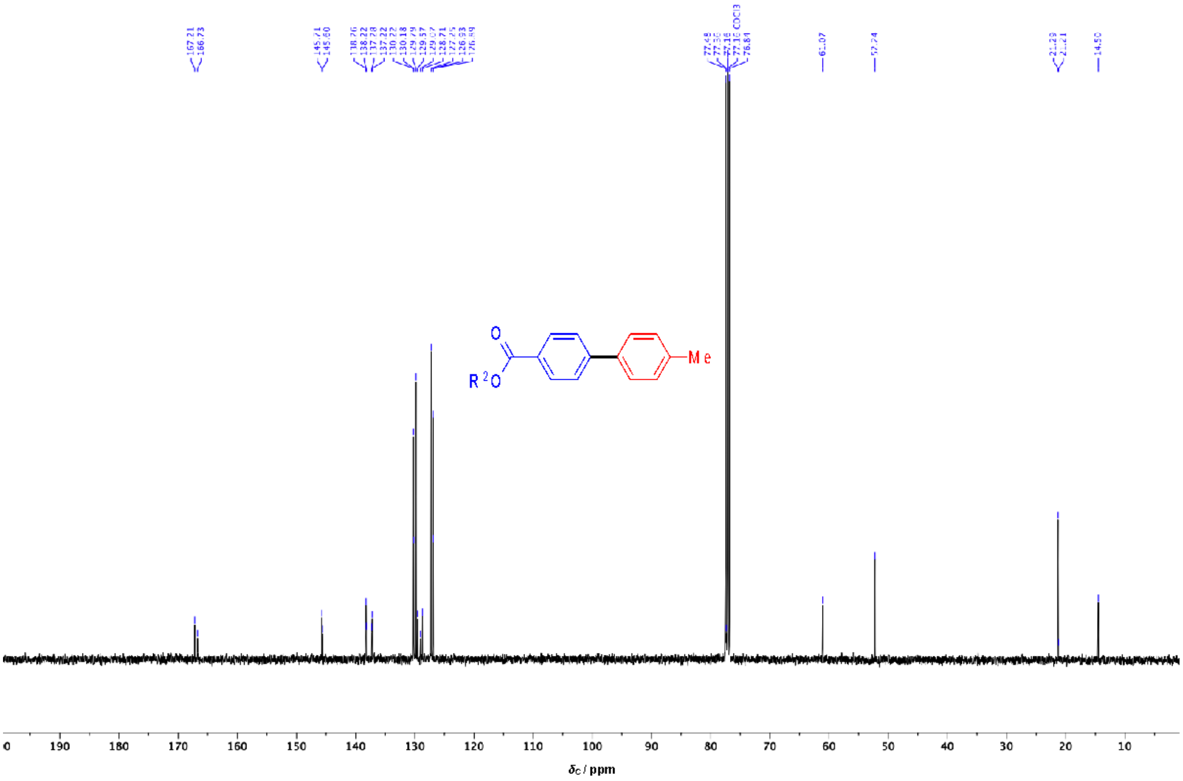

Figure S46. 101 MHz ^{13}C NMR of **1c** in $CDCl_3$

1d

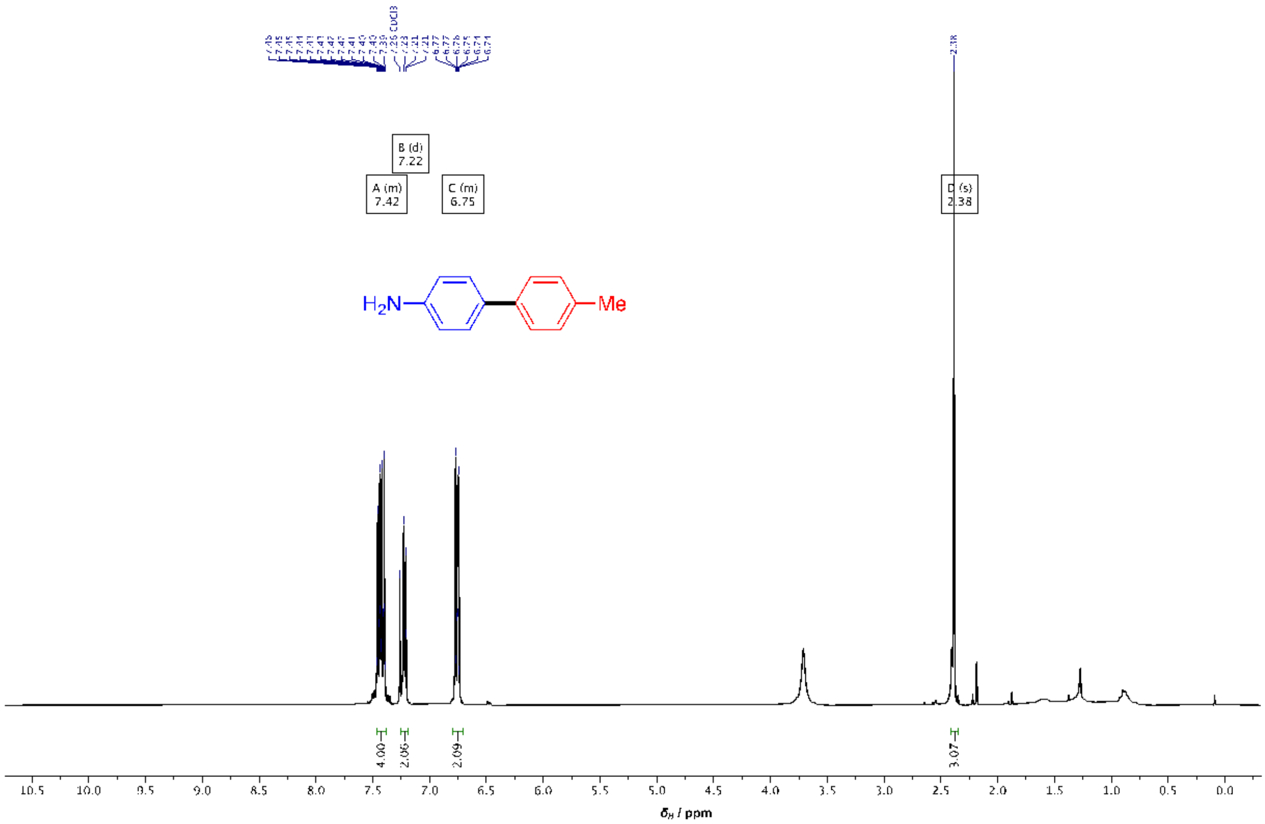

Figure S47. 400 MHz ^{1}H NMR of **1d** in $CDCl_3$

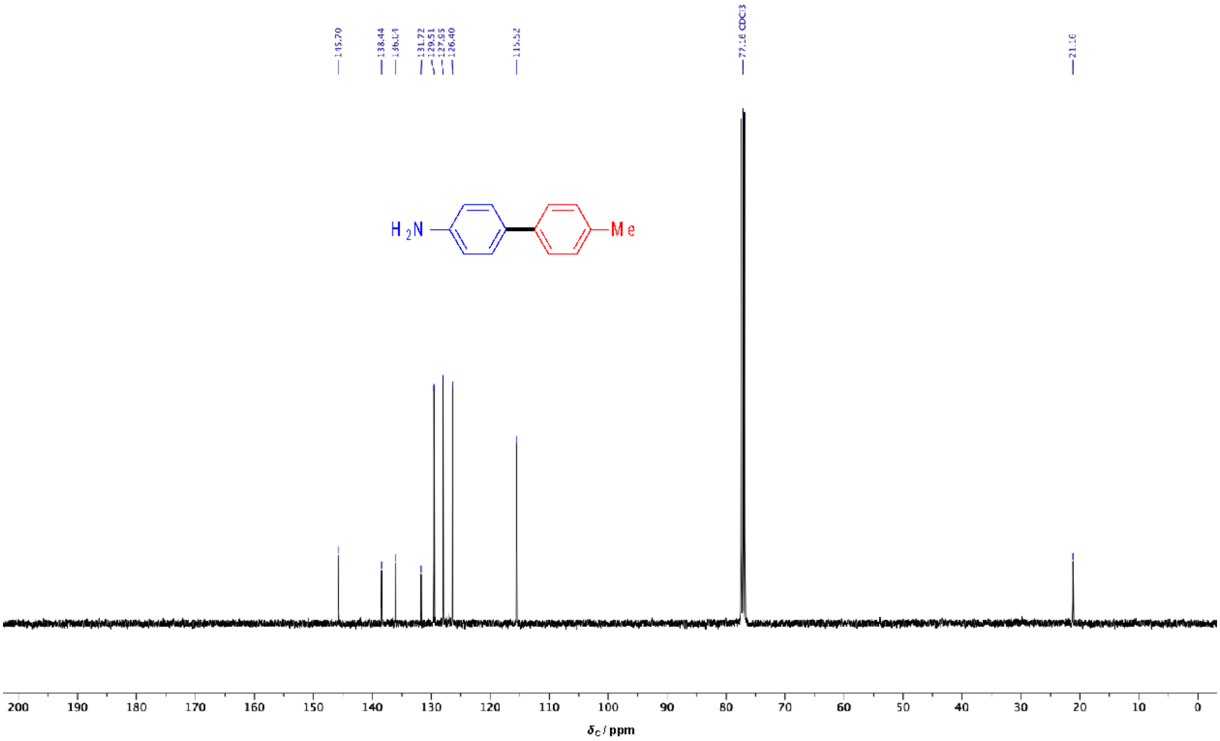

Figure S48. 101 MHz ^{13}C NMR of **1d** in $CDCl_3$

1e

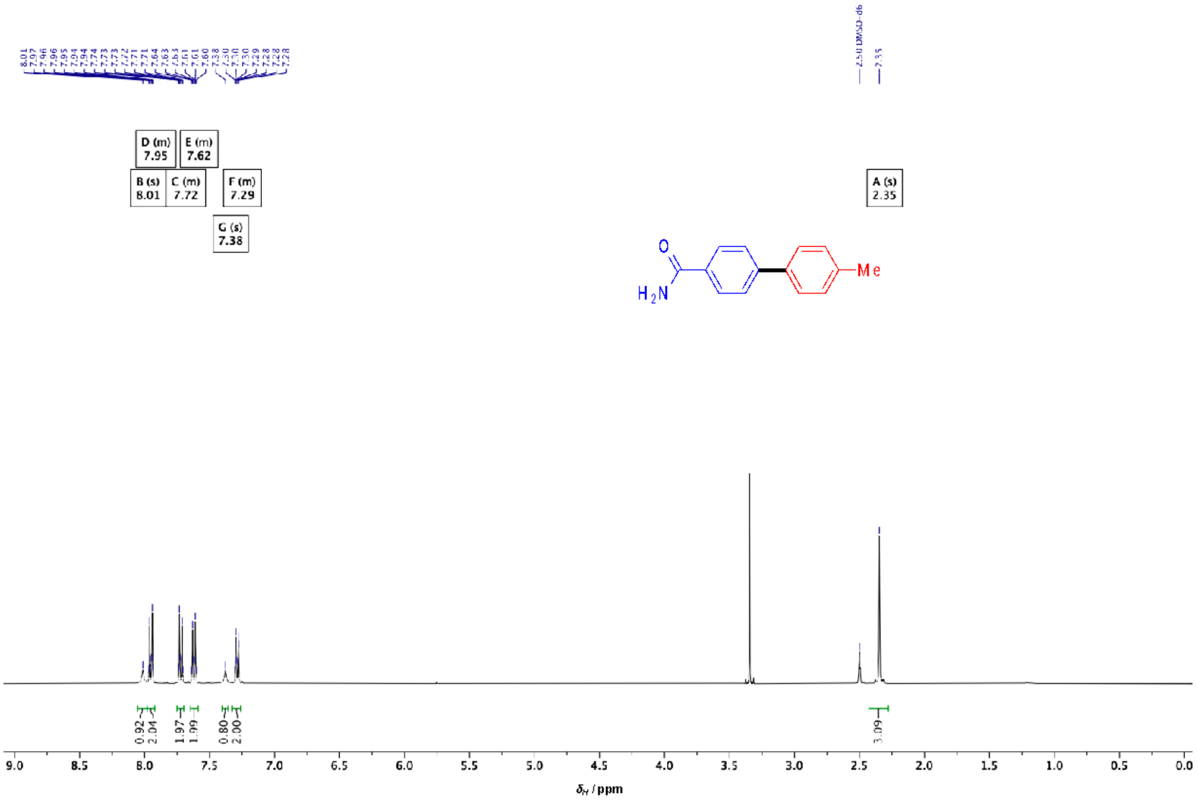

Figure S49. 400 MHz ^{1}H NMR of **1e** in DMSO-d_6

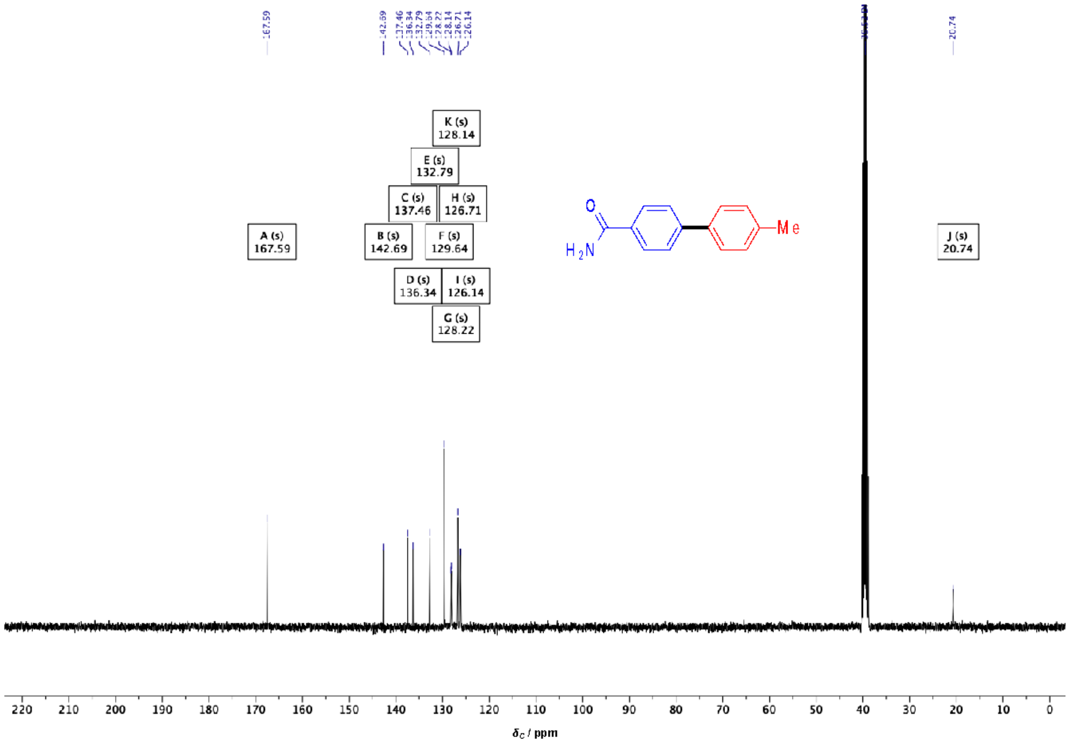

Figure S50. 101 MHz ^{13}C NMR of **1e** in DMSO-d_6

1f

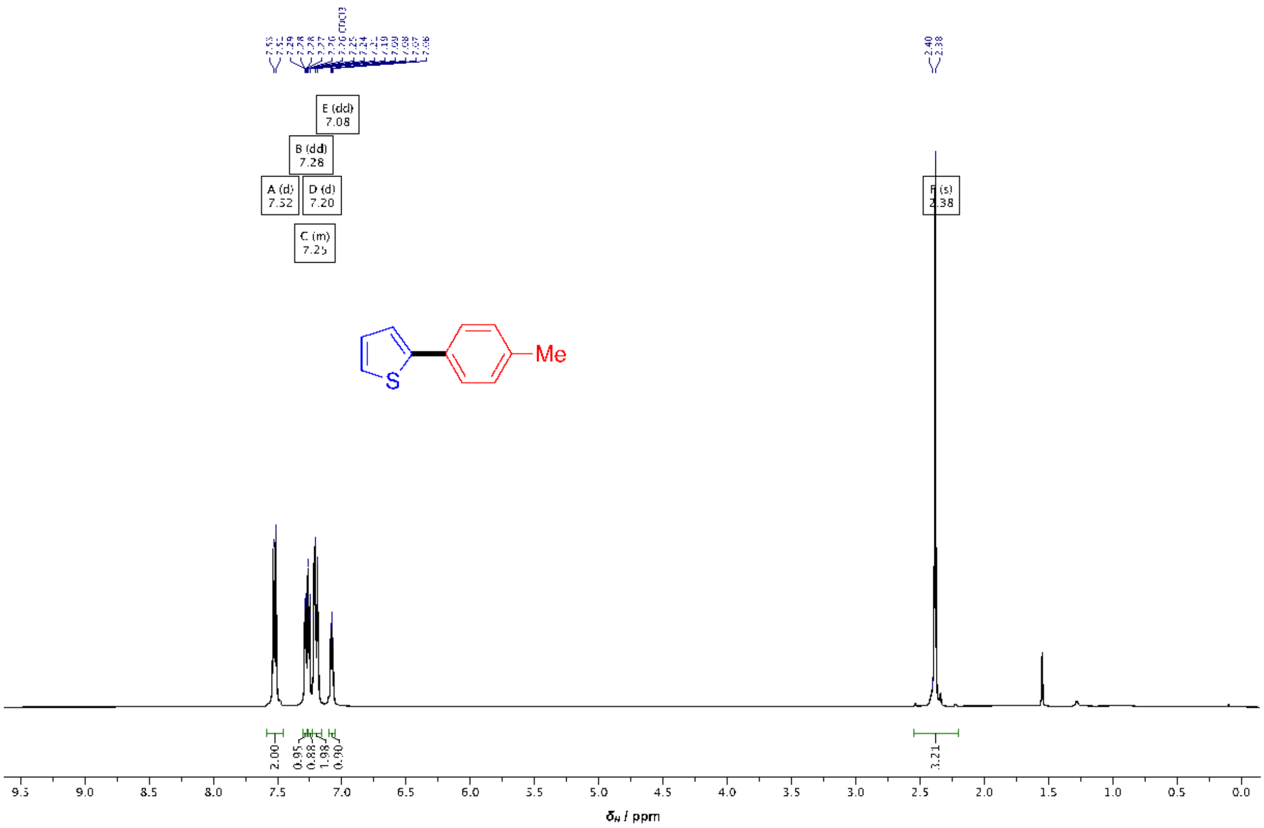

Figure S51. 400 MHz ^{1}H NMR of **1f** in $CDCl_3$

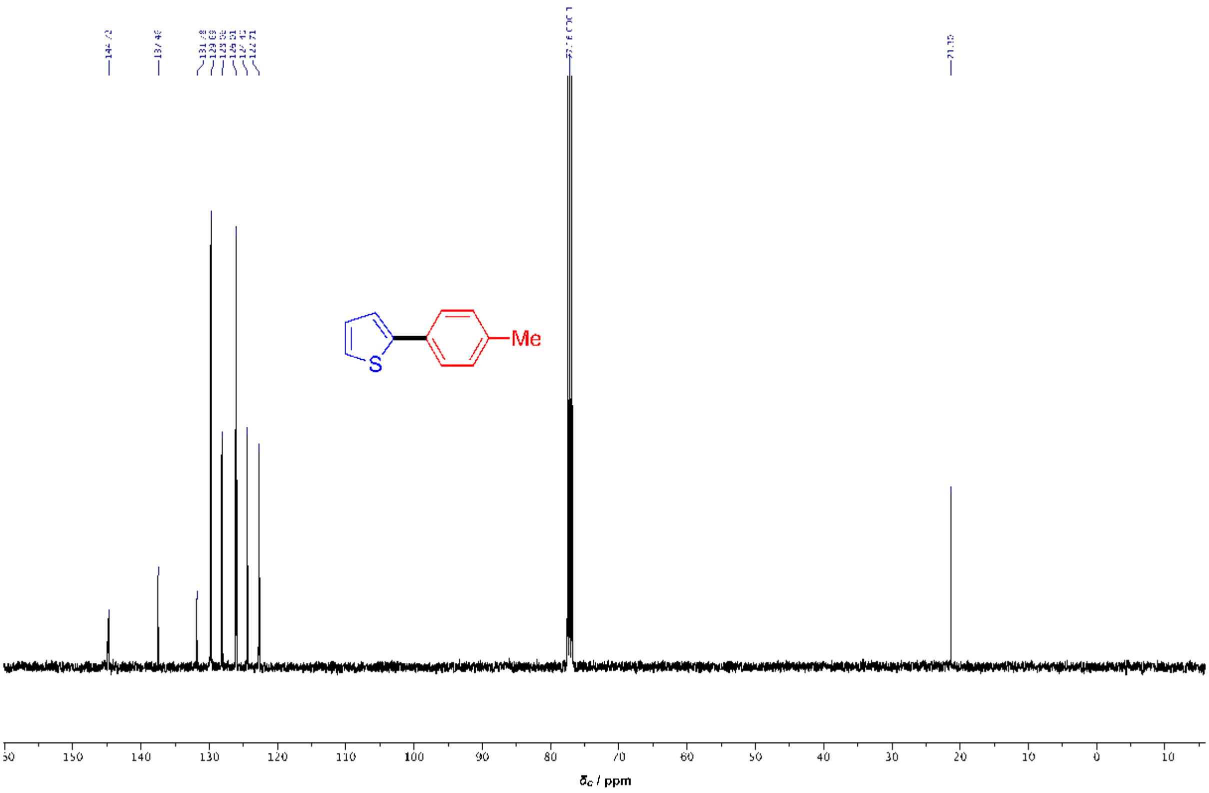

Figure S52. 101 MHz ^{13}C NMR of **1f** in $CDCl_3$

1g

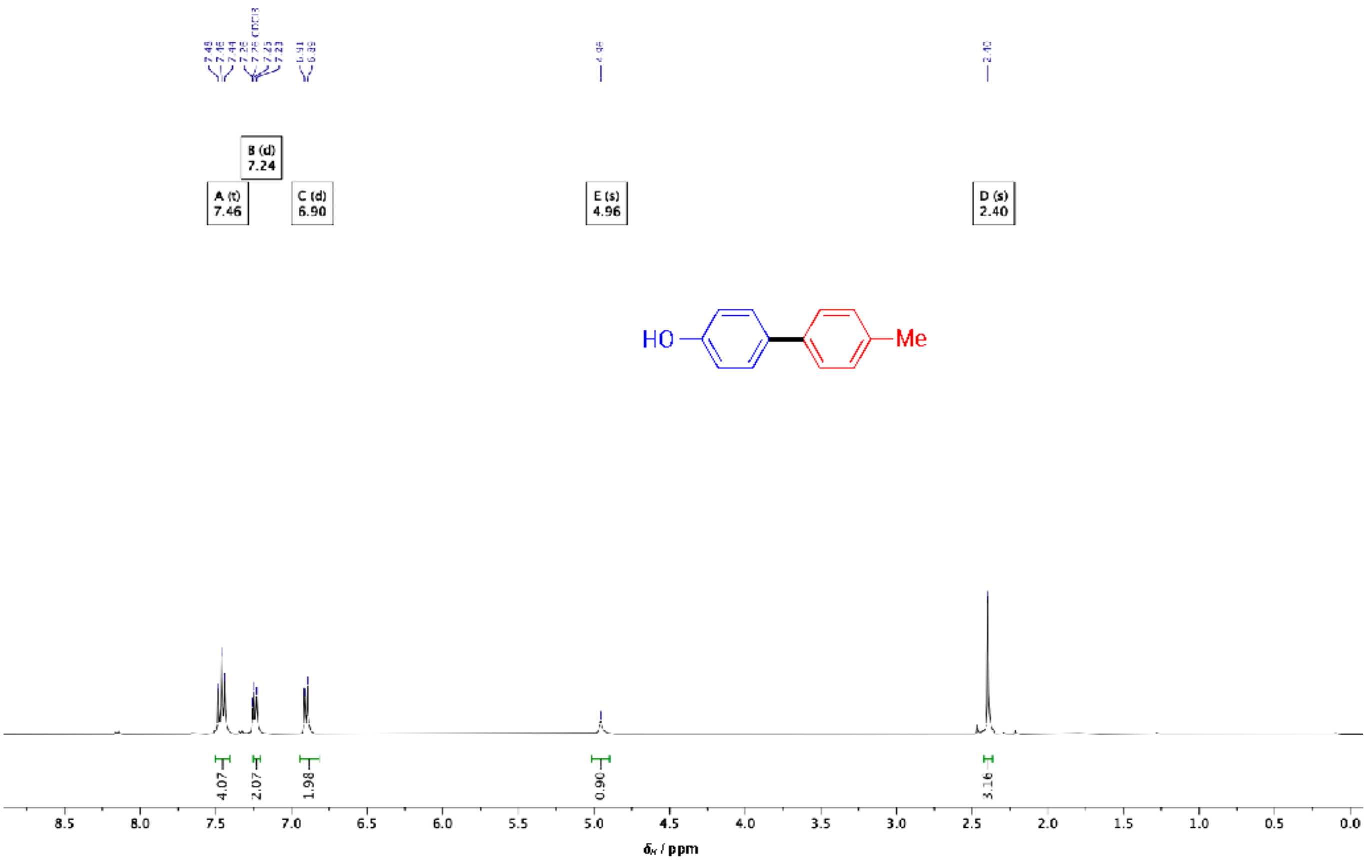

Figure S53. 400 MHz ^{1}H NMR of **1g** in $CDCl_3$

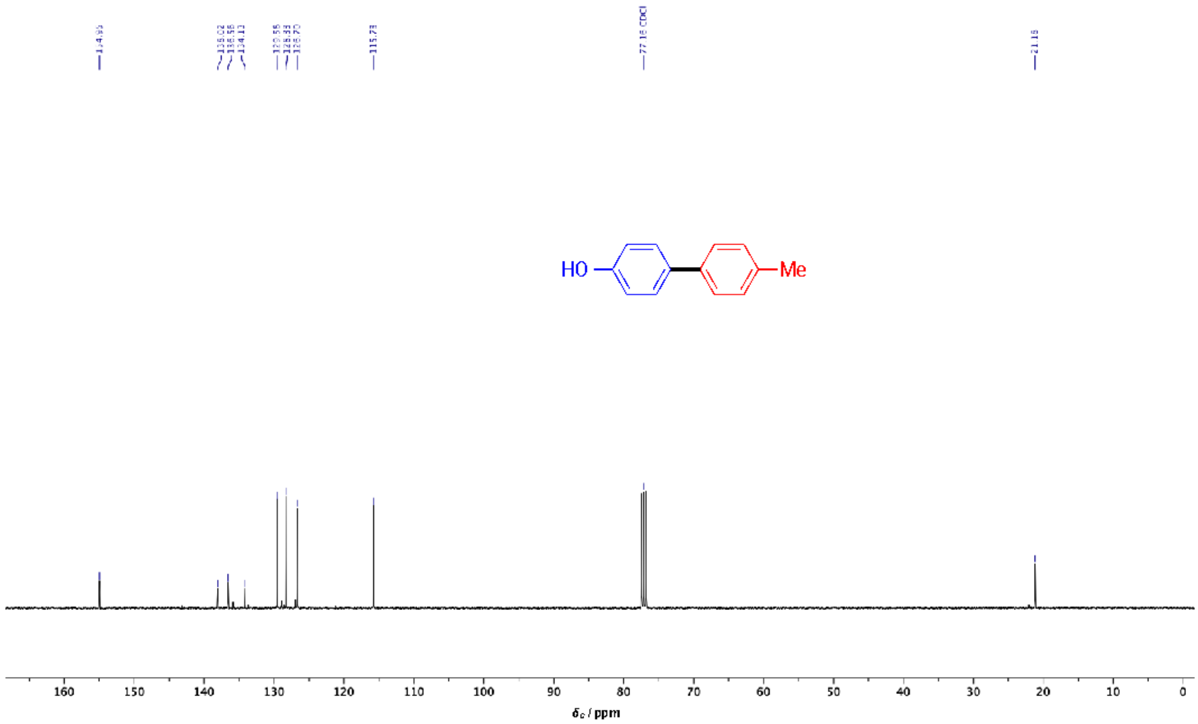

Figure S54. 101 MHz ^{13}C NMR of **1g** in $CDCl_3$

1h

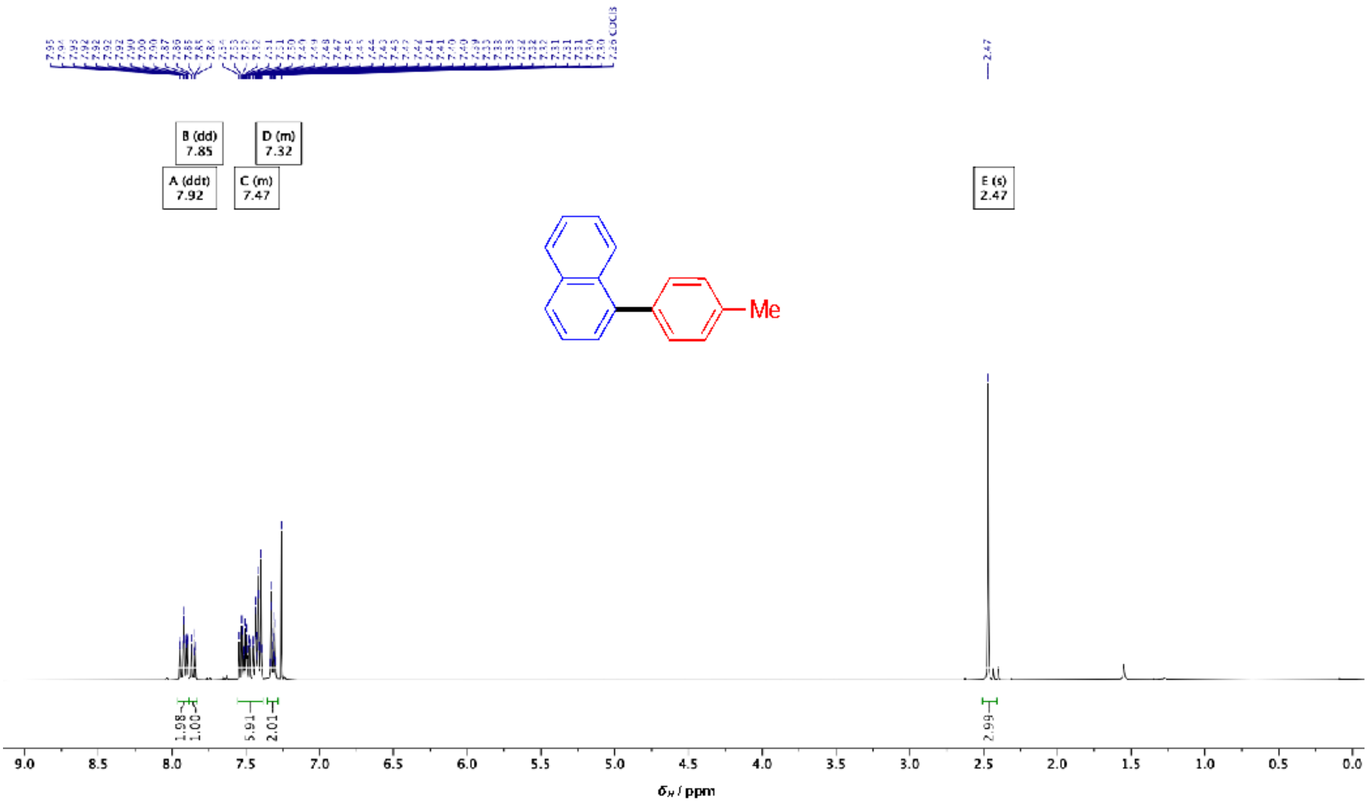

Figure S55. 400 MHz ^{1}H NMR of **1h** in $CDCl_3$

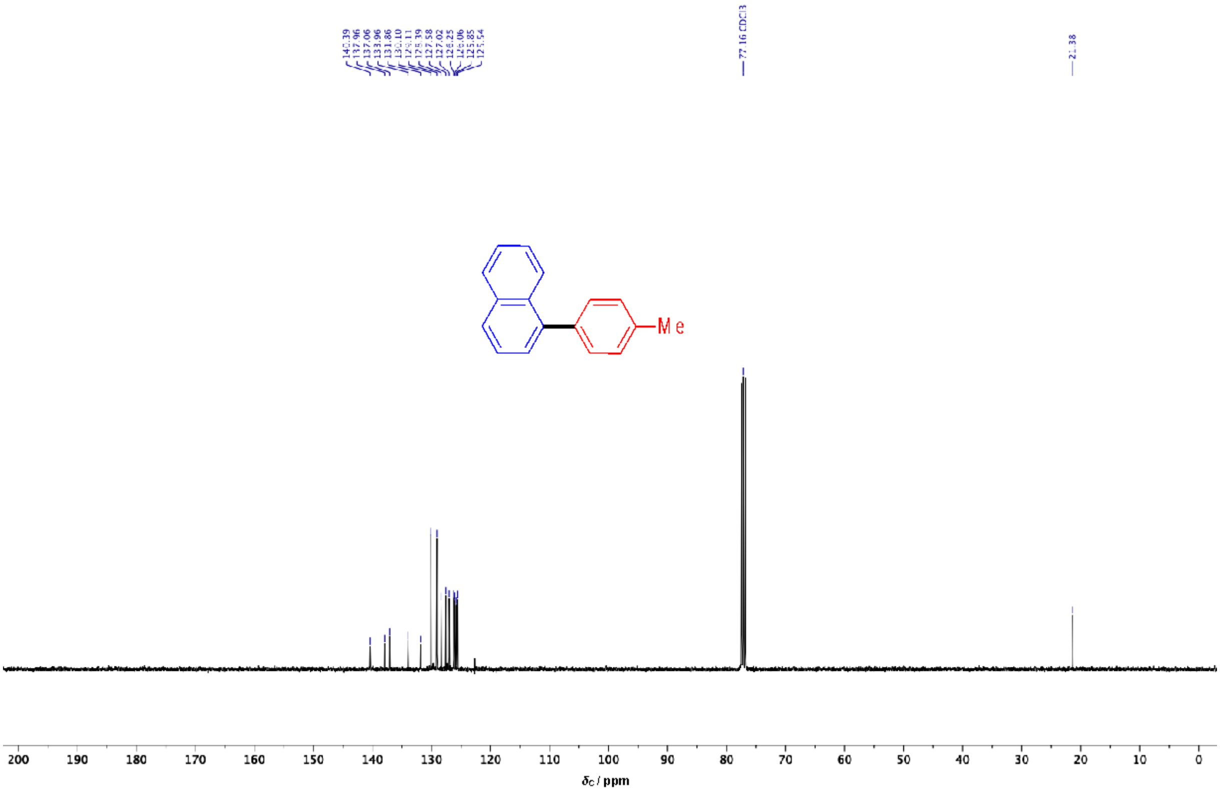

Figure S56. 101 MHz ^{13}C NMR of **1g** in $CDCl_3$

1i

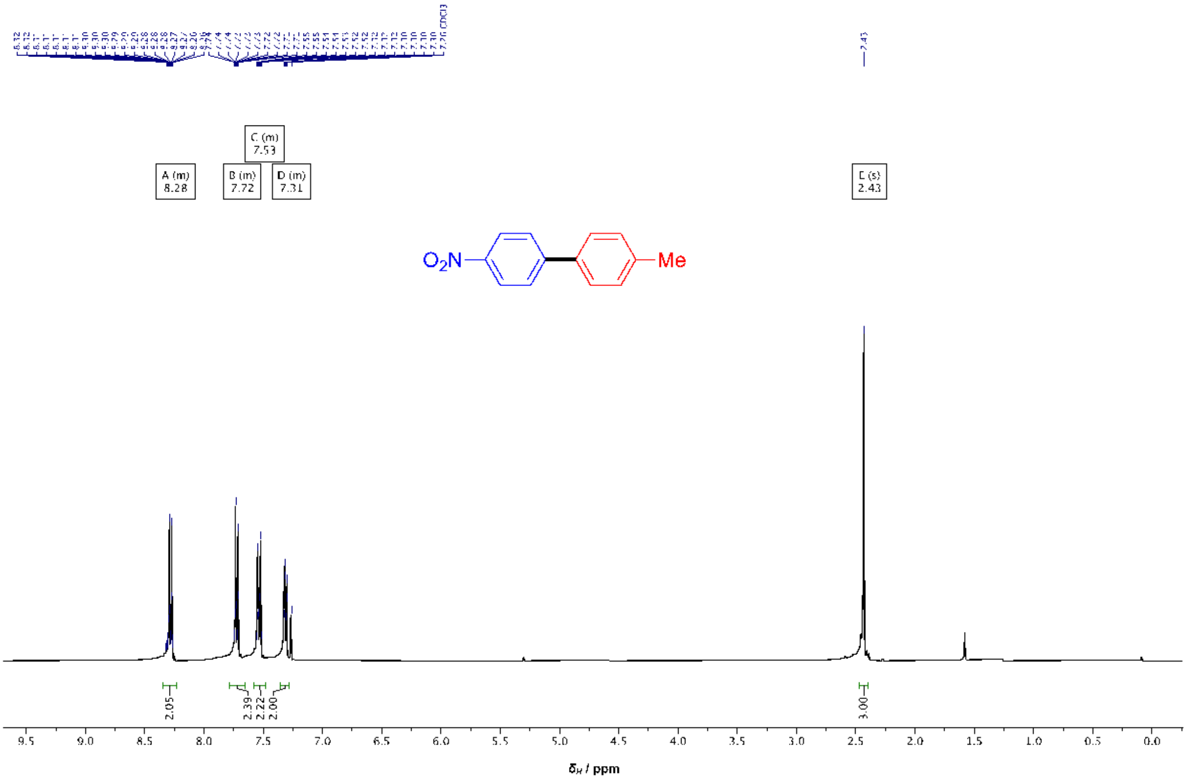

Figure S57. 400 MHz ^{1}H NMR of **1i** in $CDCl_3$

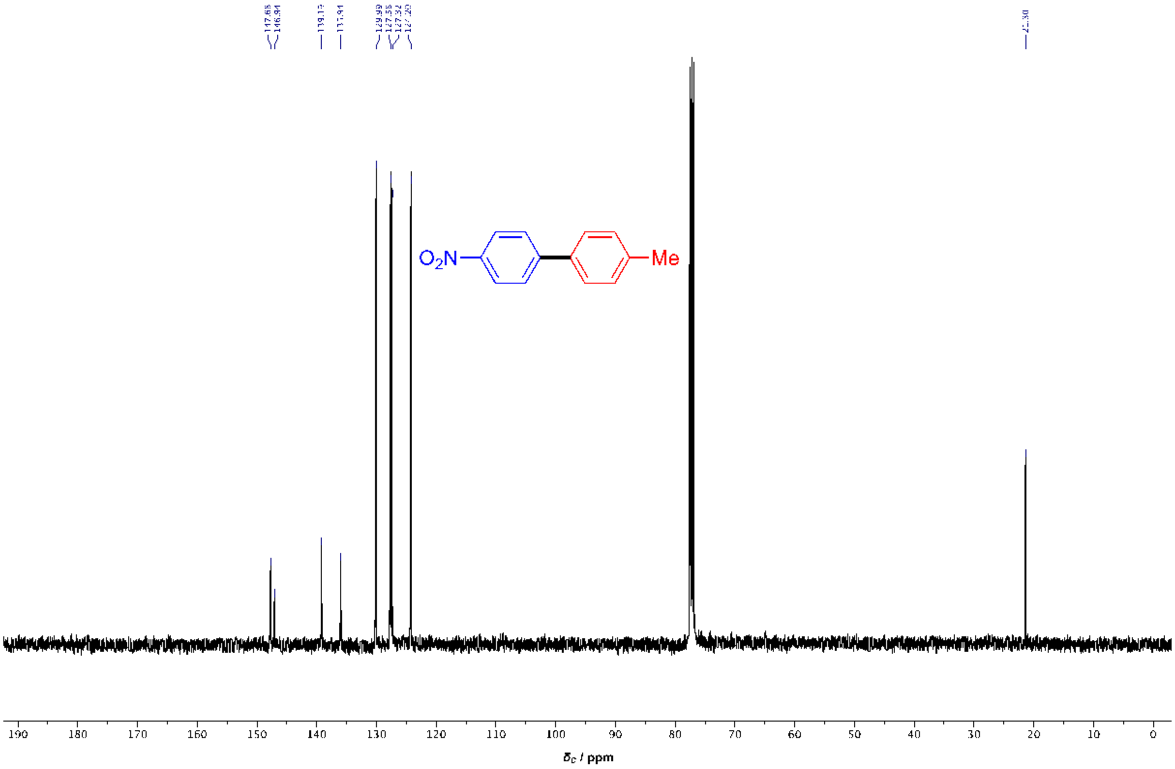

Figure S58. 101 MHz ^{13}C NMR of **1g** in $CDCl_3$

1j

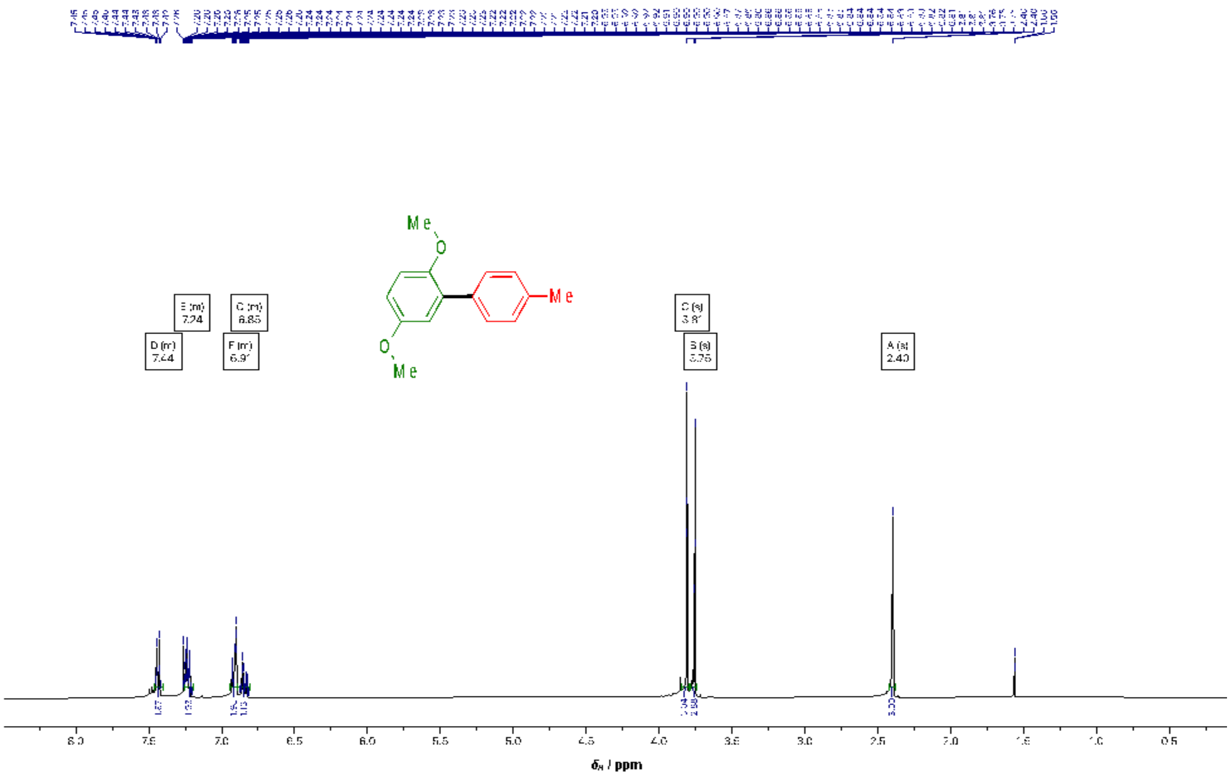

Figure S59. 400 MHz ^{1}H NMR of **1j** in $CDCl_3$

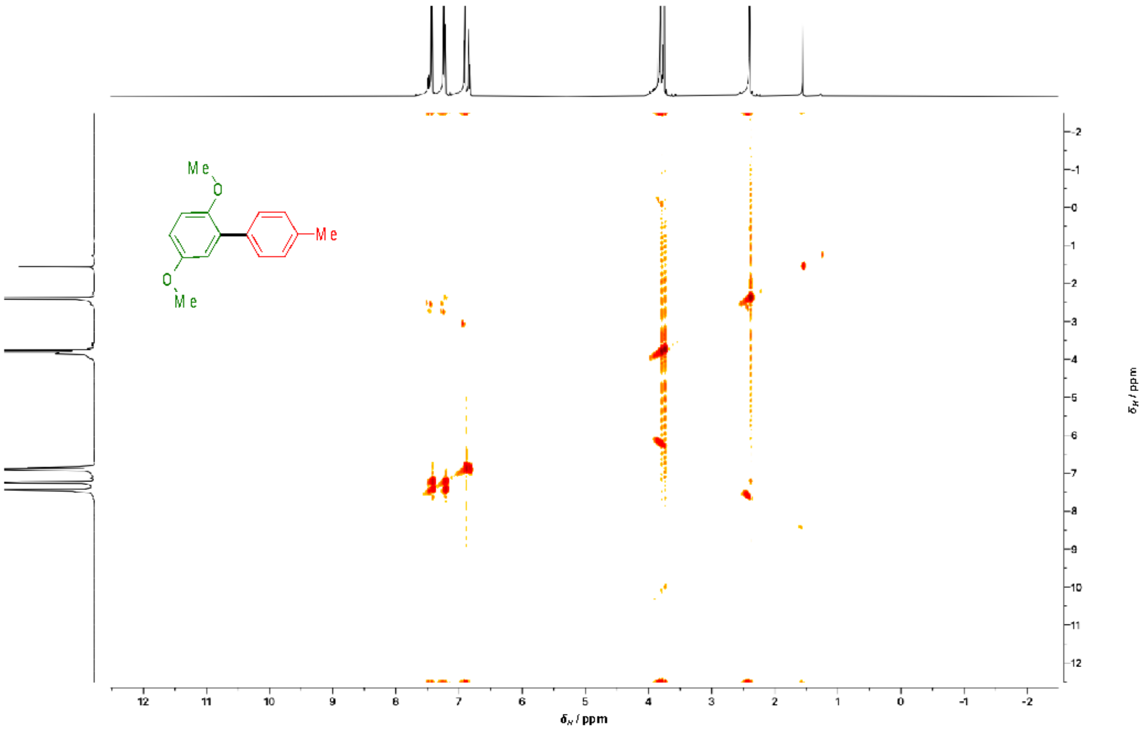

Figure S60. COSY spectra of **1j** in $CDCl_3$

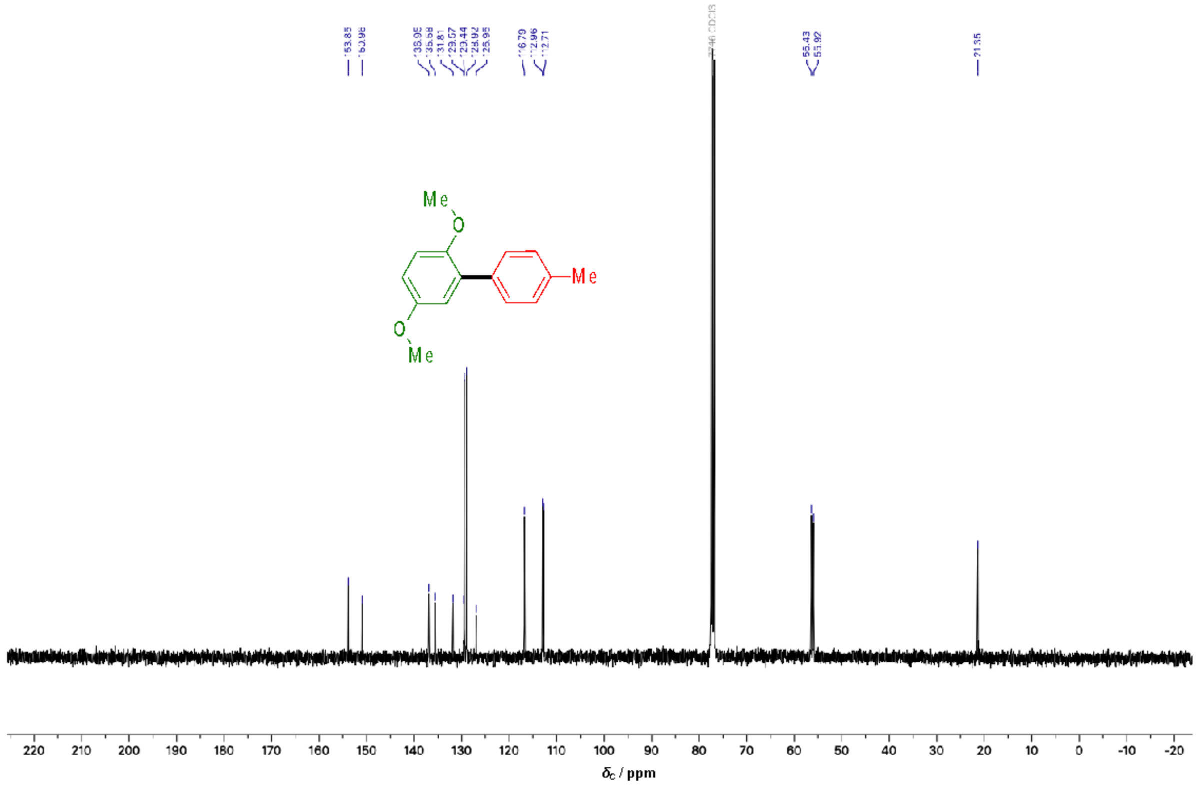

Figure S61. 101 MHz ^{13}C NMR of **1g** in $CDCl_3$

1k

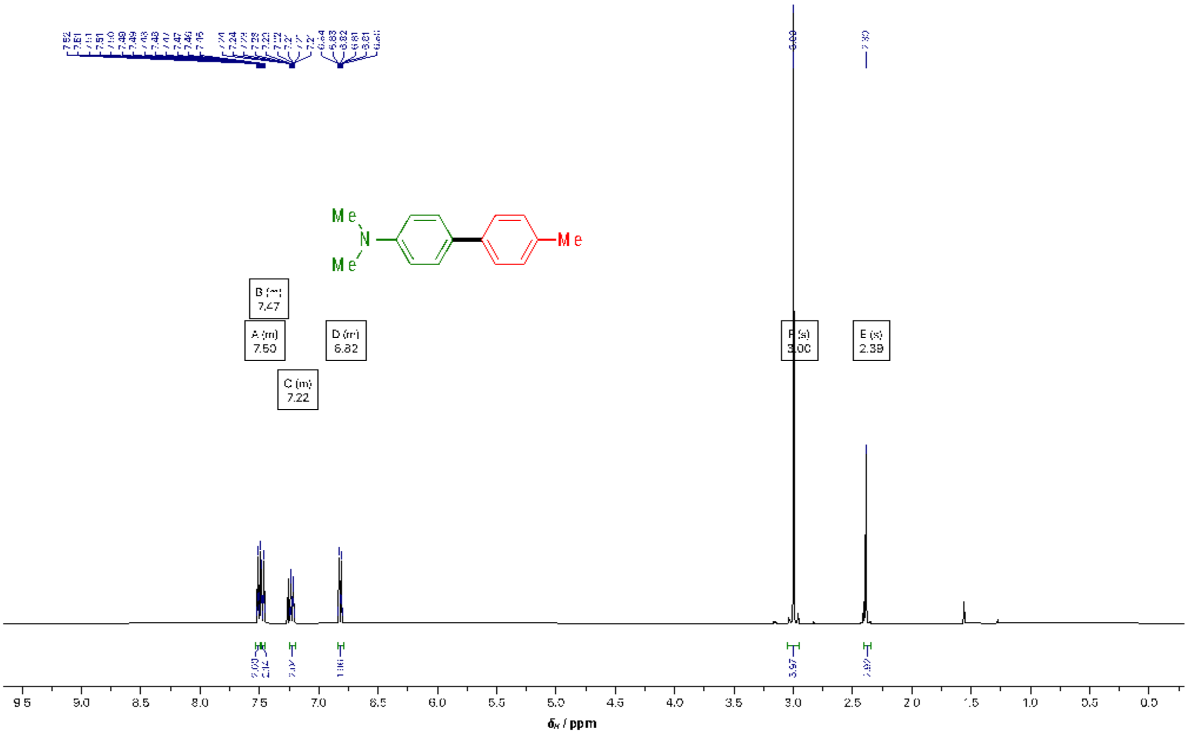

Figure S62. 400 MHz ^{1}H NMR of **1k** in $CDCl_3$

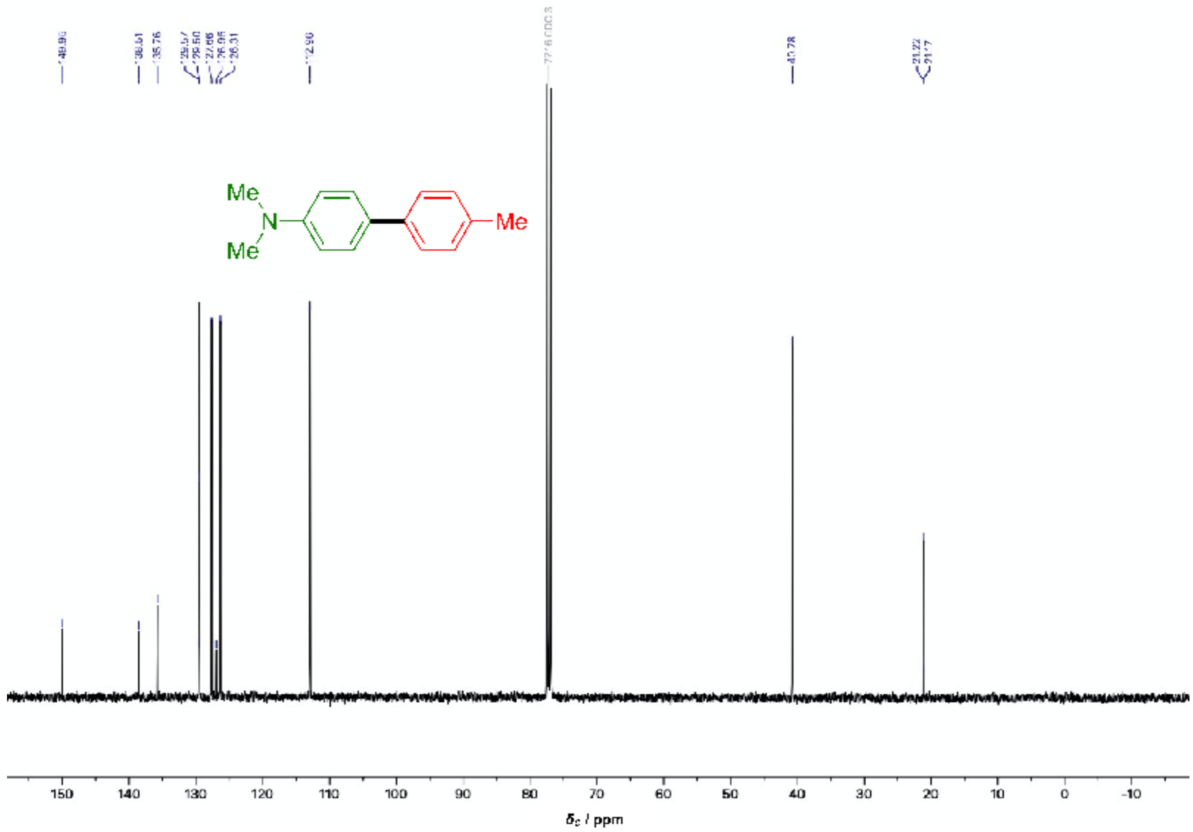

Figure S63. 101 MHz ^{13}C NMR of **1k** in $CDCl_3$

1l

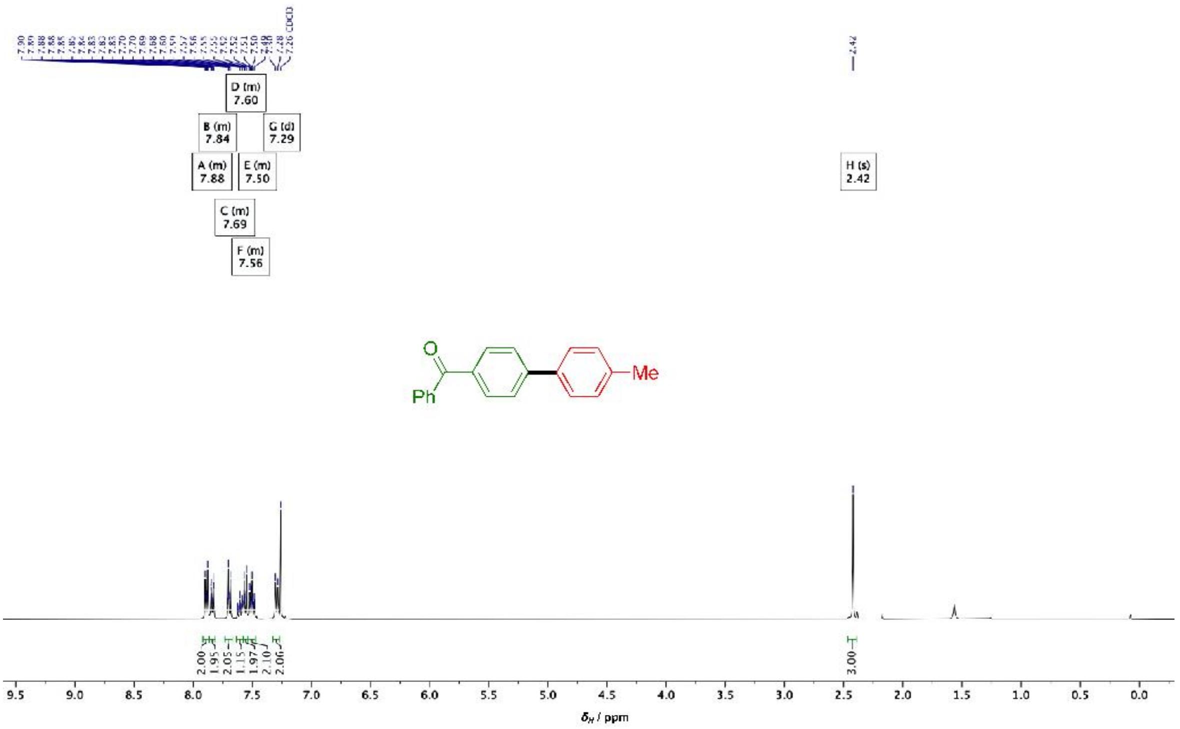

Figure S64. 400 MHz ^{1}H NMR of **1l** in $CDCl_3$

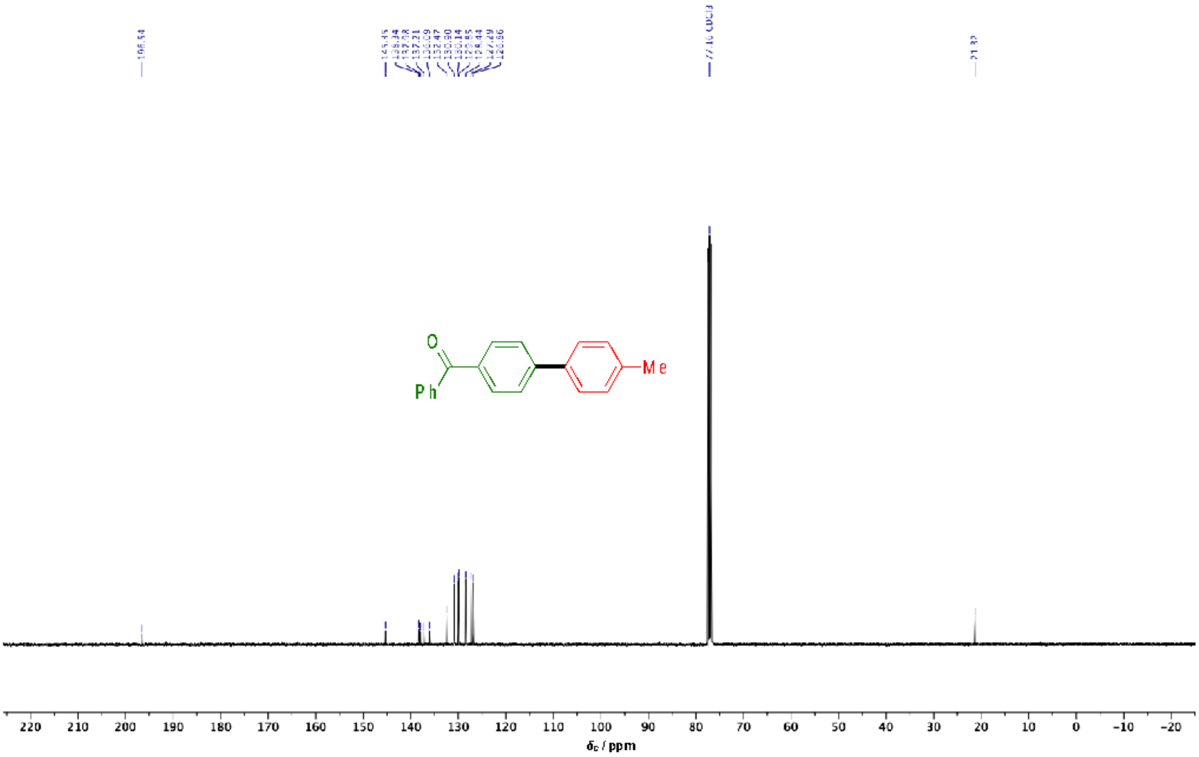

Figure S65. 101 MHz ^{13}C NMR of **1l** in $CDCl_3$

2a

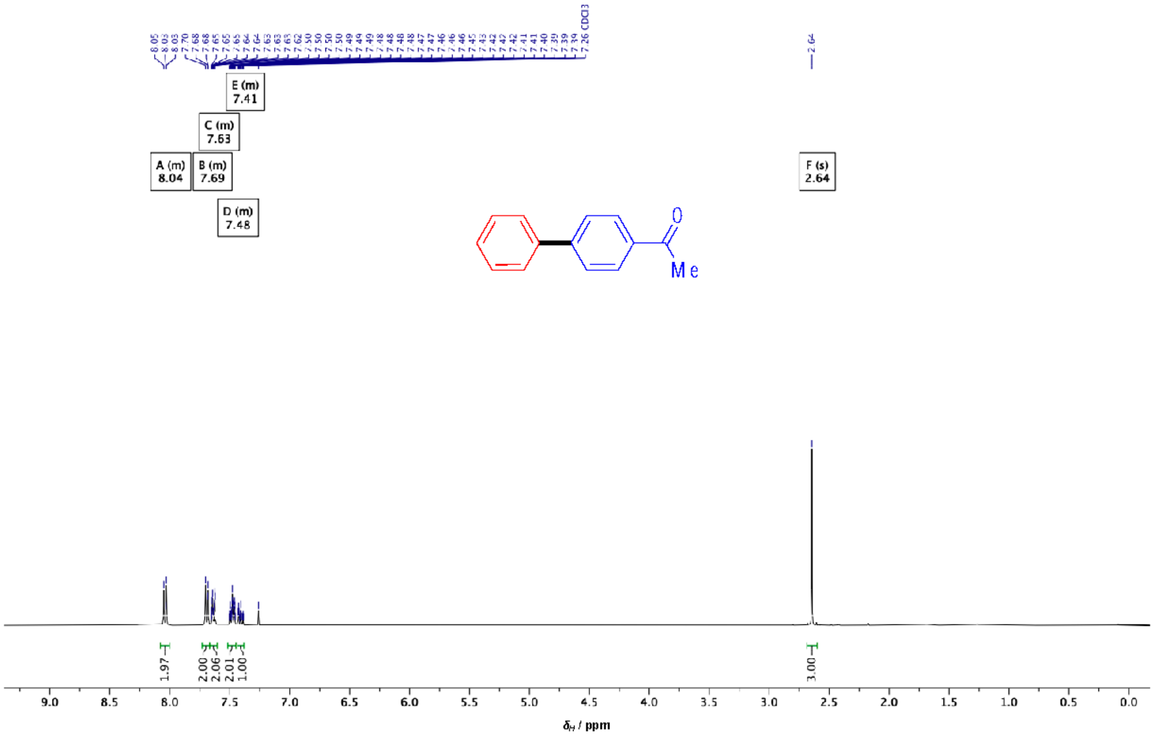

Figure S66. 400 MHz ^{1}H NMR of **2a** in $CDCl_3$

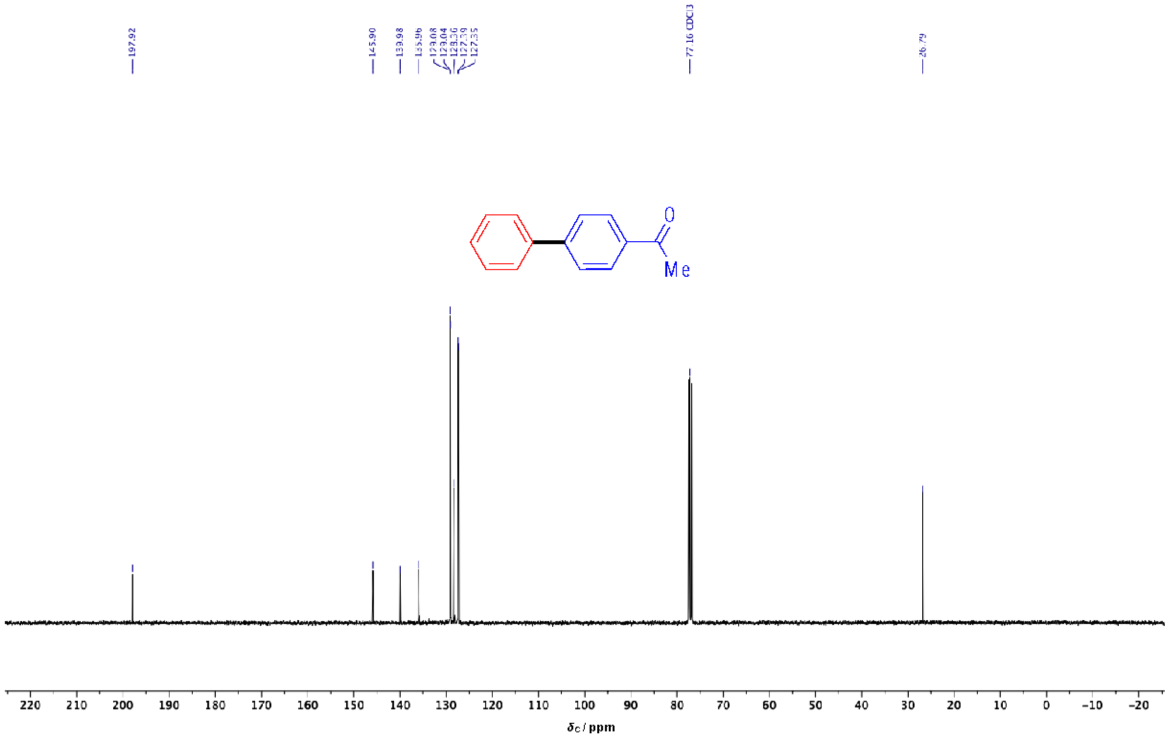

Figure S67. 101 MHz ^{13}C NMR of **2a** in $CDCl_3$

3a

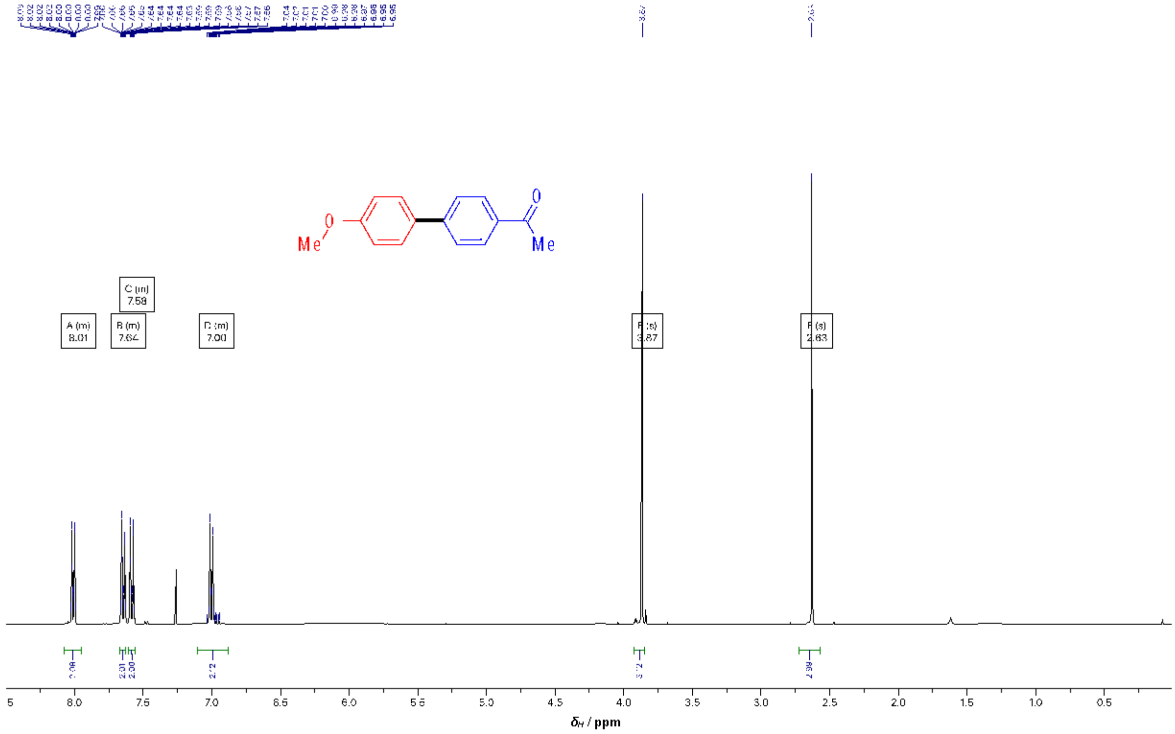

Figure S68. 400 MHz ^{1}H NMR of **3a** in $CDCl_3$

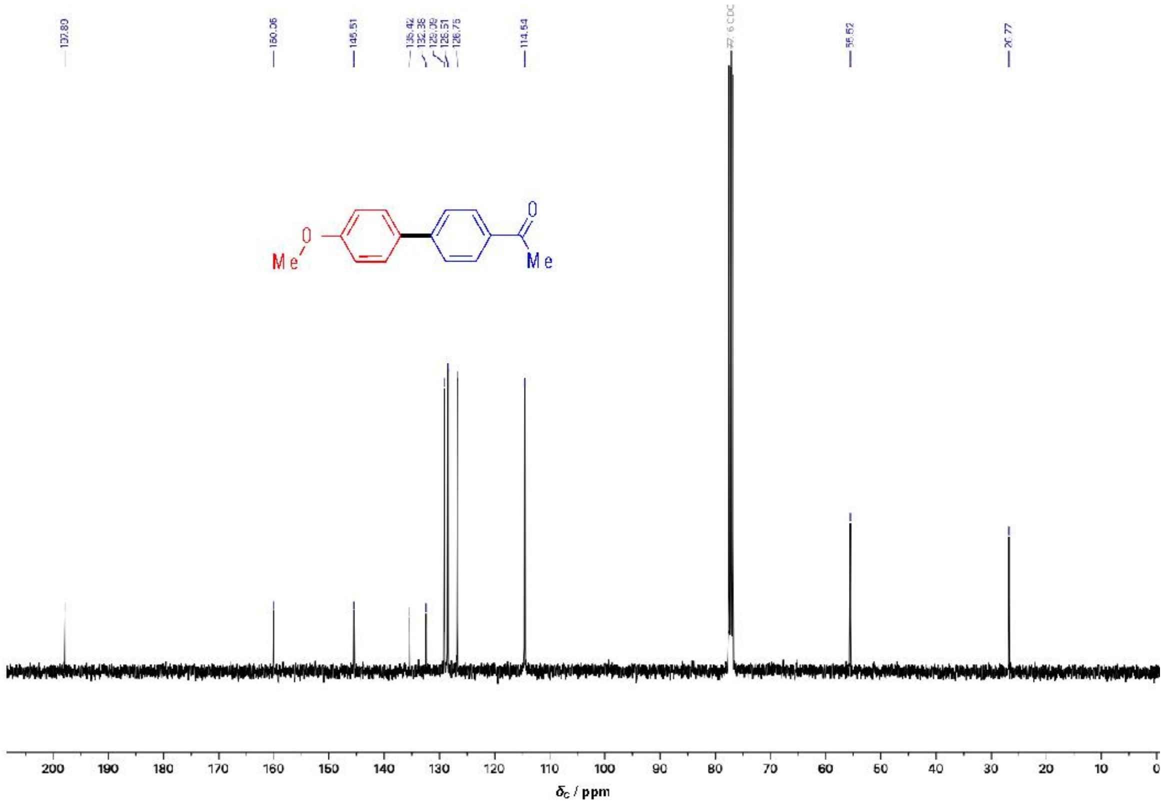

Figure S69. 101 MHz ^{13}C NMR of **3a** in $CDCl_3$

4a

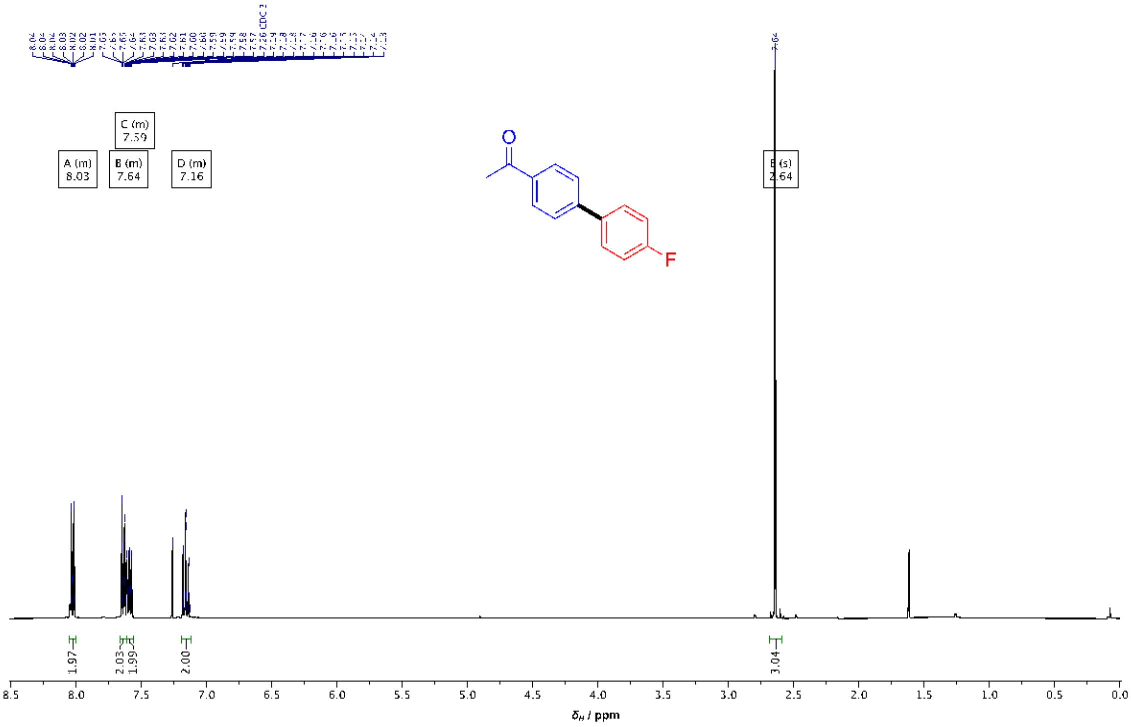

Figure S70. 400 MHz ^{1}H NMR of **4a** in $CDCl_3$

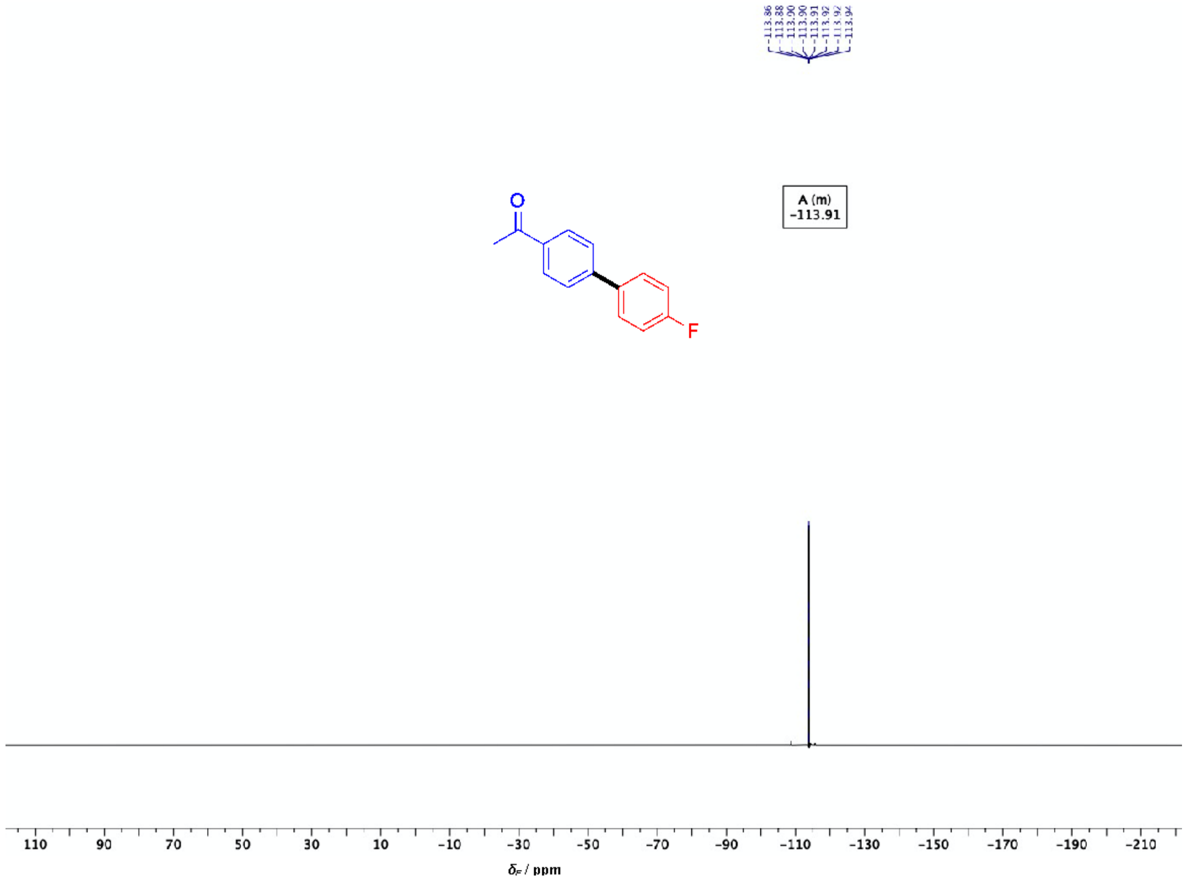

Figure S71. 376 MHz ^{19}F NMR of **4a** in $CDCl_3$

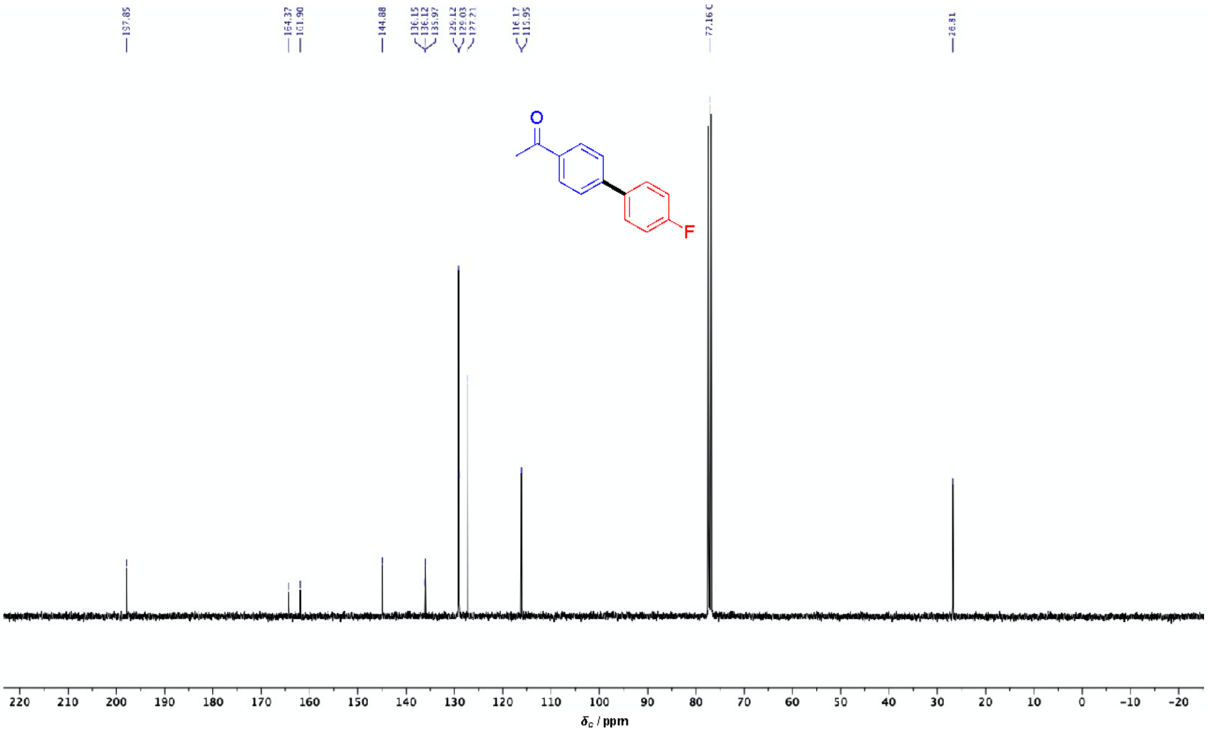

Figure S72. 101 MHz ^{13}C NMR of **4a** in $CDCl_3$

5a

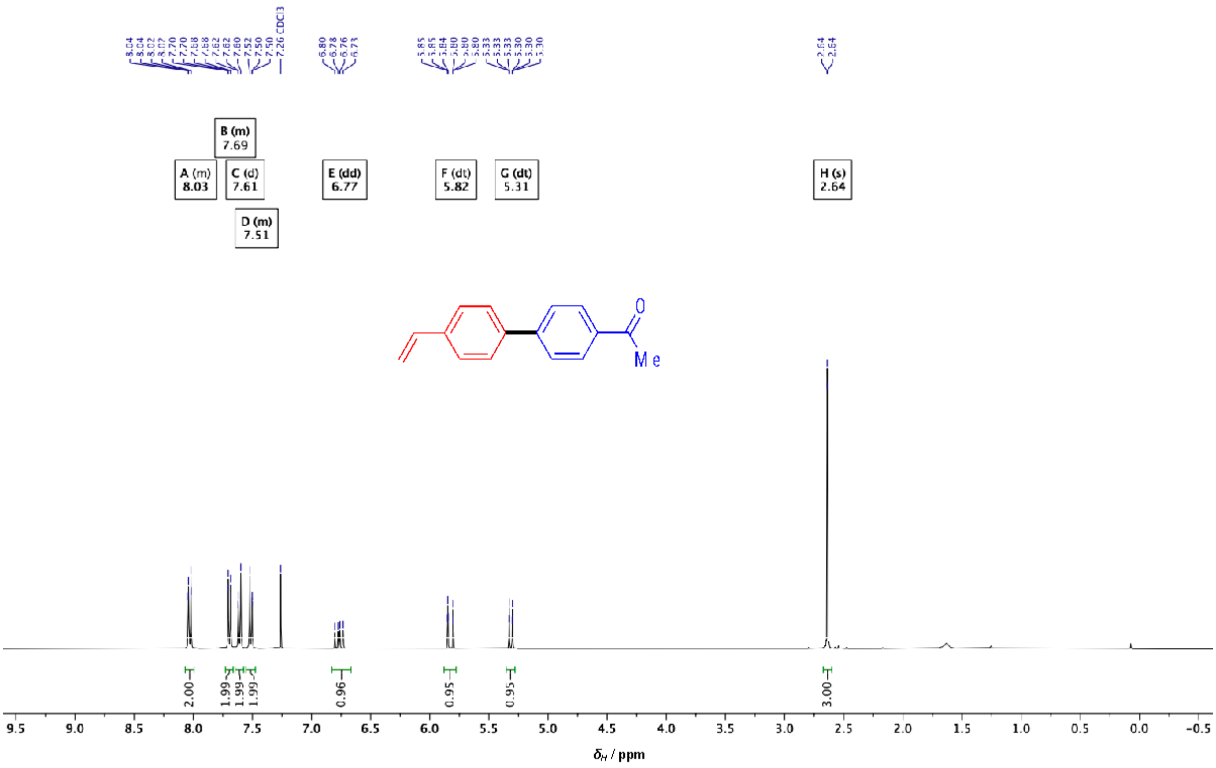

Figure S73. 400 MHz ^{1}H NMR of **5a** in $CDCl_3$

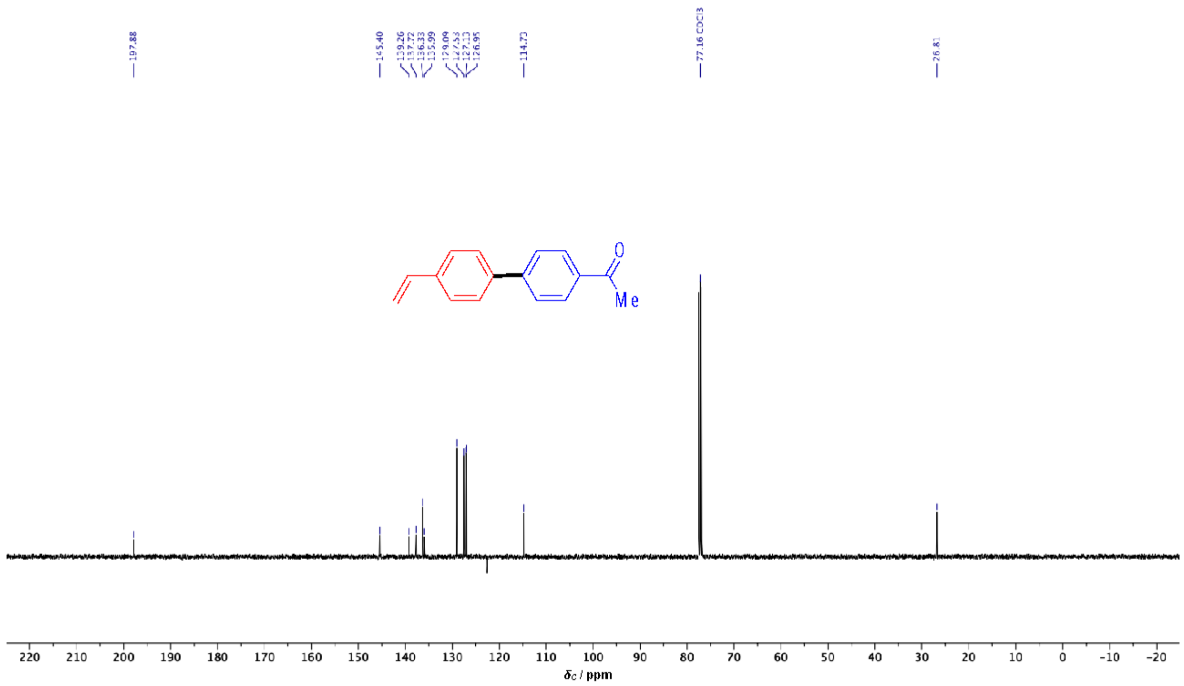

Figure S74. 101 MHz ^{13}C NMR of **5a** in $CDCl_3$

6a

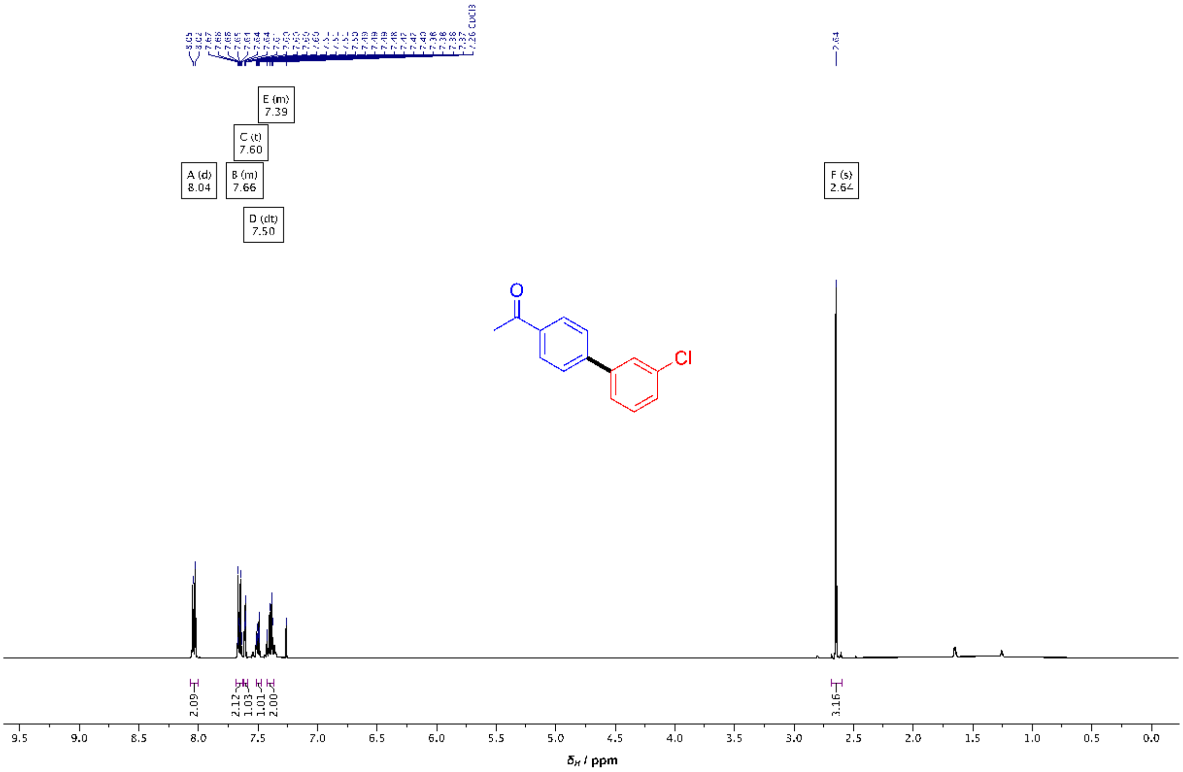

Figure S75. 400 MHz ^{1}H NMR of **6a** in $CDCl_3$

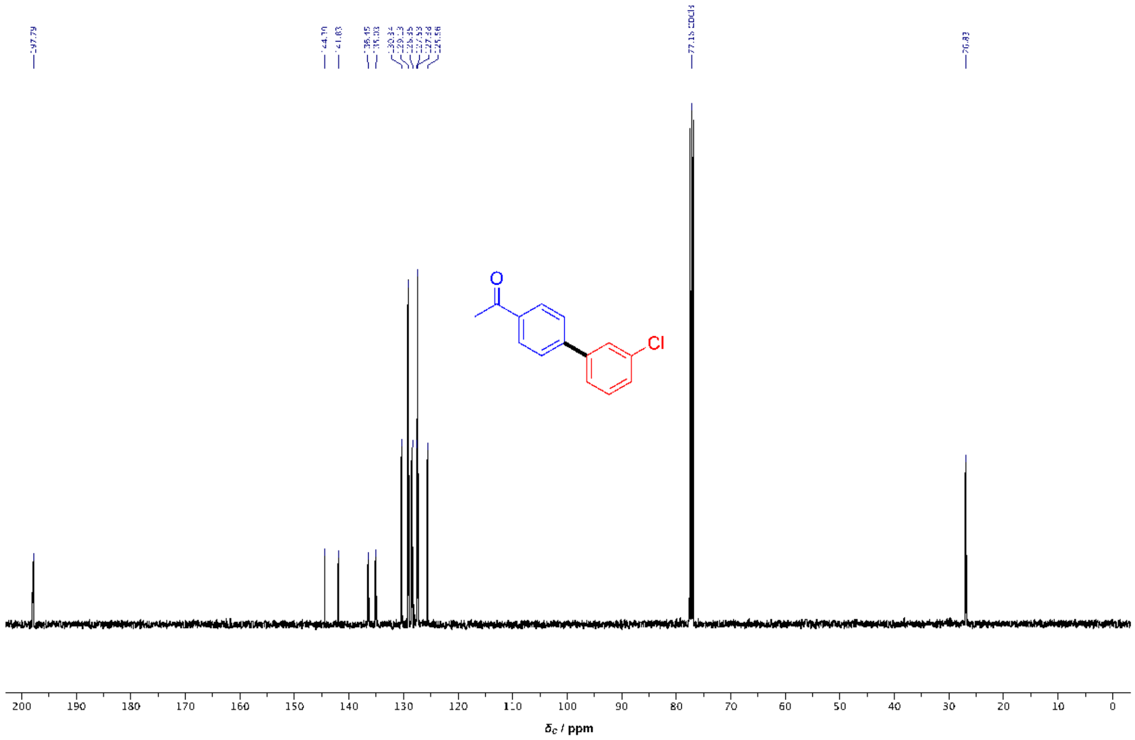

Figure S76. 101 MHz ^{13}C NMR of **6a** in $CDCl_3$

3b

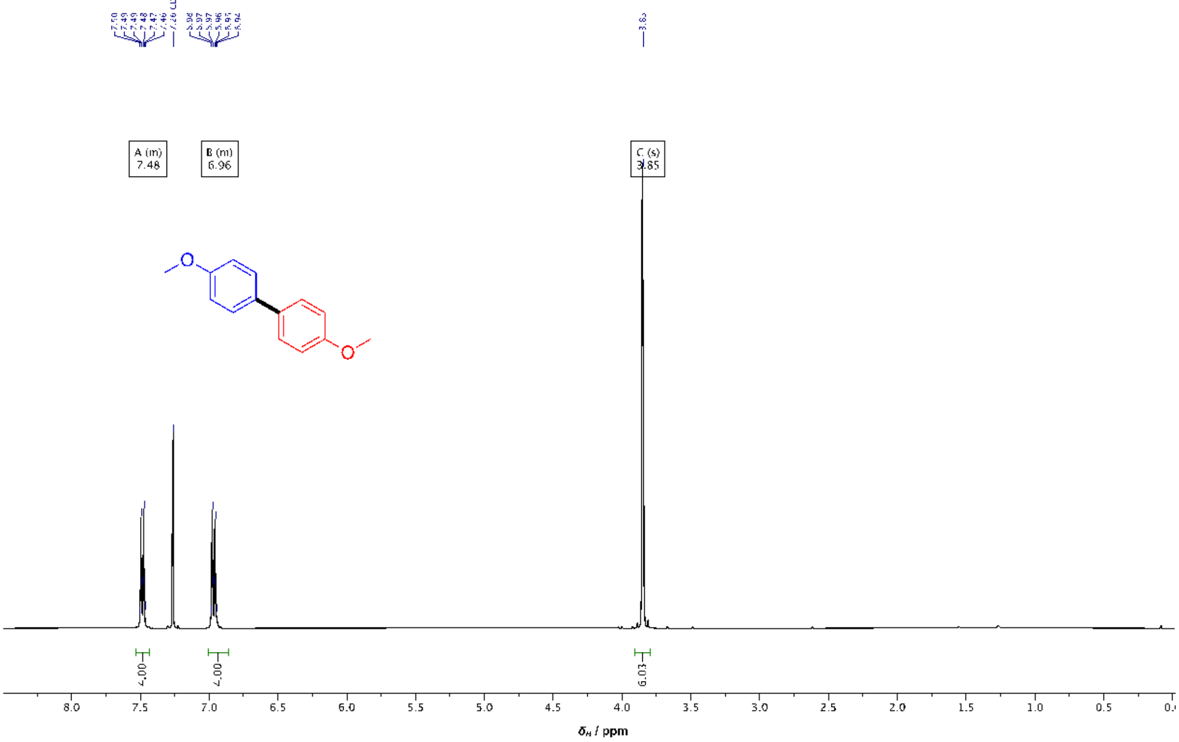

Figure S77. 400 MHz ^{1}H NMR of **3b** in $CDCl_3$

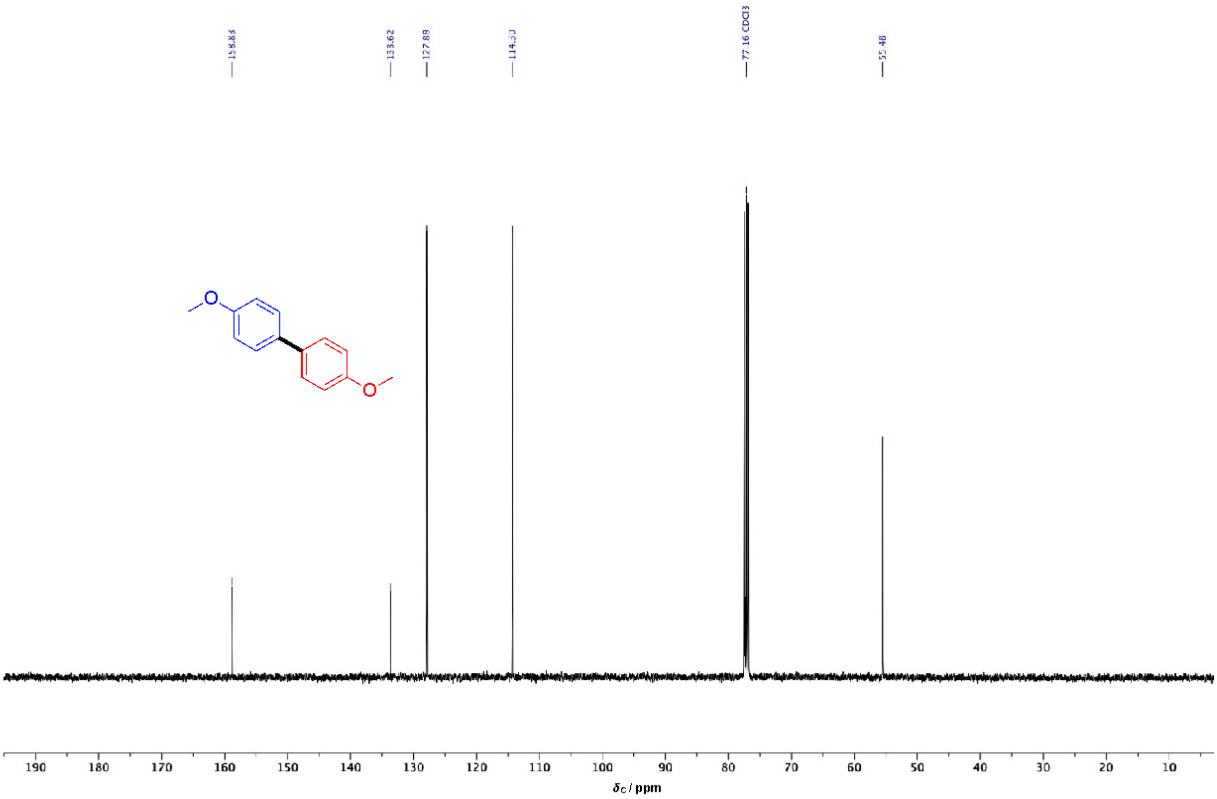

Figure S78. 101 MHz ^{13}C NMR of **3b** in $CDCl_3$

7m

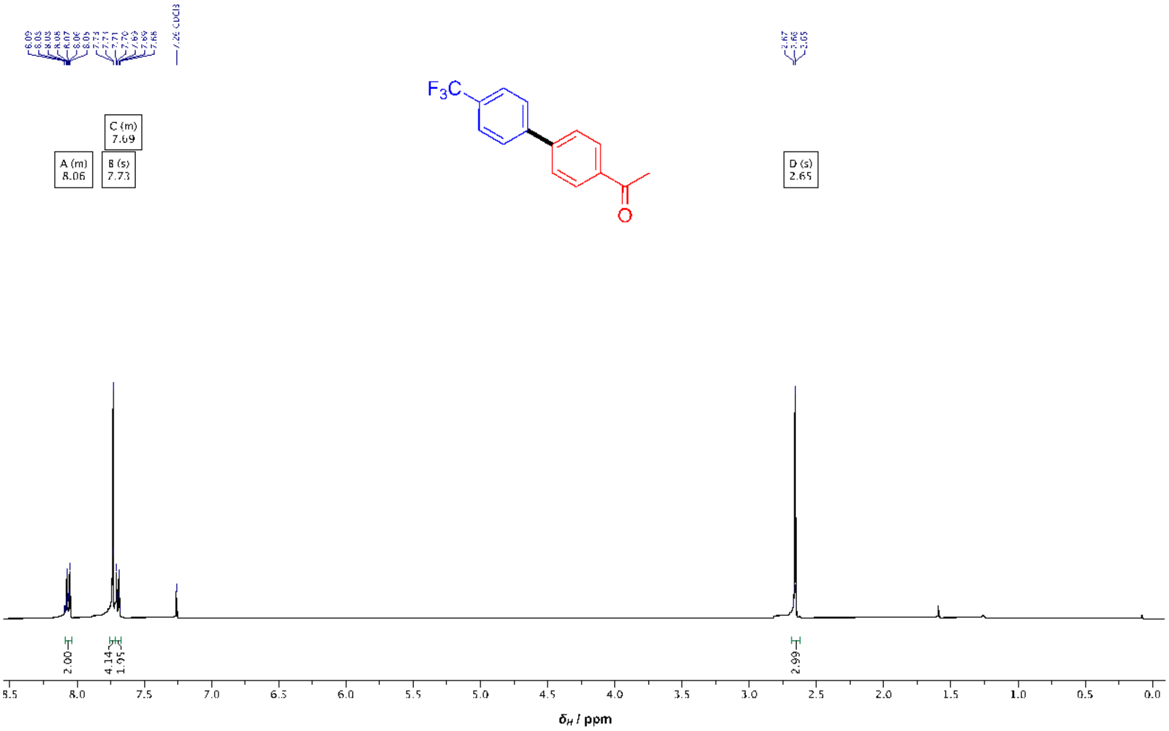

Figure S79. 400 MHz ^{1}H NMR of **7m** in $CDCl_3$

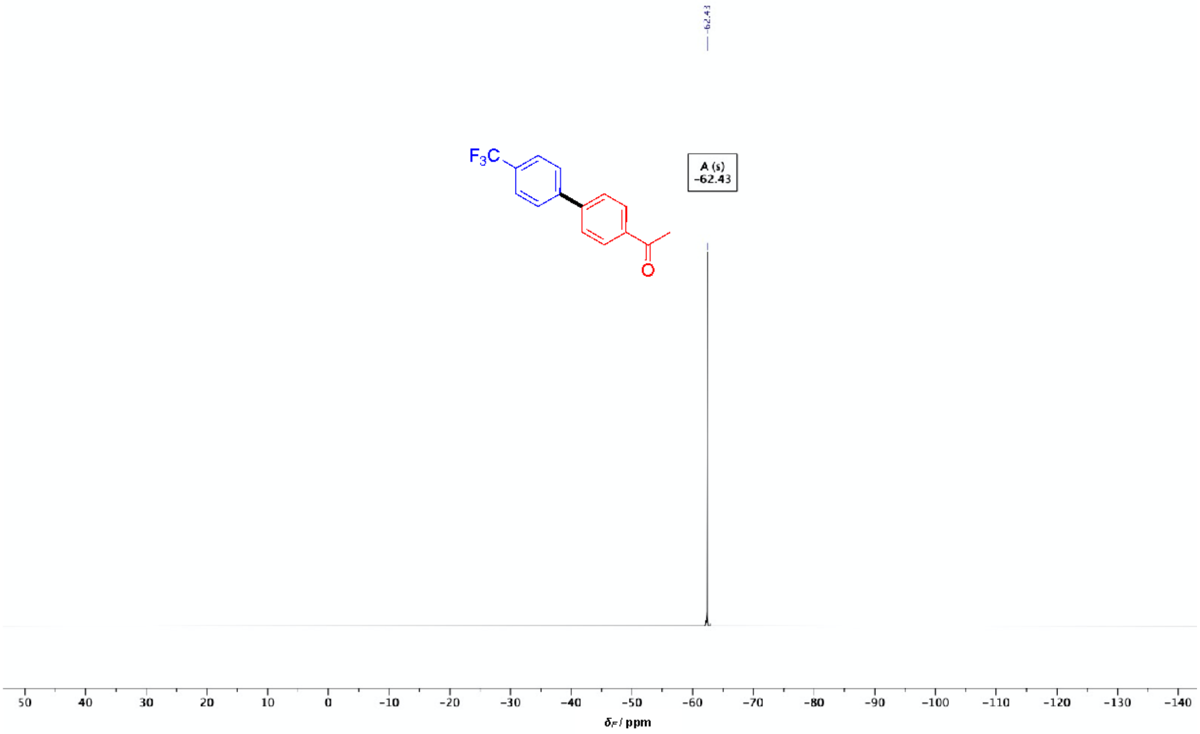

Figure S80. 376 MHz ^{19}F NMR of **7m** in $CDCl_3$

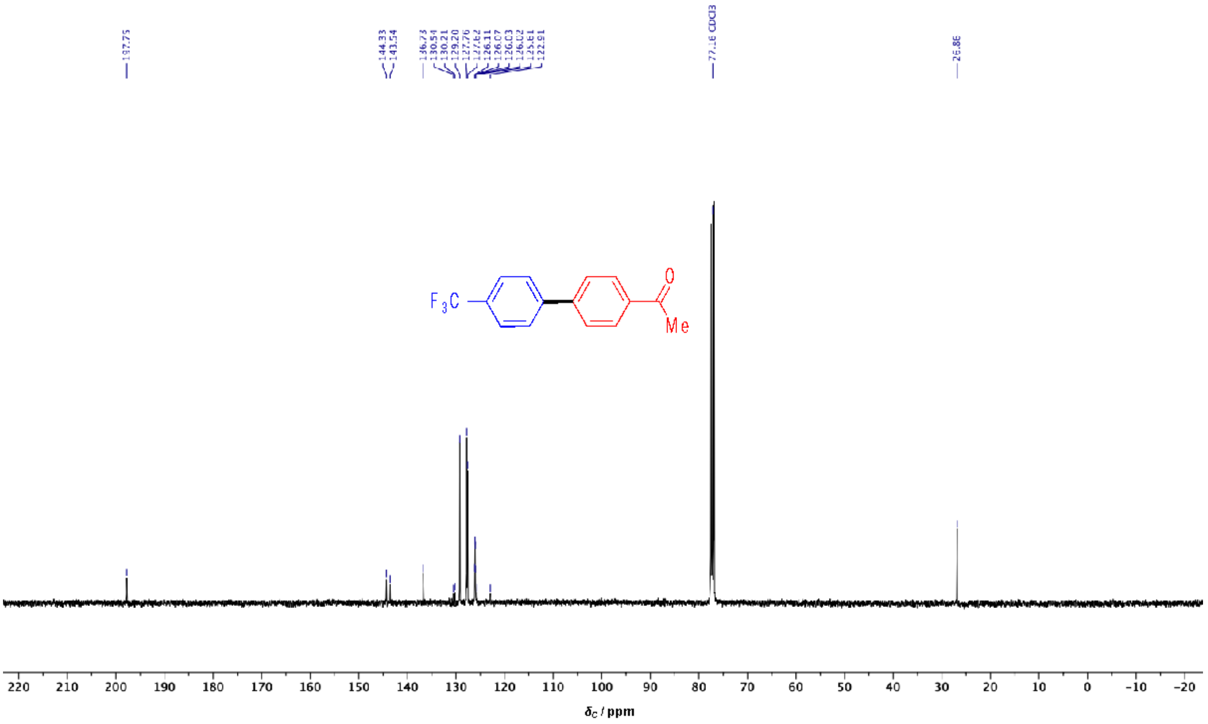

Figure S81. 101 MHz ^{13}C NMR of **7m** in $CDCl_3$

8n

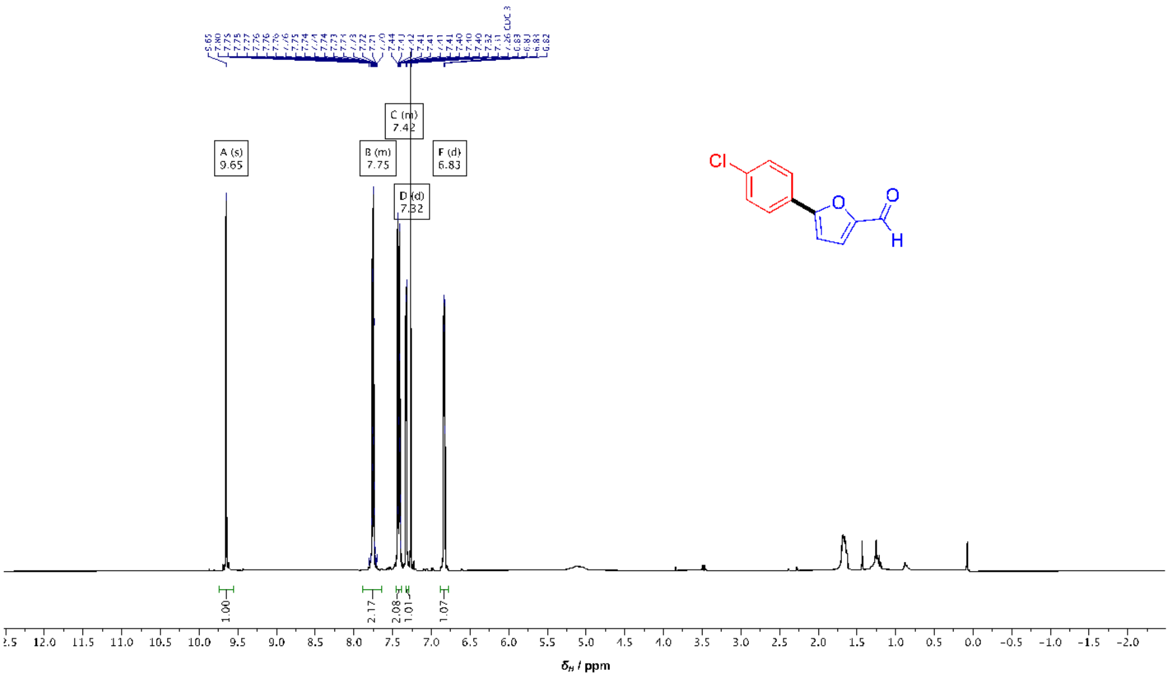

Figure S82. 400 MHz ^1H NMR of **8n** in DMSO-d_6

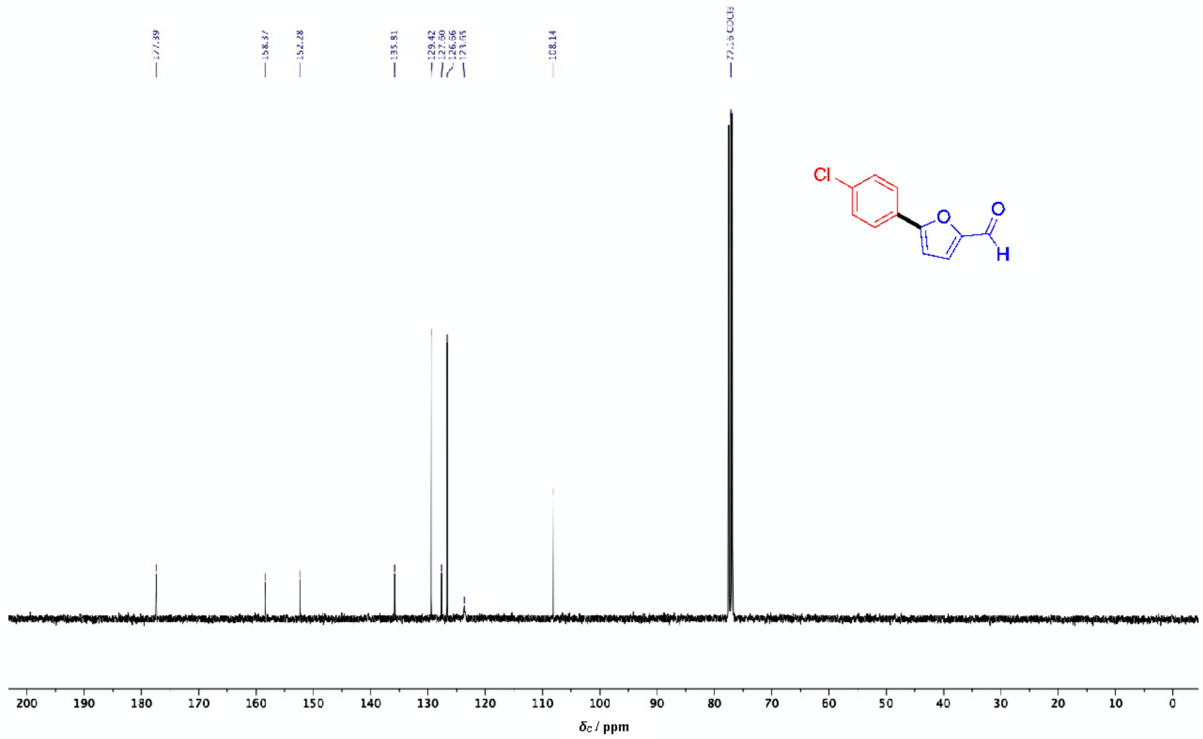

Figure S83. 101 MHz ^{13}C NMR of **8n** in DMSO-d_6

8n2

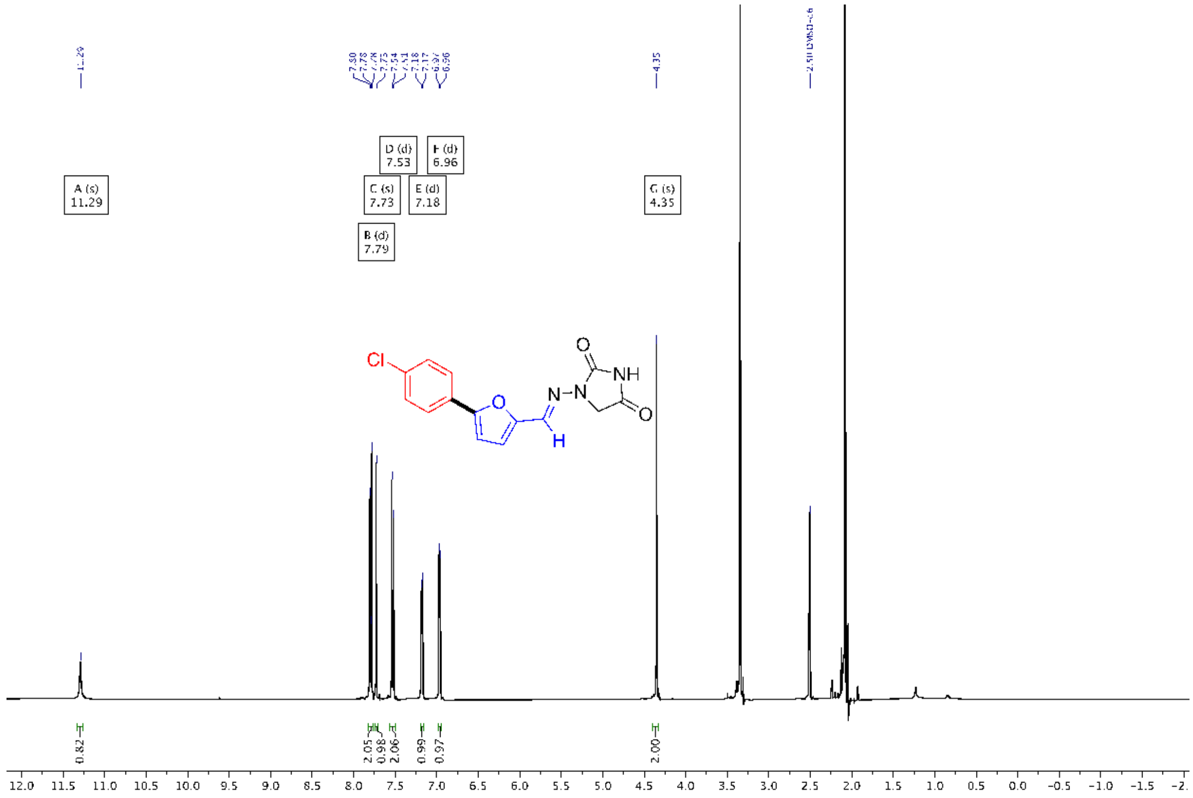

Figure S84. 400 MHz ^{1}H NMR of **8n2** in DMSO-d_6

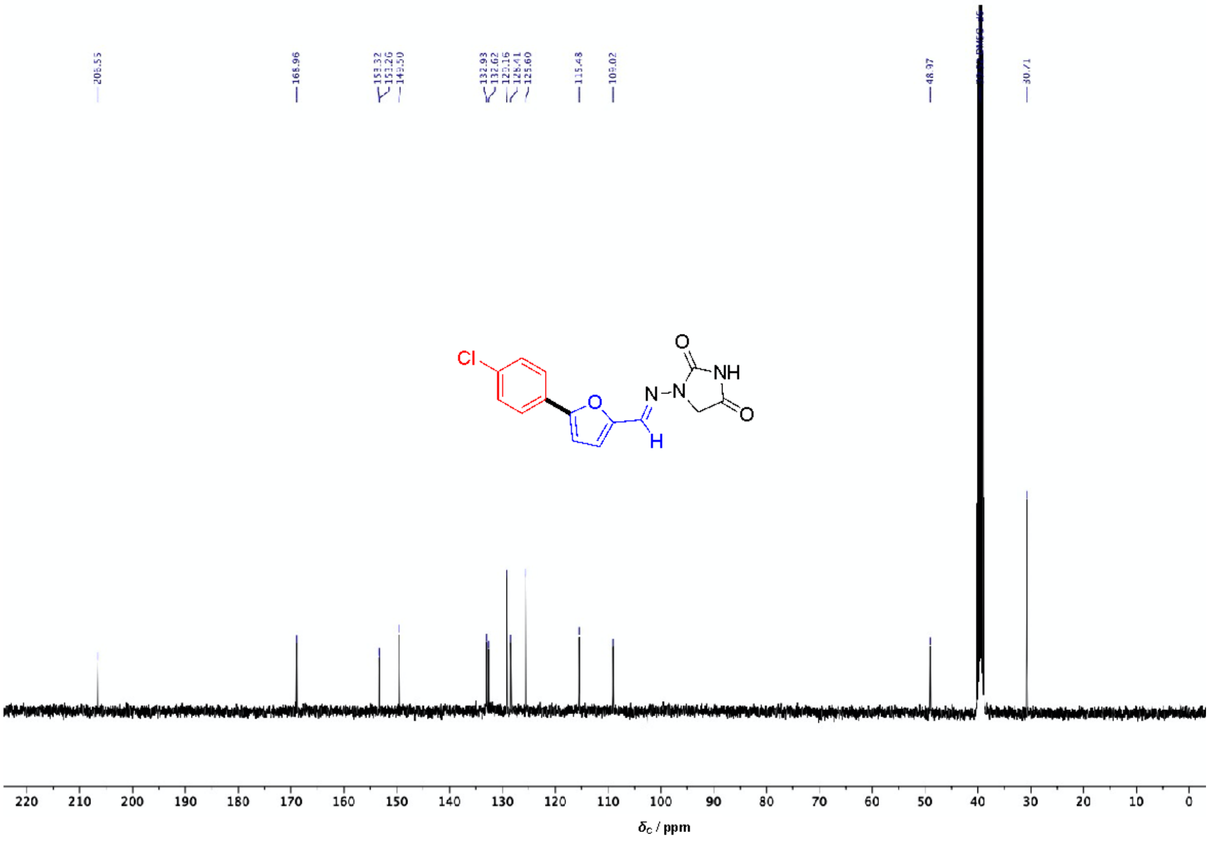

Figure S85. 101 MHz ^{13}C NMR of **8n2** in DMSO-d_6

4o

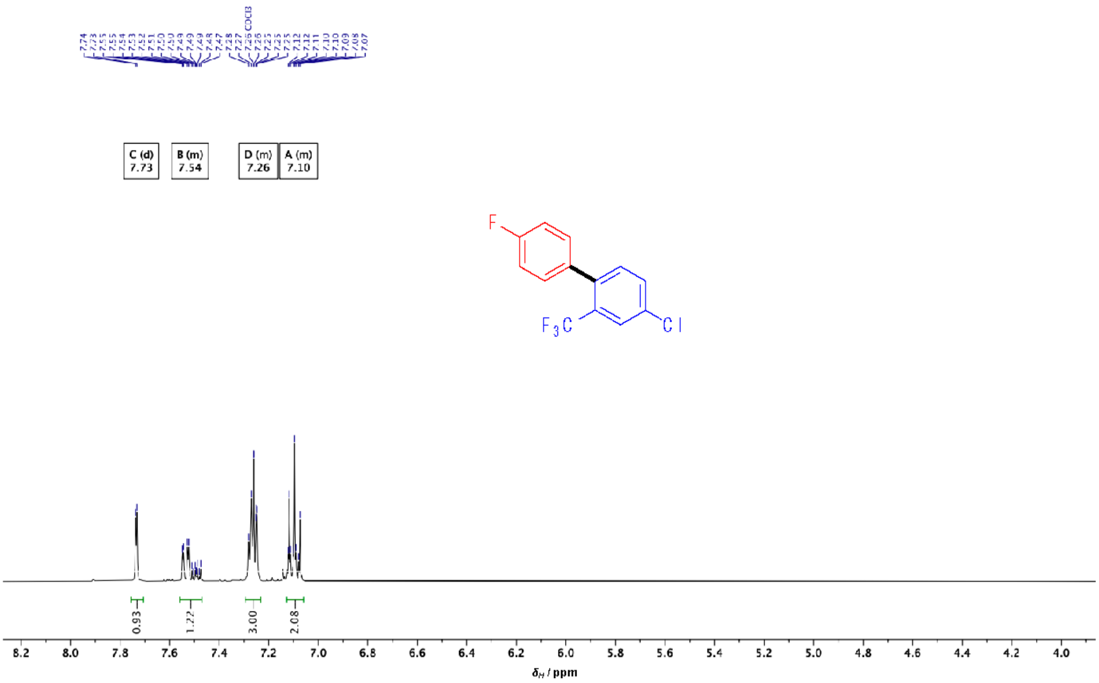

Figure S86. 400 MHz ^{1}H NMR of **4o** in $CDCl_3$

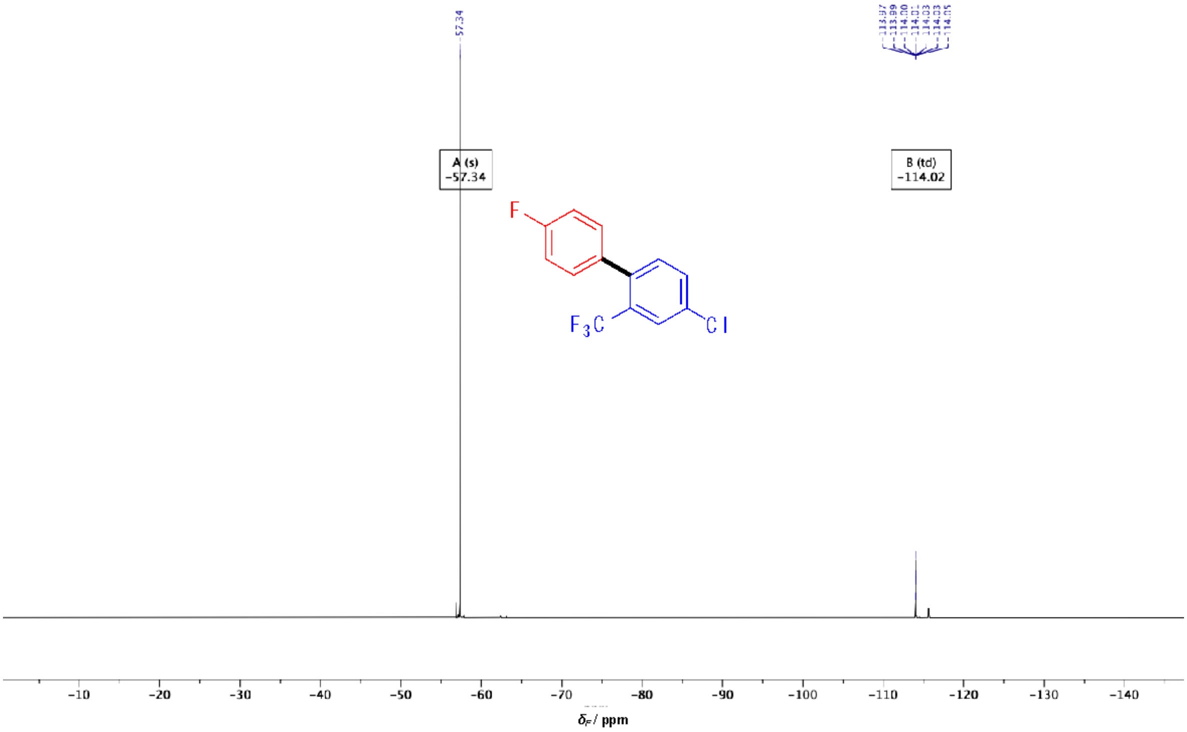

Figure S87. 376 MHz ^{19}F NMR of **4o** in $CDCl_3$

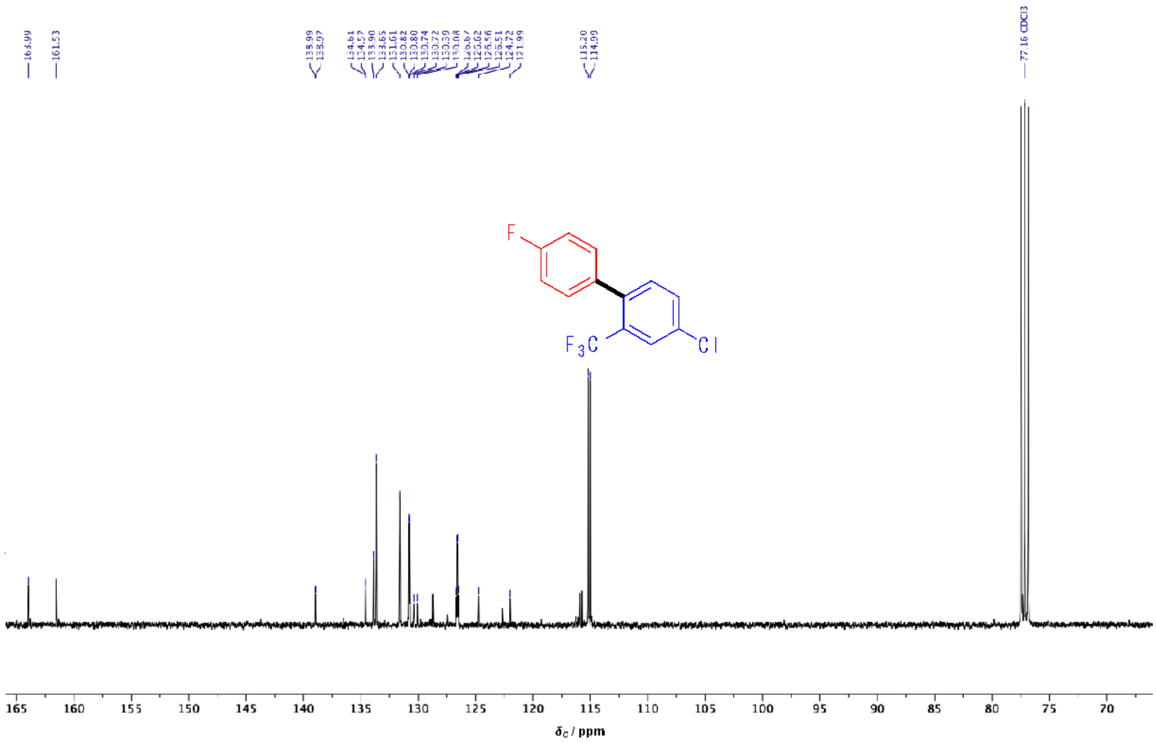

Figure S88. 101 MHz ^{13}C NMR of **7m** in $CDCl_3$

S14 XRD Data

All thermal ellipsoids are shown at 50% probability. Carbon atoms are shown in black, oxygen in red, nitrogen in blue, fluorine in dark green, chlorine light green and hydrogen in white.

1a

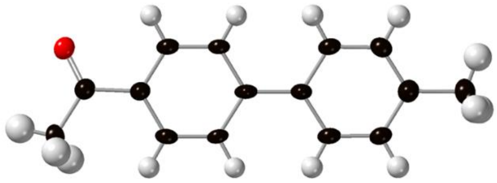

Figure S89. Crystal Structure of **1a**

Data collected, solved and refined by Adrian C. Whitwood. CCDC: 2206299; **1a** was crystallized by slow evaporation of hexane.

Table S13. Table of Crystal data and structure refinement for **1a**

Identification code	dks22016
Empirical formula	$C_{15}H_{14}O$
Formula weight	210.26
Temperature/K	110.00(10)
Crystal system	orthorhombic
Space group	$P2_12_12_1$
a/Å	5.5165(3)
b/Å	7.5009(4)
c/Å	27.2394(13)
α/°	90
β/°	90
γ/°	90
Volume/Å^3	1127.14(10)
Z	4
ρ_{calc}g/cm^3	1.239
μ/mm^{-1}	0.590
F(000)	448.0
Crystal size/mm^3	0.136 × 0.042 × 0.031
Radiation	Cu Kα (λ = 1.54184)
2Θ range for data collection/°	12.238 to 134.106
Index ranges	-6 ≤ h ≤ 6, -8 ≤ k ≤ 7, -32 ≤ l ≤ 32
Reflections collected	3775
Independent reflections	1985 [R_{int} = 0.0252, R_{sigma} = 0.0338]
Data/restraints/parameters	1985/0/147
Goodness-of-fit on F^2	1.055
Final R indexes [I>=2σ (I)]	R_1 = 0.0477, wR_2 = 0.1281
Final R indexes [all data]	R_1 = 0.0542, wR_2 = 0.1351
Largest diff. peak/hole / e Å^{-3}	0.32/-0.16
Flack parameter	-0.6(3)

1d

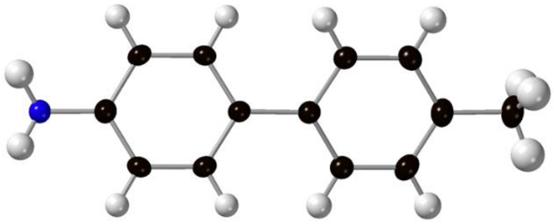

Figure S90. Crystal Structure of **1d**

Data collected, solved and refined by Adrian C. Whitwood. CCDC: 2206300; **1d** was crystallized by slow evaporation of hexane.

Table S14. Table of Crystal data and structure refinement for **1d**

Identification code	dks22008
Empirical formula	$C_{15}H_{17}N$
Formula weight	211.29
Temperature/K	110.05(10)
Crystal system	orthorhombic
Space group	Fdd2
a/Å	9.4033(3)
b/Å	16.7998(5)
c/Å	14.5691(4)
α/°	90
β/°	90
γ/°	90
Volume/Å^3	2301.53(12)
Z	8
ρ_{calc}g/cm^3	1.220
μ/mm^{-1}	0.533
F(000)	912.0
Crystal size/mm^3	0.263 × 0.172 × 0.078
Radiation	Cu Kα (λ = 1.54184)
2Θ range for data collection/°	12.38 to 133.884
Index ranges	-11 ≤ h ≤ 8, -20 ≤ k ≤ 17, -17 ≤ l ≤ 14
Reflections collected	1870
Independent reflections	901 [R_{int} = 0.0135, R_{sigma} = 0.0185]
Data/restraints/parameters	901/1/79
Goodness-of-fit on F^2	1.081
Final R indexes [I>=2σ (I)]	R_1 = 0.0307, wR_2 = 0.0855
Final R indexes [all data]	R_1 = 0.0315, wR_2 = 0.0863
Largest diff. peak/hole / e Å^{-3}	0.18/-0.15
Flack parameter	-0.3(4)

1e

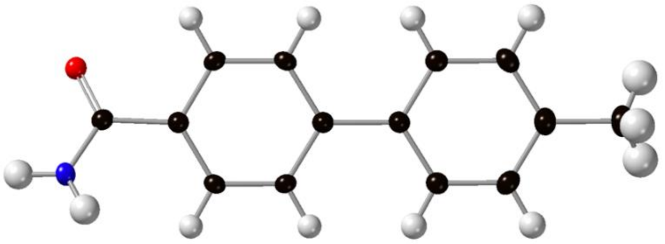

Figure S91. Crystal Structure of **1e**

Data collected, solved and refined by Adrian C. Whitwood. CCDC: 2206301; **1e** was crystallized by slow evaporation of warm MeCN.

Refinement Special details: The hydrogens of the methyl group were disordered and modelled using a 2 position riding model (AFIX 127) in a refined ratio of 0.601:0.399(19).

Table S15. Table of Crystal data and structure refinement for **1e**

Identification code	dks22017
Empirical formula	$C_{14}H_{13}NO$
Formula weight	211.25
Temperature/K	110.05(10)
Crystal system	monoclinic
Space group	$P2_1/c$
a/Å	7.9456(2)
b/Å	5.25933(15)
c/Å	26.4513(7)
α/°	90
β/°	92.993(3)
γ/°	90
Volume/Å^3	1103.86(5)
Z	4
ρ_{calc}g/cm^3	1.271
μ/mm^{-1}	0.632
F(000)	448.0
Crystal size/mm^3	0.281 × 0.091 × 0.064
Radiation	Cu Kα (λ = 1.54184)
2Θ range for data collection/°	13.408 to 134.118
Index ranges	-9 ≤ h ≤ 8, -6 ≤ k ≤ 4, -28 ≤ l ≤ 31
Reflections collected	3447
Independent reflections	1953 [R_{int} = 0.0159, R_{sigma} = 0.0237]
Data/restraints/parameters	1953/0/156
Goodness-of-fit on F^2	1.095
Final R indexes [I>=2σ (I)]	R_1 = 0.0359, wR_2 = 0.0990
Final R indexes [all data]	R_1 = 0.0434, wR_2 = 0.1061
Largest diff. peak/hole / e Å^{-3}	0.19/-0.18

8n2

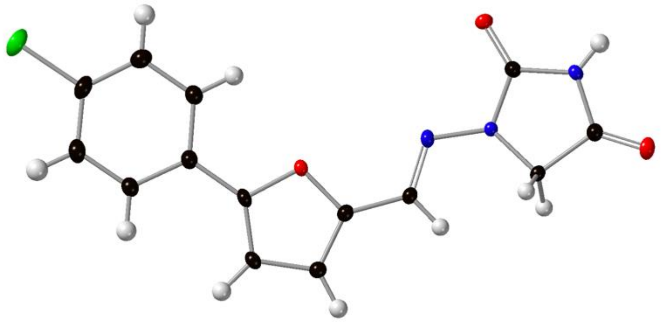

Figure S92. Crystal Structure of **8n2**

Data collected, solved and refined by Adrian C. Whitwood. CCDC: 2206302; **8n2** was crystallized by slow evaporation of MeCN.

Table S16. Table of Crystal data and structure refinement for **8n2**

Identification code	dks22015a
Empirical formula	$C_{14}H_{10}N_3O_3Cl$
Formula weight	303.70
Temperature/K	110.00(10)
Crystal system	orthorhombic
Space group	Pbca
a/Å	7.48537(12)
b/Å	12.15787(16)
c/Å	57.8150(7)
α/°	90
β/°	90
γ/°	90
Volume/Å^3	5261.52(13)
Z	16
ρ_{calc}g/cm^3	1.534
μ/mm^{-1}	2.717
F(000)	2496.0
Crystal size/mm^3	0.132 × 0.111 × 0.02
Radiation	Cu Kα (λ = 1.54184)
2Θ range for data collection/°	9.178 to 134.15
Index ranges	-6 ≤ h ≤ 8, -14 ≤ k ≤ 12, -66 ≤ l ≤ 69
Reflections collected	26935
Independent reflections	4686 [R_{int} = 0.0354, R_{sigma} = 0.0269]
Data/restraints/parameters	4686/0/387
Goodness-of-fit on F^2	1.044
Final R indexes [I>=2σ (I)]	R_1 = 0.0316, wR_2 = 0.0706
Final R indexes [all data]	R_1 = 0.0387, wR_2 = 0.0735
Largest diff. peak/hole / e Å^{-3}	0.19/-0.24

S15 References

1. CrysAlisPro, Oxford Diffraction Ltd. Version 1.171.34.41.
2. G. Sheldrick, *Acta Crystallogr. A,* 2015, **71**, 3–8.
3. G. Sheldrick, *Acta Crystallogr. C,* 2015, **71**, 3–8.
4. O. V. Dolomanov, L. J. Bourhis, R. J. Gildea, J. A. K. Howard and H. Puschmann, *J. Appl. Cryst.*, 2009, **42**, 339–341.
5. B. O. Okesola and D. K. Smith, *Chem. Commun*., 2013, **49**, 11164–11166.
6. D. J. Cornwell, B. O. Okesola and D. K. Smith, *Soft Matter*, 2013, **9**, 8730–8736
7. C. C. Piras and D. K. Smith, *Chem. Eur. J.*, 2021, **27**, 14527–14534
8. T. Ichikawa, M. Netsu, M. Mizuno, T. Mizusaki, Y. Takagi, Y. Sawama, Y. Monguchi and H. Sajiki, *Adv.Synth.Catal.,* 2017, **359**, 2269 –2279.
9. P. Wessig and S. Krebs, *Eur. J. Org. Chem.*, 2021, **2021**, 6367–6374.
10. A. Ohtsuki, K. Yanagisawa, T. Furukawa, M. Tobisu and N. Chatani, *J. Org. Chem.*, 2016, **81**, 9409−9414.
11. S. Kamio, I. Kageyuki, I. Osaka and H. Yoshida, *Chem. Comm.*, 2019, **55**, 2624–2627.
12. Y. Zhao, C. Yu, W. Liang, I. L. Atodiresei, F. W. Paterau, *Chem. Commun.*, 2022, **58**, 2846–2849.
13. Y. Luan, T. Zhang, W. Yao, K. Lu, L. Kong, Y. Lin and M. Ye, *J. Am. Chem. Soc.* 2017, **139**, 5, 1786–1789.
14. A. Shiozuka, K. Sekine and Y. Kuninobu, *Org. Lett.*, 2021, **23**, 4774−4778.
15. P. Y. Choy, O. Y. Yuen, M. P. Leung, W. K. Chow, F. Y. Kwong, *Eur. J. Org. Chem.*, 2020, **2020**, 2846–2853
16. P. Slavík, D. W. Kurka and D. K. Smith, *Chem. Sci.*, 2018, **9**, 8673–8681
17. C. Diebold, J. M. Becht, J. Lu, P. H. Toy and C. Le Drian, *Eur. J. Org. Chem.*, 2012, **2012**, 893–896.
18. Y. Liu, T. Scattolin, A. Gobbo, M. Beliš, K. Van Hecke, S. P. Nolan and C. S. J. Cazin, *Eur. J. Inorg. Chem.*, 2022, **2022**, e202100840.
19. W. Shih, Y. Chiang, Q. Wang, M. Wu, G. P. A. Yap, L. Zhao and T. Ong, O*rganometallics*, 2017, **36**, 21, 4287–4297.
20. J. M. A. Miguez, L. A. Adrio, A. Sousa-Pedrares, J. M. Vila and K. K. M. Hii, *J. Org. Chem.*, 2007, **72**, 7771–7774.
21. J. M. Gil-Negrete, J. Pérez Sestelo and L. A. Sarandeses, *Chem. Comm.*, 2018, **54**, 1453–1456.

22. B. T. Luo, H. Liu, Z. J. Lin, J. Jiang, D. S. Shen, R. Z. Liu, Z. Ke and F. S. Liu, *Organometallics*, 2015, **34**, 4881−4894.
23. Z. Lin, F. Deyu, R. Lu, Z. Xiaoli, L. Chun, Z. Danyu, W. Yanjie, C. Wenna, G. Junfu, C. Peiwei, L. Liping, L. Baokun, W. Yue, C. Yong and L. Yuqian, CNIPA, CN111620918, 2020
24. M. M. S. Andrade, Í F. Protti, V. G. Maltarollo, Y. F. G. da Costa, W. G. de Moraes, N. F. Moreira, G. G. Garcia, G. F. Caran, F. M. Ottoni, R. J. Alves, C. P. S. Moreira, H. R. Martins, M. S. Alves and R. B. de Oliveira, *Med. Chem. Res.,* 2021, **30**, 1074–1086
25. I. Ali, B. Siyo, Z. Hassan, I. Malik, I Ullah, A. Ali, M. Nawaz, J. Iqbal, T. Patonay, A. Villinger and P. Langer, *J. Fluor. Chem.*, 2013, **145**, 18–34.
26. N. W. J. Scott, M. J. Ford, N. Jeddi, A. Eyles, L. Simon, A. C. Whitwood, T. Tanner, C. E. Willans and I. J. S. Fairlamb, *J. Am. Chem. Soc.* 2021, **143**, 9682−9693.
